# DNA Damage and Transcriptional Changes in the Gills of *Mytilus galloprovincialis* Exposed to Nanomolar Doses of Combined Metal Salts (Cd, Cu, Hg)

**DOI:** 10.1371/journal.pone.0054602

**Published:** 2013-01-23

**Authors:** Laura Varotto, Stefania Domeneghetti, Umberto Rosani, Chiara Manfrin, Miren P. Cajaraville, Stefano Raccanelli, Alberto Pallavicini, Paola Venier

**Affiliations:** 1 Department of Biology, University of Padova, Padova, Italy; 2 Department of Life Sciences, University of Trieste, Trieste, Italy; 3 Department of Zoology & Cell Biology, University of the Basque Country UPV/EHU, Bilbao, Basque Country, Spain; 4 Consorzio INCA Marghera, Venezia, Italy; Catalan Institute for Water Research (ICRA), Spain

## Abstract

Aiming at an integrated and mechanistic view of the early biological effects of selected metals in the marine sentinel organism *Mytilus galloprovincialis*, we exposed mussels for 48 hours to 50, 100 and 200 nM solutions of equimolar Cd, Cu and Hg salts and measured cytological and molecular biomarkers in parallel. Focusing on the mussel gills, first target of toxic water contaminants and actively proliferating tissue, we detected significant dose-related increases of cells with micronuclei and other nuclear abnormalities in the treated mussels, with differences in the bioconcentration of the three metals determined in the mussel flesh by atomic absorption spectrometry. Gene expression profiles, determined in the same individual gills in parallel, revealed some transcriptional changes at the 50 nM dose, and substantial increases of differentially expressed genes at the 100 and 200 nM doses, with roughly similar amounts of up- and down-regulated genes. The functional annotation of gill transcripts with consistent expression trends and significantly altered at least in one dose point disclosed the complexity of the induced cell response. The most evident transcriptional changes concerned protein synthesis and turnover, ion homeostasis, cell cycle regulation and apoptosis, and intracellular trafficking (transcript sequences denoting heat shock proteins, metal binding thioneins, sequestosome 1 and proteasome subunits, and GADD45 exemplify up-regulated genes while transcript sequences denoting actin, tubulins and the apoptosis inhibitor 1 exemplify down-regulated genes). Overall, nanomolar doses of co-occurring free metal ions have induced significant structural and functional changes in the mussel gills: the intensity of response to the stimulus measured in laboratory supports the additional validation of molecular markers of metal exposure to be used in Mussel Watch programs.

## Introduction

Edible marine bivalves of the genus *Mytilus* have a long history as biosensors of coastal water pollution [1] but they are also attractive for genetic selection [2], biotechnological applications [3] and functional ecology studies [4]–[7].

Both the levels of contaminants accumulated by filter-feeding in the mussel tissues and related biological effects are widely documented, and used to assess the quality of the coastal marine waters and health risks [8], [9]. Despite a general consensus on the integration of different measures, biomarkers and biomonitoring criteria are a matter of study [10], [11] and the current ‘omics’ sciences greatly enlarge the repertoire of the potential indicators to be considered for validation and standardization [12], [13].

The surveillance of early induced biological alterations in sentinel species such as mussels can disclose the impact of water contaminants not usually analyzed. However, the induction of toxic effects strongly depends on the substance properties, water chemistry and functional traits of the target organisms, not only different life stages and typical behaviours but also circadian and tidal rhythms of gene expression. Hence, careful evaluation of the cause-effect relationships and identification of reliable biomarkers in various exposure sceneries are crucial for advancing in environmental toxicology and risk assessment.

Essential and non-essential metals [14] can occur in various mixtures at doses threatening the human and ecosystem health because of natural sources or in the proximity of urbanized coasts and harbours [15], [16]. In the coastal transition waters, geochemical conditions such as pH and organic matter components influence the element speciation and the affinity constants for ligand binding, with the most labile metal fractions and free ions more likely bioavailable and related to toxic effects [17], [18]. Also in aquatic animals with different physiological traits, the element speciation and affinity constants for biotic ligands are crucial determinants of metal transport, intracellular uptake, reaction to critical targets and detoxification pathways.

According to the Lewis acid classification, hard metal ions of class A preferentially form ionic bonds and complexes with oxygen donors, soft metal ions of class B such as Cu(I), Cd, Hg preferentially form covalent bonds with sulphur or nitrogen donors whereas borderline metal ions such as Cu(II) form rather stable complexes with O- and S- or N- donors. Given the stable bounds with their ligands (metallothioneins, MTs, and other cell proteins) borderline and class B metal ions are difficult to eliminate and more likely cause membrane damage and other detrimental effects in the living organisms [19], [20].

Bivalve molluscs display metal absorption rate constants comparable to crustaceans and ten times higher than in fish species (decreasing in the order Ag>Hg>Zn>Cd>Co>Cr(III)>Cs and consistent with class B reactivity) whereas weight-corrected elimination rate constants seem relatively constant across metals and animal species [21]. The efficient metal absorption observed in bivalves is largely determined by species-specific and temperature-dependent filtration rates: according to the dissolved and particulate metal amounts, the uptake mainly occurs through the gills and digestive tube, and is also influenced by selective regulatory mechanisms for essential redox elements such as copper, involved in the hemocyanin-mediated oxygen transport [22], [23]. Passive diffusion of lipophilic metal compounds/complexes, transfer through membrane ion channels and transporters as well as the formation of endocytic vesicles have a role in the metal influx. Bivalve plasma components and haemocytes can drive metal ions into the soft tissues and mediate their accumulation in diverse cell types, cytoplasmic organelles and granules, and even in the bissal threads and shells wherein metal ions can replace calcium in the carbonate complex [15], [22], [24]. As regards the soft mussel tissues, gills have been reported to accumulate the highest or comparable metal levels than digestive gland [18], [23]. In filibranch bivalves, two gill lamellae per demibranch divide the pallial cavity into inhalant and exhalant chambers; each one is arranged in rows of ciliated filaments enclosing haemolymph sinuses and consisting of ciliated/non-ciliated epithelial cells, endothelial-like and mucous cells [25].

Metal uptake occurs particularly in the mucus-rich abfrontal (distal third of the gill lamellae) and laterofrontal regions of the gill filaments. For instance, Cd can enter the columnar epithelial cells through Ca channels, can be incorporated into lysosomes, transported in vesicles and basally exocytosed into the haemolymph. Depending on the element and its intracellular concentration, metals can be released also from haemocytes, digestive cells, nephrocytes, and eventually eliminated with faeces and urine [22], [26]. Following mussel exposure to 100 µg/L Hg for 30–60 days, metal accumulation and release from the apical and basal regions of epithelial gill cells have been reported, and a decrease in metal accumulation due for instance to adaptive cell renewal during long-term exposure has been hypothesized [27].

Depending on the exposure history and other environmental variables, the subcellular distribution and body burden of essential and non-essential metals in bivalve species depend on regulative processes and adjustments aimed to maintain the cell ion homeostasis, and to avoid the potential damage due to excessive metal concentrations. Continuous or intermittent exposure of clams and mussels to Cd, Cu, Hg has been associated to increased metal binding to thioneins, autophagy and lysosome destabilization, accumulation of metal-rich granules and age pigment lipofuscin [28]–[30]. Actually, the removal of the metal ions from cytosol by increased MT synthesis, compartmentalization into the endo-lysosomal system and mineralized granules can enhance the uptake and accumulation processes. Autometallography, a technique detecting metal ions by transforming them into insoluble sulfides, have confirmed the localization of Cd, Hg, Ag, Pb, Cu and Zn in either digestive cells, gill cells or haemocytes of mussels [22], [26], [27].

In general, controlled laboratory studies support the identification of toxicant-induced biological changes and instruct the validation of biomarkers, as pollution warning signals, in the real environment. However, such general objective is more difficult to disentangle in the case of complex contaminant mixtures and the effects of combined toxic metals have been only occasionally studied by using a transcriptomic approach in the *Mytilus* species.

The present study aims to extend, and reinforce with other measures, the DNA microarray data concerning the digestive gland of winter mussels intentionally exposed for two days to the 100 nM nominal dose of combined soluble salts of Cd, Cu and Hg, already known to induce MT proteins and DNA damage in the absence of manifest toxicity [31], [32]. Thus, our objective was to evaluate the relative robustness of traditional biomarkers, i.e. the frequency of cells with micronuclei (MN) and other nuclear abnormalities (NA), and transcriptional markers of metal exposure in the mussel gills.

Copper is a nutrient in trace amounts and commonly occurs in water as redox-active cation mostly associated to organic matter (used in antifouling, fungicide and larvicide products, for instance against mosquito-borne diseases) whereas both Cd and Hg are not essential for life [33], [34]. Cadmium mostly occurs in divalent oxidation state and is largely present in manufactured products. It is a model toxicant in aquatic species also known as animal and human carcinogen. Mercury is a volatile element whose global distribution depends on both natural and anthropogenic sources; highly soluble and easily released from its aqueous divalent cations, it can be biologically transformed in organic (primarily alkyl) compounds able to cause neurotoxic effects following bioaccumulation and biomagnifications. In humans, the half-life of Hg and Cd was estimated in 60–70 days and 10–20 years, respectively [33]. The maximum admissible level of metals in bivalve mollusc food stuff is 1.5 mg/kg wet weight (ww), with more restrictive values for Cd and Hg: 1.0 and 0.1 mg/kg ww, respectively [35], [36] whereas 960–1550 µg/L, 122–480 µg/L and 67–2510 µg/L are the empirical LC50 ranges reported for *M. edulis* after 96 h of exposure to waterborne Cd, Cu and Hg, respectively [37].

In the following sections, we present and discuss experimental data relating to mussels exposed for two days to 50–200 nM doses of combined Cd, Cu and Hg salts, renewed every 12 hours together with the sea water and food. The applied dose range exemplifies metal concentrations which could be measured in contaminated coastal sites, for instance in the case of sediment resuspension and metal mobilization from the particulate matter when the increased metal bioavailability can lead to bioaccumulation and biomagnification phenomena [38].Actually, exceptional Hg contamination has occurred in sea food stuff with methyl mercury levels of 9–24 µg/g in fishes [39]. Despite the Hg levels detected in bivalves are usually significantly smaller, for instance 27.3±12.4 and 66.6±23.5 µg/Kg of Hg have been reported in canned mussels and cockles, new contamination events and the peculiar physico-chemical behaviour of Hg can change these figures [40]–[42].

## Materials and Methods

### Ethics Statement

The Mediterranean mussels used in this study (*Mytilus galloprovincialis* Lmk. 1819, common and not endangered invertebrate species) were obtained by courtesy of local producers (Mitilpesca and C.a.m. Srl, Venice lagoon area, Italy). No permits were required for the described study, which complied with all relevant regulations, and all efforts were made to minimize possible animal suffering.

### Mussel Samplings, Treatments and Gill Processing

Adult mussels of mixed sex were collected from a farming site offshore Alberoni in late spring when no interference of massive spawning is expected. Following acclimatization at 18±1°C in 32 ‰ artificial sea water with commercial food (Coralife® Sera, Heinsberg, Germany) mussels of average size (shell length 4.93±0.17 cm) were placed in three plastic tanks (5 mussels/tank, 1 litre sea water/mussel) and exposed for 48 hours to equimolar mixtures of Cd, Cu and Hg chloride salts (final concentration 50, 100 and 200 nM, 11–16 June). The applied dose range of metal salts is equivalent to 5.6–22.5, 3.2–12.7 and 10.0–40.1 µg/L of Cd, Cu and Hg, respectively. Water, food and metal salts were changed every 12 h during the treatment, and the mussel reactivity was regularly monitored (i.e. valve movement, bissus and faeces production). Five control mussels were maintained in a fourth tank in parallel.

At the dissection, the mussels showed intermediate gonad development. Individual half-gill was rapidly rinsed in physiological solution, frozen in liquid nitrogen and stored at –80°C until RNA purification and DNA microarray analysis. The other half-gill was subjected to mild enzymatic digestion (dispase II 0.6 U/ml Alsever, 10–15 min at room temperature). The cell suspension was subsequently filtered at 280 and 100 µm, washed twice in Alsever (200×g at 10°C) and centrifuged on slide at suitable cell density with a Shandon Cytospin (800 rpm, 2 min) in agreement to earlier experience [43], [44]. Finally, the gill cell preparations were fixed in iced methanol, stained with 6% Giemsa and make permanent in DPX mountant (Fluka).

The treatment of acclimatized mussels was repeated twice in the following year at the same temperature and salinity conditions (18±1°C, 32 ‰) using marketed mussels with ripe or depleted gonads (19–21 January, average shell length 7.72±0.33 cm) and offshore mussels with intermediate gonad development, collected again in late spring (11–16 June, average shell length 5.94±0.21 cm). With the former group we aimed to estimate the metal bioaccumulation in whole mussel tissues and, with the latter, we measured the frequency of cytogenetic alterations in opposite seasonal conditions.

### Metal Determination

The concentration of selected metal elements was determined in the whole soft tissues of 12 offshore mussels exposed for 48 h to the 200 nM dose of combined Cd, Cu and Hg: three tissue pools were composed (N = 4) and residual palleal water was gently drained on blotting paper just before snap freezing. Following lyophilisation, homogenization and acid digestion in Teflon containers by means of microwave oven (CEM Mars Xpress), the metal elements were determined by Atomic Absorption Spectrometry (AAS) with flame atomisation (F-AAS) for Cu and Zn, electrothermal atomisation (graphite furnace GF-AAS) for As, Cd, Cr, Pb. The dry/wet weight ratio of each tissue pool was also recorded. Analyses were performed by means of a Thermo Electron M6 mkII Atomic Absorption Spectrometer, with D_2_ and Zeeman background correction, equipped with flame burner and GF95 Graphite Furnace atomiser. The analytical settings are summarised in Table 1. For Hg determination, we employed a TDA AAS direct analyser FKV AMA254 in the following analytical conditions: wavelength 253.6 nm, accumulation time 200 sec, drying time 150 sec, decomposition time 45 sec.

**Table 1:** Analytical conditions used in the metal determination by Atomic Absorption Spectrometry (AAS).

Parametr	As	Pb	Cd	Cr	Cu	Zn
Wavelenght (nm)	193.7	283.3	228.8	357.8	324.8	213.9
Slit (nm)	0.5	0.5	0.5	0.2	0.5	0.5
Measurement time (sec)	3.0	3.0	D_2_	–	4.0	4.0
Background correction	Zeeman	D_2_	D_2_	–	D_2_	D_2_
Matrix modifier	Ni(NO_3_)_2_	NH_4_H_2_PO_4_	NH_4_H_2_PO_4_	Pd(NO_3_)_2_	–	–
Atomisation (t°C)	2600	1500	1300	2550	–	–
Flame	–	–	–	–	air/acetylene	air/acetylene
Flow (L/min)	–	–	–	–	1.0	1.1

Limits of quantification (LOQs) of the applied analytical methods are hereby reported: As 0.05 mg/kg, Pb 0.03 mg/kg; Cd 0.01 mg/kg; Cr 0.04 mg/kg; Cu 0.10 mg/kg; Zn 0.10 mg/kg; Hg 0.002 mg/kg. Quantitative data were determined by means of calibration curves of diluted specific salts and trueness of analytical data was verified by means of Certified Reference Materials (BCR185R, NRCC DORM3) analysed concurrently with samples in each analytical batch.

The metal determinations were run in triplicate and the element concentration was reported in µg/g of wet weight (ww). We used the average dry matter percentage of the three analyzed tissue pools (10.27±0.13 g/100 g) to convert the concentration values in µg/g of dry weight (dw).

### Evaluation of Micronuclei and Nuclear Abnormalities

Two independent observers, previously trained by preliminary exercises and working on coded slides, evaluated the presence of MN in epithelial-like gill cells with preserved cytoplasm in optical microscopy at 1000× magnification. MN were classified by comparison to the main nucleus (diameter <1/3 and not connected to the main nucleus, similar chromatin staining and structure). Nuclear abnormalities other than micronuclei (incomplete MN, abnormally lobed nuclei, cells with more than one nucleus, abnormal mitosis) were scored in parallel and collectively reported.

At least 2000 cells/slide, at least 2 slides/mussel and 5 mussels/dose were scored. Results are expressed per dose point in the text figures whereas individual frequencies of MN (‰) and NA (%) plus mean and standard deviation per dose point are detailed as Supplementary Information. Significant differences between treated and control mussels were evaluated from the absolute group frequencies with the G-test of goodness-of-fit adjusted for small sample size. Specifically, we tested the null hypothesis that the observed frequencies result from random sampling from a distribution with the given expected frequencies. The distribution of the *G* statistic approximately follows a *χ* ^2^ distribution with one degree of freedom.

### RNA Purification and Quality

Total RNA was purified from individual half-gill samples using the Trizol reagent (Invitrogen) in agreement to the manufacturer’s instructions. Further purification with 8 M LiCl was applied in order to eliminate glucidic contaminants. RNA samples were quantified with the UV-Vis spectrophotometer NanoDrop® ND-1000 and their quality was checked by capillary electrophoresis on Agilent 2100 Bioanalyzer with RNA 6000 Nano LabChip (Agilent Technologies). Equal amounts of RNA obtained from the five control mussels were pooled and used as reference to test individual RNAs of 3 mussels/tank in competitive hybridization to the DNA microarray slides.

### RNA Processing and Competitive Hybridization

Reference and test RNAs (10 µg) were separately incubated with a degenerated oligo-dT18 primer (10 min at 70°C) in a volume of 10 µl each, added to 20 µl of reaction mix (1x first-strand buffer, 400 U SuperScript II InvitrogenTM, 0.5 mM dATP, 0.5 mM dGTP, 0.5 mM dCTP, 0.3 mM TTP, 0.2 mM 5-(3-aminoallyl)-dUTP, 0.5 mM DTT) and reverse transcribed for 2 h at 42°C. RNA was removed from single-stranded cDNA with 3 µl of 1 N NaOH, 0.6 µl of 500 mM Na2-EDTA and the reaction mixture was then neutralized with 3 µl of 1 N HCl and 8.5 µl of 2 M HEPES. We used Microcon YM-30 (Amicon separation, MILLIPORE®) to remove buffer, unincorporated dNTPs and free amines. Finally, the cDNA samples were vacuum dried. Mono-functional NHS-esters of Cy3 or -Cy5 dyes (CyDye Post-Labeling Reactive Dye Pack, Amersham GE Healthcare) were covalently coupled to the aminoallyl-cDNA probes in DMSO for 1 h at room temperature in the dark. Then, the samples were quenched with 4.5 µl 4 M hydroxylamine for 15 min and purified with the GeneEluteTM PCR Clean-Up Kit (Sigma). Following UV-quantification, equal amounts of Cy3- or Cy5-reference and Cy5- or Cy3-test samples were combined in the same tube and ethanol-precipitated.

After resuspension in 18 µl of hybridization buffer (5× SSC, 50% formamide, 0.1% SDS) and denaturation for 3 min at 70°C, the Cy3/Cy5-coupled samples were competitively hybridised to the DNA microarray slides, covered with a 22×22 mm cover-slip and incubated overnight at 42°C in a humidified dual-slide chambers (HybChamber, GeneMachines). Before the hybridization reaction, the microarray slides were conditioned for 2 h at 42°C in a solution of 5x SSC, 100 ng/µl salmon sperm ssDNA, 5× Denhardt’s solution, 0.1% SDS.

We used the MytArray 1.0 platform, a cDNA array composed by 1712 duplicated mussel probes and unrelated controls, described in the Gene Expression Omnibus repository (see GPL1799 at http://www.ncbi.nlm.nih.gov/geo/) and printed twice on slide to allow two simultaneous array hybridizations (the reference and test samples were hybridised on the same slide in dye-swap crossed combinations). Afterwards, slides were sequentially washed with mild shaking in: 1x SSC, 0.2% SDS at 42°C for 1 min and at room temperature for 3 min; 0.1x SSC, 0.2% SDS at room temperature for 4 min; in 0.1x SSC at room temperature for 2 min. Finally, the slides were dried by air flushing.

### Microarray Data Analysis

All slides were examined with a GSI Lumonics LITE dual confocal laser scanner at 5 µm resolution. Image processing and quantification of the fluorescence signals were performed by the ScanArray Express® software (PerkinElmer) whose output files report intensity values, background, standard deviation, pixel statistics and quality parameters for each microarray probe and fluorescence detection channel (dual fluorescence detection at 570 and 670 nm for Cy3 and Cy5, respectively). Overall normalization of the fluorescence signals was performed by using the total and LOWESS (logfit) algorithm with MIDAS, the MIcroarray Data Analysis System available at The Institute of Genome Research (http://www.tigr.org/software). To identify the differentially expressed genes, log_2_ test/reference ratios of the normalised fluorescence values were computed by the permutation-based Significance Analysis of Microarrays (SAM v3.0, One-Class analysis with 200 minimal permutations) which takes into account the standard deviation of repeated measurements and, based on a modified t-test statistics assigns a q-value to each transcript probe [45]-[46]. SAM allows the selection of a delta (Δ) value providing the optimal ratio of true positives to predicted false positives, and produces a list of significantly regulated genes with a given False Discovery Rate (FDR) value. The similarity between mussel transcriptional profiles was evaluated with J-Express V2.1 by hierarchical clustering according to the Pearson correlation, complete linkage [47]. Subsequently, we traced the differentially expressed transcripts in the interactive database of *Mytilus galloprovincialis* [48] and launched new BLASTX similarity searches (10^−6^ e-value cut-off ) in the non-redundant NCBI nucleotide database with the Blast2GO tool [49]–[50]. We updated the functional annotation of the differentially expressed transcripts using multiple current resources [51]–[54], and ultimately assigned them to eleven functional categories (Metabolism and ion homeostasis, Replication, transcription and repair, Translation, Protein folding, turnover and degradation, Signal transduction, Immunity and inflammation, Cell motility and intracellular trafficking, Cell adhesion and extracellular matrix, Cell cycle and apoptosis, Development and reproduction, Unknown).

### Quantitative Real-Time Reverse-Transcriptase Polymerase Chain Reaction (qRT-PCR)

The expression patterns of eight selected transcripts were validated by quantitative RT-PCR analysis using an Applied Biosystems 7500 Fast Real-Time PCR System (Foster City, CA). Due to the limited RNA amounts residual from the microarray experiments, we could test samples pooled from the individual gill RNAs in equal amounts per each dose point. Preliminarily, we treated the samples with DNAse I (RNase-free DNase set/79254, Qiagen) to avoid the amplification of contaminant genomic DNA. Nine µg of RNA from each pooled sample were used to perform 3 independent cDNA syntheses using 2.5 µM random decamers (Invitrogen), 10 mM dNTPs and 200 U SuperScript II reverse transcriptase (Invitrogen) in 10 µl reaction volume. The reverse transcription proceeded for 2 h at 42°C, then 1 µl of purified and diluted first-strand cDNA was PCR-amplified according to the SYBR Green chemistry in 10 µl of the following reaction mixture: 1x SybrGreen PCR Master Mix (DyNAmo^TM^HS SybrGreen qPCR kit F-410S/L, Finnzymes), 1x Rox passive reference dye, and 0.5 µM of each specific primer.

Primer pairs were designed to selectively amplify fragments close to the 3′-end of the transcript using Primer Express® (Applied Biosystems) with the following guidelines: product size 100-200 bp, melting temperatures T_m_ 60±1°C, and G/C <50%. Each primer pair was preliminarily subjected to PCR to ensure the presence of a single primary amplicon, as evidenced by 1.5% agarose gel electrophoresis (data no shown). Actually, the dissociation curves of the qPCR products for all eight transcripts showed single peaks. Table 2 reports the gene annotation and identification code, forward and reverse primers and related amplicon length for each transcript sequence.

**Table 2:** Identity codes and primer details for the qRT-PCR amplification of selected mussel gene transcripts.

MytArray 1.0 ID	Mytibase ID	GenBank ID	Transcript	Forward primer (5′– 3′)	Reverse primer (5′– 3′)	Amplicon
						size (bp)
Myt01-016C08	MGC04485	AJ625847	MT10	AGGTTGTCGCTGTGGTGAATG	CAGGAACATCCAGGTGCACAT	129
*	MGC00212	AY566247	MT20	AATGGCTGGACCTTGTAACTGC	CTTTACATCCGGAACATCCGC	128
Myt01-013C12	MGC02733	AJ625244	small HSP24.1	TGTTGACAGCCCAAGGAGAAC	AACATCTTCAGCCATTGGTGC	167
Myt01-007D04	MGC00749	AJ624024	SQSTM1	CCTTCTGTGCCAAGTACAACCA	TCAGAAGAGAGGTCAACCAGCC	121
Myt01-010B03	MGC02267	AJ624615	HSC70	GCAGCCTTGTCTAGTTTGGCA	GCATCACAAGAGCCAGGTTTG	103
Myt01-016G09	MGC00670	AJ625915	HSP90	TCATGGAGGCTCTTCAAGCTG	CAGCTGCCGATTCCCAGATAT	147
Myt01-013D11	MGC00220	AJ625268	ferritin	ATGCAGGTTGCTTTGTCCTTG	GGCGTTCACTTGTTCTTCCAA	135
Myt01-006C05	MGC01801	AJ623737	GADD45 gamma	TCTGTTTCGGCCATCTCTGGT	GCACAGGAAGACGGCAGAATT	110

Abbreviations: *gadd,* growth arrest and DNA damage inducible; *hsp/hsc,* heat shock protein/cognate;

*mt*, metallothionein; *sqstm*, sequestosome.

We performed the PCR reactions using the same Universal 18S rRNA (QuantumRNA™ 18S Internal Standards, Ambion) as internal reference and thermal cycling protocol previously reported [32] and calculated the relative gene expression values with the ΔΔCt method [55]. In agreement to other authors, we found the expression levels of the ribosomal gene 18S essentially stable [56]. To ensure that the abundant 18S transcripts do not overwhelm other transcripts during the amplification reaction, we adopted the empirical and optimal 1∶9 ratio of 18S primers:18S competimers. All samples and the internal reference were run and amplified in triplicate. Prior to the relative quantitative analysis, a standard curve was produced by using four serial dilutions of the mixed RT products from all the dose points and the corresponding efficiencies for each primer pair were calculated according to the equation E = 10^(−1/slope)^ (correlation coefficients were >0.98).

Hence, we measured the change in expression of the eight selected transcripts relative to the reference 18S rRNA in gill samples from mussels exposed to the 50, 100 and 200 nM metal mixtures in comparison to control mussels.

## Results

### Mussel Exposure to Nanomolar Cd, Cu, Hg Mixtures and Metal Analysis

We used offshore mussels collected in late Spring to test the effects of the same metal mixture previously chosen to investigate the transcriptional response of the Mediterranean mussels to co-occurring metal contaminants [32]. Compared to the earlier work, we confirmed the exposure time (48 h) and extended the dose range of combined Cd, Cu and Hg chloride salts from 50 to 200 nM final concentrations, dose levels which did not induce mortality nor visible behavioural changes in the treated mussels.

The triplicate analysis of selected metal elements performed by atomic spectrometry on the pooled flesh of mussels exposed to the 200 nM metal dose for 48 h indicated mean concentration values of 1.42 (Pb), 1.56 (Cd), 0.24 (Cr), 2.85 (As), 3.08 (Cu), 31.38 (Zn) and 22.52 (Hg) µg/g ww (Table 3). Hence, in the time of two days the treated mussels showed 69, 242 and 561 times higher amounts than the nominal delivered doses of Cd, Cu and Hg, respectively. In Table 3, the metal concentration values are also reported in µg/g of dw for comparative purposes.

**Table 3:** Concentration of selected elements in the mussels exposed for 48 h to the 200 nM metal dose.

Element	Dose unit	Mean	St. Dev	BCF
Pb		1.48	0.09	
		1.36	0.15	
		1.43	0.08	
	µg/g ww	**1.42**	**0.04**	
	µg/g dw	13.86	0.36	
**Cd**		1.63	0.03	**69.3 x**
		1.48	0.13	
		1.57	0.11	
	µg/g ww	**1.56**	**0.06**	
	µg/g dw	15.16	0.54	
Cr		0.26	0.03	
		0.22	0.03	
		0.24	0.02	
	µg/g ww	**0.24**	**0.01**	
	µg/g dw	2.34	0.09	
As		2.99	0.22	
		2.82	0.09	
		2.74	0.18	
	µg/g ww	**2.85**	**0.07**	
	µg/g dw	27.74	0.68	
**Cu**		3.84	1.13	**242.0 x**
		2.62	0.04	
		2.76	0.03	
	µg/g ww	**3.08**	**0.63**	
	µg/g dw	29.94	6.18	
Zn		33.49	2.49	
		29.47	1.17	
		31.20	0.55	
	µg/g ww	**31.38**	**0.99**	
	µg/g dw	305.50	9.63	
**Hg**		23.22	0.91	**561.4 x**
		21.81	1.70	
		22.54	0.94	
	µg/g ww	**22.52**	**0.45**	
	µg/g dw	219.24	4.37	

Triplicate analysis of three tissue pools, each one composed by the whole flesh of four mussels. Mean and standard deviation are reported in ww and dw; BCF, bioconcentration factor.

### Frequency of Micronuclei and Nuclear Abnormalities in the Mussel Gill Cells

**Figure 1:**
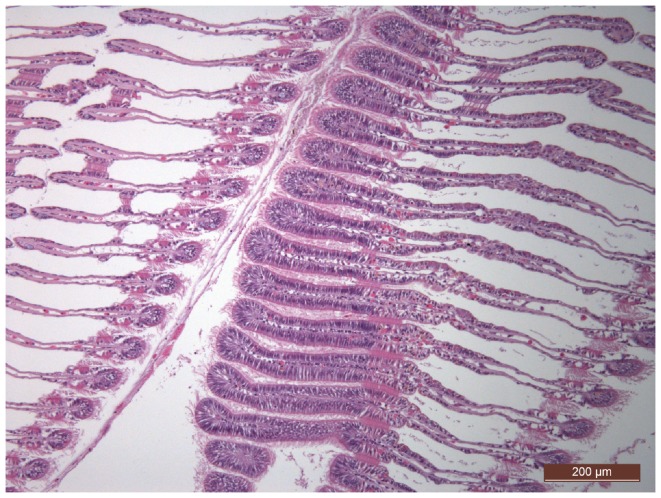
The mussel gill structure. In the mussel shell, rows of interconnected gill filaments folded to form a W-shape create the trabecular structure of the gill lamellae on either sides of the visceral mass, with apical ciliated cells facilitating the flux of water, oxygen uptake and food collection (3 µm sections observed after haematoxylin and eosin staining in bright field microscopy, by courtesy of Tobia Pretto, Reference Natl. Centre on mollusc diseases, IZSVe, Italy).

Figure 1 illustrates the gill organization in the filibranch bivalve *M. galloprovincialis*: many ordered and interconnected filaments suggest the sea water circulation in the palleal cavity and efficient filter feeding activity. We evaluated the genotoxic effects of Cd, Cu and Hg combined at 50–200 nM equimolar doses in the main epithelial-like gill cells, gently recovered and processed from single offshore mussels after the 48 h exposure. For comparison, the mussel treatment and individual cytogenetic analysis was repeated with marketed winter mussels of Spanish origin.

**Figure 2:**
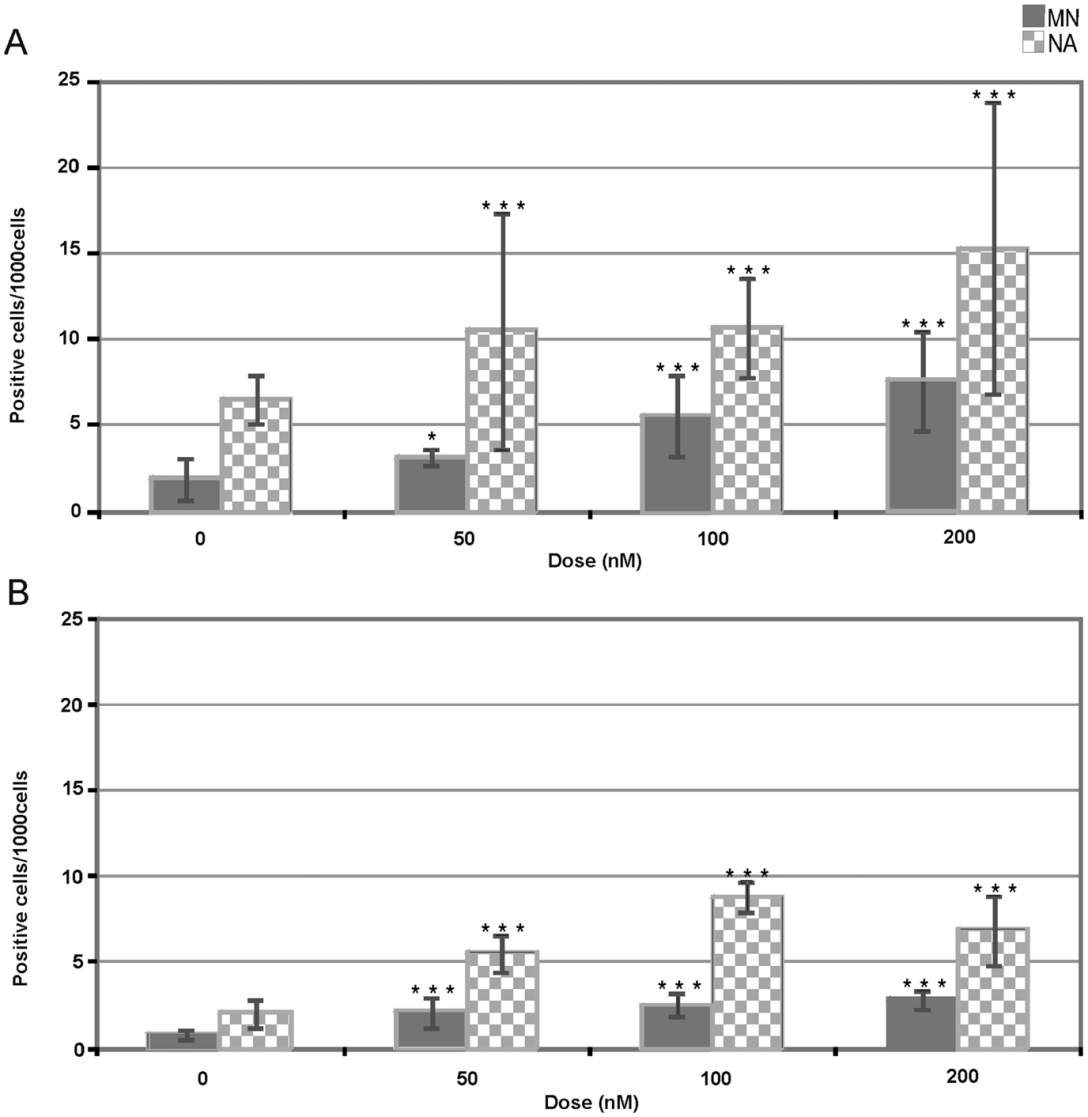
Occurrence of gill cells with micronuclei (MN) or nuclear abnormalities (NA) in the mussels exposed for 48 h to the combined metals. Panels A and B refer to offshore mussels collected in late spring and marketed winter mussels, respectively. Mean (N = 5) and standard deviation per dose point are reported (* and *** indicate significant increases at p<0.05 and 0.001 compared to the control mussels).

The frequency values of epithelial-like gill cells with MN or NA detected in both the native and imported mussels exposed to the 50, 100 and 200 nM metal doses are reported in Figure 2A and 2B, respectively. On average, the micronucleated cells raised from 1.90 to 3.18, 5.56, 7.53 ‰ in late spring offshore mussels and from 0.81 to 2.08, 2.52, 2.85 ‰ in the marketed winter mussels (detailed results are available in the Table S1). Significant increases of MN and NA frequencies were observed in both mussel groups already at the 50 nM exposure dose. In other words, significant genotoxic effects were detected at the lowest tested dose irrespective to the functional mussel condition, with more pronounced dose-response effects in the native mussels of late spring than in the winter mussels.

The presence of cells with morphologically altered nucleus (i.e. incomplete MN, lobed or multiple nuclei, nuclei connected by chromatin bridges in the same cell) was evaluated on the same slides in parallel. The mean frequency of gill cells with NA confirmed the genotoxicity of the metal mixture as such cells increased from control values of 6.55 up to 15.32 ‰ (200 nM dose) in the offshore mussels and from control values of 2.08 to 8.81 (100 nM dose) then decreased to 6.86 ‰ in the winter mussels (see Table S1). Following 2 and 6 extra days of depuration, the micronucleated gill cells diminished to 4.24±0.99 and 4.71±2.51 ‰, respectively, without reaching the original background levels in offshore mussels treated with the 200 nM metal dose.

### Gill Gene Expression Profiles in Mussels Exposed to the Metal Mixtures

In order to evaluate the gene expression changes induced in the mussel gills by the metal mixture we used MytArray 1.0 (GPL1799), a cDNA array gathering 1758 probes (1712 from *M. galloprovincialis*) spotted in duplicate and designed at the 3′end of transcripts expressed in multiple mussel tissues. Double competitive hybridization in dye swap labelling of the individual gill RNAs yielded four fluorescent signals per probe (3 samples/dose point, 18 hybridization experiments). Following normalization, the replicated values of each array probe were averaged. The hierarchical clustering of the resulting fluorescence data obtained from individual mussel gills indicated the full consistency of the microarray experiments since the arrays A and B from the same dye-swap hybridization experiment always clustered together (Figure S1).

**Figure 3:**
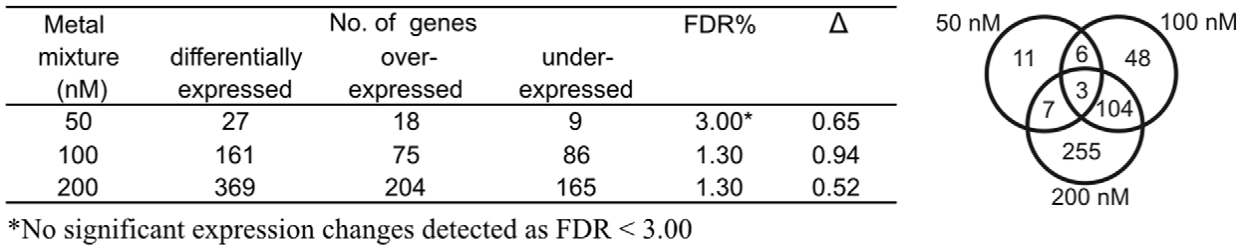
Number of genes differentially expressed in the mussel gills per metal dose in regard to control group. Left: over- and under-expressed genes with related false discovery rate (FDR) and delta value (Δ). Right: Venn diagram of common and unique genes modulated at 50, 100 and 200 nM metal mixtures (right).

Figure 3 (left) illustrates the total number of transcriptional changes detected in the gills of mussels treated with the 50, 100 and 200 nM metal doses. The complete list of differentially expressed genes with related identity codes, best sequence similarity, assigned functional category and relative expression values detected at the three tested doses, is available in the Tables S2, S3 and S4. Only 27 mussel probes (1.6%) with 0.03 FDR value were significantly modulated at the 50 nM dose whereas 161 (9.4%) and 369 (21.6%) mussel probes with 0.01 FDR marked the effect of the metal mixture at the 100 and 200 nM doses. The Venn diagram resulting from these data revealed 3 and 107 transcript probes in common between the three dose groups and the two highest doses, respectively (Figure 3, right).

The differentially expressed genes in common to one or more treatment doses have been extracted in the Table S5 for the benefit of reading. Three transcripts denoting heat shock protein HSP90, small HSP24.1 and alpha tubulin were significantly modulated at all treatment doses. Additional 6 transcripts similar to translation initiation factor 5A, precollagen P, a C1q domain containing protein plus other unknown sequences were significantly modulated at the 50 and 100 nM doses. Among the transcripts (additional 104) differentially expressed at the 100 nM and 200 nM doses, we found many sequences related to oxidative stress defence and metal homeostasis/detoxification such as those for MT10B, various chaperones (HSP90, HSP70, chaperonin subunit 7, small HSP24.1), components of the cell machinery for protein degradation and turnover (sequestosome SQSTM1, FK506 binding protein), membrane transporters and receptors (magnesium and taurin transporters), and glutathione S-transferase (GST) enzymes (GSTpi1, microsomal GST3).

At the lowest exposure dose (50 nM) the magnitude of the transcriptional changes ranged between ±0.67 value (median log_2_ test/reference ratio from three individual gill samples, equivalent to ±1.6 fold change), with the most over-expressed transcripts referred to an unknown sequence, HSP90, small HSP24.1 and translation initiation factor 5A. The 100 nM and 200 nM metal doses induced wider functional adjustments since the median values of differential expression ranged from −1.69 to 3.20 (up to 9.2 fold change) and from −1.58 to 4.28 (up to 19.5 fold change), respectively. The transcripts most over-expressed at the 100 nM dose were recognised as for the ubiquitin-binding protein SQSTM1, immunophilin FK506-binding protein, GST, small HSP24.1, and the unknown Myt01-014C10. The above mentioned cases (SQSTM1, GST, the PPIase and various HSPs) plus transcripts for MT10B, an apoptosis inhibitor (Myt01-017B11), an incilarin-like C-type lectin, a suppressor of cytokine signalling, a C1q domain containing and several unknown sequences were the most over-expressed at the 200 nM dose. Also transcripts related to the genotoxic stress (i.e. growth arrest and DNA damage inducible protein GADD 45 gamma), essential to cell replication (BAT2, nucleolar protein p130) or likely involved in apoptotic pathways (caspases 3 and 7, defender against apoptotic cell death 1, translationally controlled tumor protein TCTP) were over-expressed at the highest dose. Down-regulation of transcripts denoting INO80 (component of chromatin remodelling complexes involved in the repair of DNA strand breaks), a distinct apoptosis inhibitor (Myt01-007H08) and unknown sequences such as Myt01-008B03 and Myt01-016D12 was evident at the highest treatment doses. Overall, these data are consistent with the increased frequency of cells with MN and NA observed in the same mussel gill samples.

**Figure 4:**
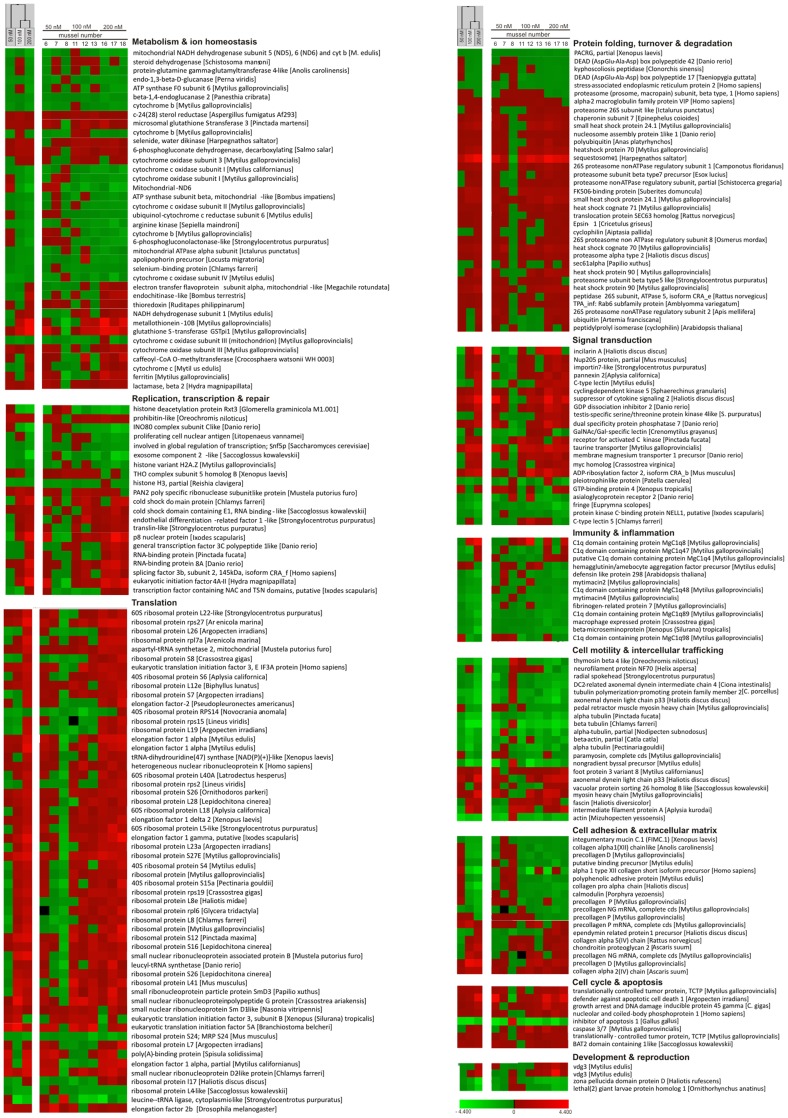
Unsupervised hierarchical clustering and relative expression trends of annotated genes differentially expressed in the mussel gills at least in one treatment dose. Clustering of median values per dose point (left), individual trends (middle) and transcript description (right) have been reorganized by functional category.

The hierarchical clustering (Pearson correlation, complete linkage) of the expression data averaged per dose point (i.e. 3 individual mussel gills) greatly supported the interpretation of the transcriptional trends per functional gene category whereas the interindividual variability of the metal-induced modulation of gene expression can be appreciated in the Tables S2, S3, S4 (the differentially expressed genes modulated in more than one treatment dose were extracted in the Table S5). As depicted in Figure 4 (upper dendogram and related heat map), the transcriptional responses to the 100 and 200 nM doses are more similar to each other than to the 50 nM metal dose. The Principal Component Analysis performed on the median expression values per dose point revealed two main factors explaining together 92.1% of the total variance, with the factor 1 explaining 74.35% variance and including many over-expressed probes (data not shown). In Figure 4, the transcript list of the genes differentially expressed at least at one dose point, with the associated individual trends and transcript descriptions has been reorganized by functional category, according to sequence similarity and protein domain searches, GO terms and KEGG pathways (see also Tables S2, S3, S4, S5). Whilst assigning a single transcript to one functional category to simplify the elucidation of the transcriptional profiles, we are not forgetting that eukaryotic proteins are often featured by multiple functional domains and roles.

**Figure 5:**
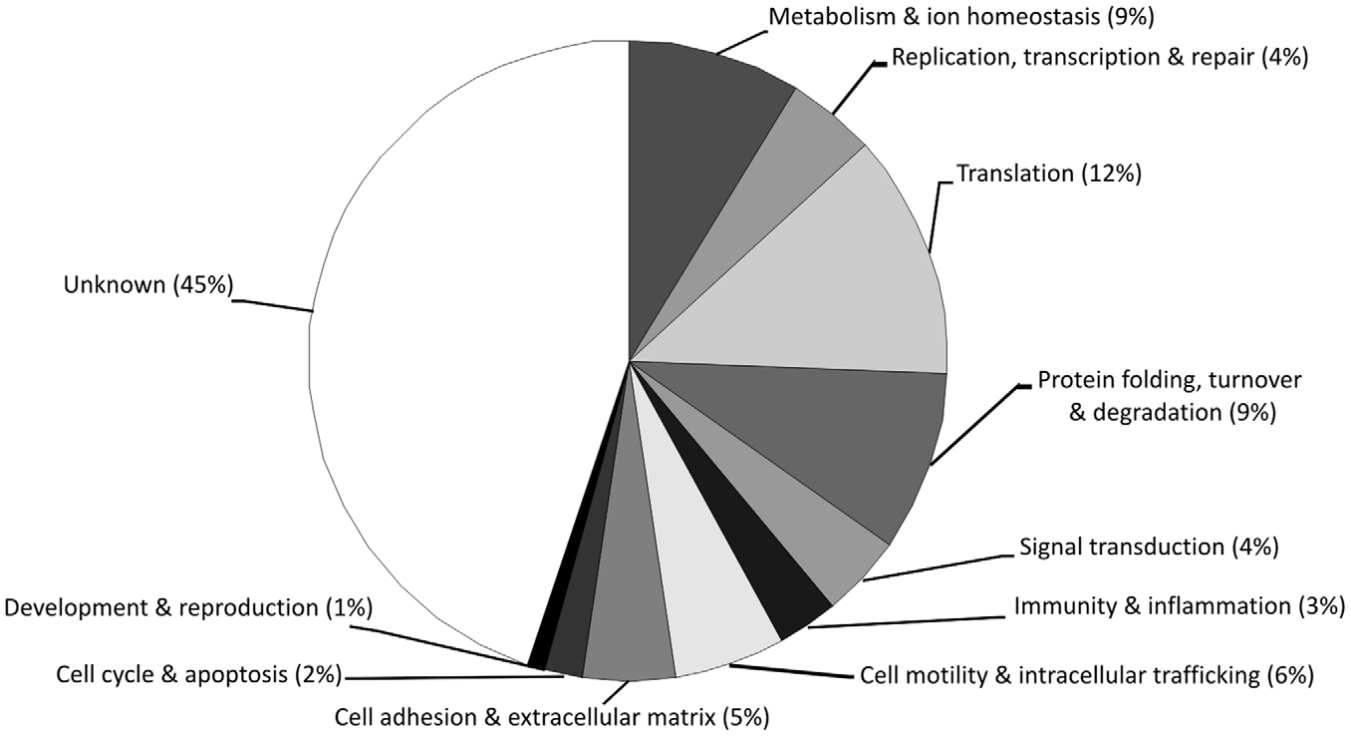
Distribution of all differentially expressed genes in different functional categories.

The annotation process revealed the overall complexity of the gill cell response (Figure 5). Actually, the metal mixture modulated the expression of many ribosomal proteins and translation factors including EL1 and EL2 (Translation 12%), ubiquitin and proteasome related proteins (Protein folding, turnover and degradation 9%), mitochondrial respiratory proteins and various enzymes (Metabolism and ion homeostasis 9%), cytoskeleton and collagen components (Cell motility and intracellular trafficking 6%; Cell adhesion and extracellular matrix 5%). Relevant transcriptional changes were also evident in other functional gene categories, and in the large fraction of transcripts having still unknown function (Unknown, 45%) also reported separately in the Figure S2.

### Validation of Microarray Data by qRT-PCR

**Figure 6:**
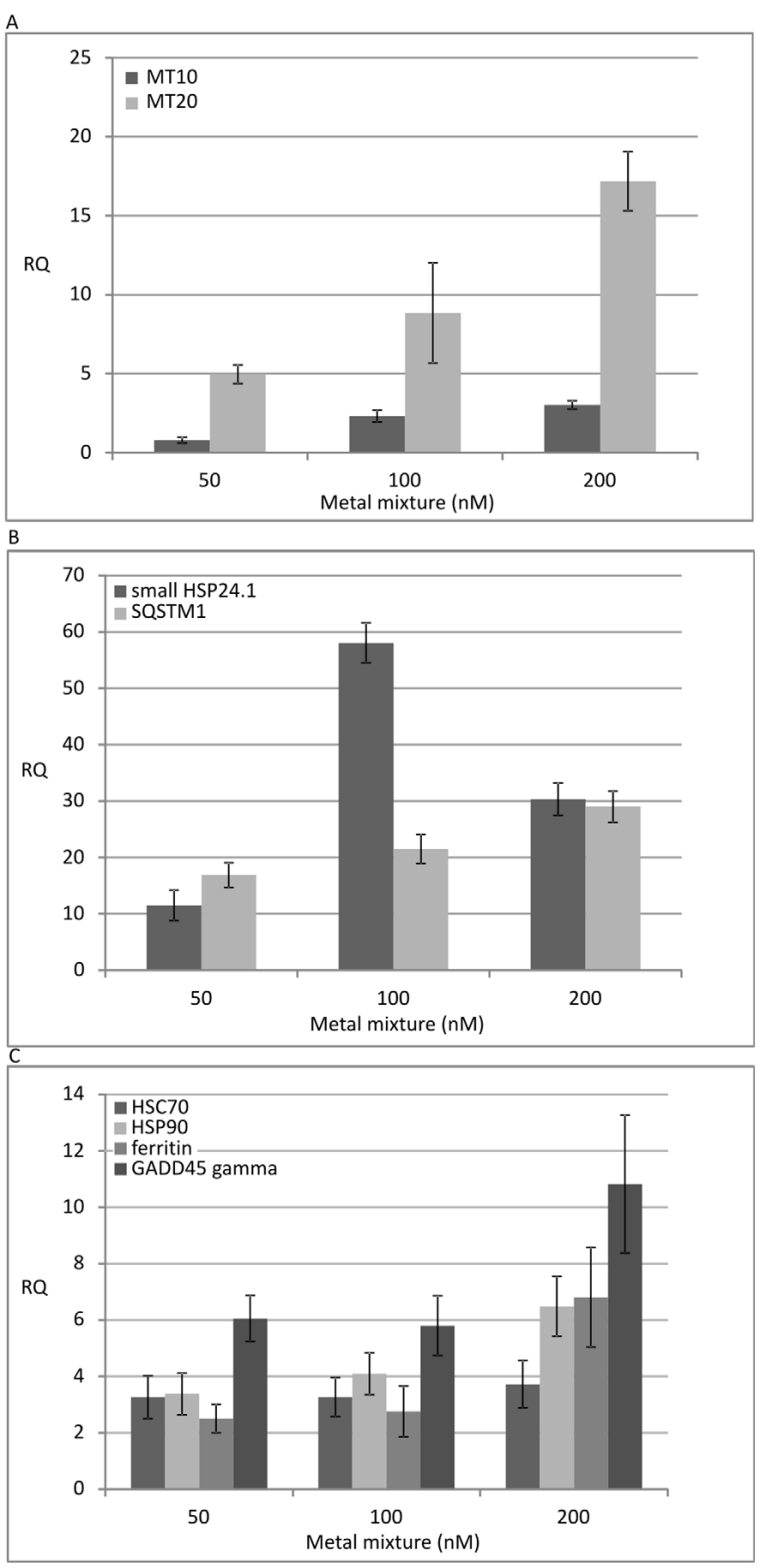
Relative quantification levels (RQ) of selected mussel transcripts significantly modulated after exposure to the combined metals. Pooled gill RNAs from both treated and control mussels were tested in triplicate according to the 2−ΔΔCt method and considering 18S rRNA as endogenous control gene.

Quantitative RT-PCR analysis was performed to validate the expression patterns of the significantly modulated transcripts for MT10B, ferritin, GADD45 gamma, small HSP24.1, HSC70, HSP90, SQSTM1 and MT20IV, the latter involved in the mussel response to heavy metals but not present in the MytArray 1.0. Overall, we confirmed the expression patterns detected on DNA microarray since the quantitative expression values of the selected transcripts increased with the metal dose more or less markedly in all cases (Figure 6 A–C, Table S6). On average, the relative expression of MT20 transcripts increased up to 17.2 RQ (relative quantification value) at the 200 nM metal dose, nearly 5.7 times more than those of MT10, while the transcripts for small HSP24.1 and SQSTM1 showed the highest expression levels (58.0 and 29.0 RQ at the 100 nM dose, respectively) and those for HSP70, HSP90, ferritin and GADD45 were in the range of 3.7–10.8 RQ.

### Modelling the Transcriptional Response of the Mussel Gill Cells to the Metal Mixture

**Figure 7:**
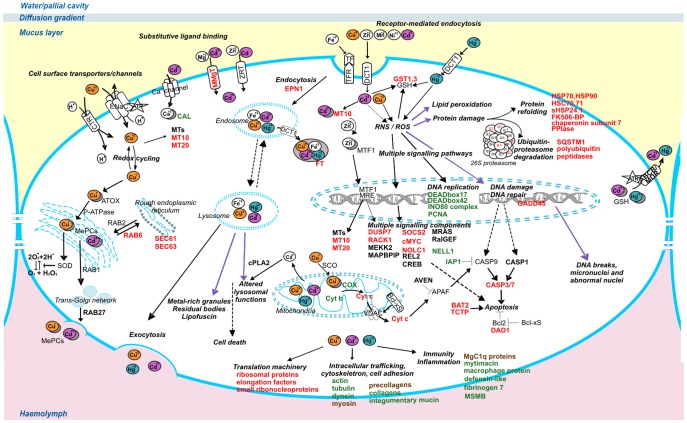
Representation of the gill cell response at 48 hours from the mussel exposure to the combined metals. Cd, Cu and Hg ions can enter the cytosol across various transporters or channels, also by endocytosis, with potential inhibition of physiological membrane import processes. Contaminant metal ions can bind small molecules (e.g. GSH), transporters and chaperons (e.g. ferritin, MTs), and apoproteins (e.g. superoxide dismutase, cytochrome c oxidase). Hence, they may compete with endogenous cations, substitute their natural ligands, disturb Ca^++^ homeostasis, and accumulate also in metal-rich or mineralized granules. Redox reactions with sulfur groups and direct/indirect formation of ROS/RNS can trigger multiple signalling pathways, disrupt the regulated expression of many genes and deplete the antioxidant cell defences. The unbalance towards the oxidative stress associated to extensive damage to organelles such as mytochondria and lysosomes, macromolecules and their precursors may lead to cell death either by necrosis or apoptosis. Deregulation of genes involved in the cell cycle homeostasis might also lead to uncontrolled replication and tumour development. Based on the experimental data, we have exemplified genes up-regulated (red), down-regulated (green) and contrasting (brown) expression trends, altogether outlining the enhancement or depression of specific cellular processes. Transcripts represented in the MytArray are reported in bold. Related abbreviations are the following. α: 26S proteasome subunit α (α1: Myt01-003G03; α2: Myt01-007D11, Myt01-013D06). ABCB: ATP binding cassette p-glycoprotein (Myt01-004E12, Myt01-018G06). APAF: Apoptotic Peptidase Activating Factor. ATP: Adenosine-5'-TriPhosphate. ATOX: AnTiOXdant protein AVEN: Caspase Activation Inhibitor (Myt01-018H01). β: proteasome subunit beta type (β1: Myt01-002G08; β5: Myt01-007H02; β7: Myt01-016F07). BAT2: HLA-B-associated Transcript 2 (BAT2 domain containing 1-like: Myt01-012B10). Bcl-xS: B cell lymphoma X apoptosis regulator. Bcl2: B cell lymphoma 2-like protein 1. CAL: Calmodulin (Myt01-003H01). CASP: Caspase (CASP3/7: Myt01-011F10; CASP1: Myt01-014F12). cMyc: cellular Myelocytomatosis proto-oncogene (Myt01-003C05). COX: Cytochrome c Oxidase (subunit I: Myt01-006G10, Myt01-019B06; subunit II: Myt01-019B11; subunit III: Myt01-004F10, Myt01-019B03, Myt01-016F03; subunit IV: Myt01-007E11). CREB: C-amp-Responsive Element-Binding (Myt01-009E09). cPLA2: cytoplasmic Ca2+-dependent PhosphoLipase A2 (Myt01-014H05). CTR: Cell surface Transporter. Cytb: Cytochrome b (Myt01-019B12, Myt01-011C05). Cytc: Cytochrome c (Myt01-005A03). DAD1: Defender Against apoptotic cell Death 1 (Myt01-017B11). DCT1: Divalent Cation Transporter 1. DEAD box: DEAD (Asp-Glu-Ala-Asp) box polypeptide 17, Myt01-007F09; polypeptide 42 (Myt01-017E11). DUSP7: Dual Specificity protein Phosphatase 7 (Myt01-016G07). ENaC; Epithelial Na Channel. EPN1: Epsin1 (Myt01-014H03). Fk506-BP: FK506-binding protein (Myt01-012A04). FT: Ferritin (Myt01-013D11). GSH: Glutathione. GST: Glutathione S-Transferase (GST3: Myt01-012G04; GSTpi1: Myt01-010C12). IAP1: Inhibitor of APoptosis 1 (Myt01-007H08). INO80: INO80 complex subunit C-like (Myt01-003G12). MAPBPIP: mitogen-activated protein-binding protein-interacting protein (Myt01-015D08). MEKK5: Mitogen activated protein Kinase Kinase Kinase 5 (Myt01-006H07). MePCs: Metal Protein Complexes. MgC1q: *M galloprovincialis* C1q domain containing protein (MgC1q8: Myt01-015F11; MgC1q4: Myt01-015C12; MgC1q48: Myt01-018E07; MgC1q89: Myt01-015H10). MMgT: Membrane Magnesium Transporter (Myt01-002C12). MRAS: Ras-related protein M-Ras (Myt01-005E12). MRE: Metal Response Element. MRP: Multidrug Resistance-associated Protein (Myt01-010D05). MSMB: MicroSeMinoprotein Beta (Myt01-016C09). MT: Metallothionein (MT10: Myt01-016C08). MTF: MRE-binding Transcription Factor. NELL1: protein kinase C-binding protein, Neural Epidermal growth factor-Like 1 (Myt01-015F09). NOLC1: Nucleolar and Coiled-body phosphoprotein 1 (Myt01-015B10). P-ATPase: P-type ATPase. PCNA: Proliferating Cell Nuclear Antigen (Myt01-016A01). PPIase: PeptidylProlyl Isomerase (cyclophilin-like) (Myt01-009D06). RAB: Ras-related GTP-binding protein (Rab6 subfamily protein: Myt01-002B09; RAB27: Myt01-018A11). RACK: Receptor for Activated C-Kinase (Myt01-007H10). RalGEF: Ral Guanine nucleotide Exchange Factor (Myt01-009C10). RNS : Reactive Nitrogen Species. ROS: Reactive Oxygen Species. SEC: *S. cerevisiae* endoplasmic reticulum membrane protein translocator (SEC61: Myt01-011C12; SEC63: Myt01-018G07). SCO: Synthesis of Cytochrome c Oxidase SOCS2: Suppressor Of Cytokine Signaling 2 (Myt01-012D01). SOD: SuperOxide Dismutase. TCTP: Translationally Controlled Tumor Protein (Myt01-007H05, Myt01-010H05). TF: TransFerrin. TFR: TransFerrin Receptor. VDAC: mitochondrial Voltage-Dependent Anion membrane Channel. ZRT: Zinc-Regulated Transporter.

One main advantage of the DNA microarray analysis is to provide an integrated view of the functional cell response to a given stimulus. According to the available knowledge, we used the lists of the differentially expressed genes to prepare a schematic, though not exhaustive, view of the molecules, organelles and pathways involved in the acute mussel gill response to equimolar mixtures of Cd, Cu and Hg, delivered as soluble metal salts. In Figure 7, we depicted a gill cell as functional element between the external environment and haemolymph. Influx, trafficking and extrusion of Cd, Cu and Hg metal ions is mediated by transporters and channel proteins, metal binding peptides and chaperones, apoproteins and metal insertases as well as endocytic/exocytic mechanisms. When escaping their proper ligands and regulatory mechanisms, free metal ions can negatively influence the cell functioning at different compartments, from the external membrane to the endo-lysosomal system and mitochondria, and in the cell nucleus. The acronyms of specific molecules are in bold when putatively/certainly identified by a cDNA probe of MytArray 1.0. Transcripts up-regulated or down-regulated in the gills of metal-treated mussels are reported in red and green, respectively (in brown those showing contrasting trends at the tested metal doses). For more details see the Abbreviation list below in the text (Section 7) and Tables S2, S3, S4, S5.

## Discussion

### Mussels Significantly Accumulate Metals Present in the Surrounding Water Phase

According to the NOAA Mussel Watch Program, conducted in the Unites States in the years 1986-2005, the highest concentrations for both metal and organic contaminants are typically found near to urban and industrial areas of estuarine and coastal systems. Many of the chemical contaminants included in such wide investigation are indicated as Priority Pollutants by the US Environmental Protection Agency: most of them are toxic to aquatic organisms, some of them having the potential to be taken up, magnified along the food chain and transferred to humans [16]. Two decades of NOAA Mussel Watch data allowed the classification of low, medium and high concentration ranges of trace metals in *Mytilus* species: in particular, the concentration ranges of cadmium, copper and mercury were 0–3/4–9/10–20 (Cd), 5–16/17–39/40–857 (Cu) and 0.00–0.17/0.18–0.35/0.36–1.28 (Hg) ppm dw, respectively. The same study made also evident differences of bioaccumulation between coastal bivalves since for instance oysters relative to mussels showed 10 times higher copper concentrations [16].

The metal mixture selected in this study is just one of the potential cocktails occurring in coastal waters. Purposely, we expanded the dose range of the same metal mixture (50–200 nM chloride salts of each Cd, Cu and Hg) to extend previous transcriptional data and compare the transcription profiles of digestive gland and gills, and to anchor the multiple gene expression changes to more stable indicators of structural damage such as the occurrence of MN and other NA. The selected dose range of metal salts is equivalent to 5.6–22.5, 3.2–12.7 and 10.0–40.1 µg/L of Cd, Cu and Hg, respectively. The exposure time of 48 h reproduces an acute exposure, useful to recognize the early changes induced by the metal mixture, in the general aim to dissect the complexity and inconstancy of contaminant levels in the natural environment into more simple questions. Long-lasting exposure to toxic metals may be associated to more complex biokinetics also because of adaptive organism responses [30].

The metal amounts determined by atomic absorption spectrometry in the mussel tissues suggested significant metal uptake from the contaminated sea water, with bioconcentration factors (BCFs) estimated to increase in the following order: Cd<Cu<Hg. Excluding Hg, the metal body burdens measured in this study are consistent with concentration ranges reported elsewhere, for instance those concerning mussels from the Galitian and Cantabrian coasts in Spain [57]. Body burdens and empirical BCFs suggest more efficient absorption of the essential metal Cu compared to the non-regulated Cd (29.9 vs. 15.2 µg/g dw) whereas the visibly high concentration of Hg (219.2 µg/g dw) likely reflects the high absolute mass of Hg delivered to mussels in the time of two days and, possibly, the overwhelming of sequestration/extrusion mechanisms. Greater BCFs reported elsewhere for Cd and Hg (761–3000 and 1000 L/Kg ww, respectively, with the latter referred to sediment-bound Hg bioavailability [37]. Since hundreds of ppm of Hg have been recently reported in lagoon sludges, especially close to industrial sites, significant bioconcentration of Hg in autochthonous bivalve species is a factual hypothesis [41].

In mussels exposed to Ag, Cu, Hg and Pb for 94 days, the bioconcentration of Hg was particularly evident in the abfrontal gill filament region [26] and significant increase of both Cu and Hg levels were detected in the mussel gills after some days of co-exposure of *M. galloprovincialis* to 0.08 mg/L of Cu and 0.08 mg/L Hg (126 and 40 nM, respectively) [58]. In the latter study, pronounced metal accumulation was observed in the frontal part of the gill filaments and, specifically, Hg significantly increased in the gills of mussels co-exposed to Cu and Hg for 11 days during additional 4 and 7 days of depuration. For the benefit of information, Hg has an higher covalent index than Cd and Cu (4.08 vs. 2.71 and 2.64, respectively), being the covalent index a parameter reflecting the tendency of metals to bind ligands with S-donor atoms [37].

### Significant Levels of Gill DNA Damage Appear after 48 h Exposure to the Metal Mixture

The gill tissue is mostly composed by ciliated and not ciliated epithelial cells, and active cell proliferation has been demonstrated throughout the gill filaments [25]. Epithelial-like gill cells are sensitive to micronucleus formation and the frequency of cells with MN or with other NA, a complementary parameter of chromosomal damage, is widely used to measure genotoxic effects in marine mussels [43], [59]. The frequency of both MN and NA significantly increased in the mussels exposed to 50–200 nM doses of combined metals, and reached values comparable to those detected in metal polluted sites along the Spanish Mediterranean coast [60] and higher than those registered in the industrial area of the Venice lagoon [43]–[44].

In a previous study, the exposure of mussels for 5 days to the same metals indicated Hg>Cu>Cd in decreasing order of genotoxic potency when tested as single compounds at the maximum doses of 184, 80 and 32 µg/L as Cd, Cu and Hg chlorides, respectively. In detail, the mussel exposure to these metals combined at 18.4, 10 and 32 µg as chlorides, respectively, caused a significant increase of micronucleated gill cells (6.73±2.3 ‰ vs. 2.8±1.4 ‰ control values) [31]. Such equimolar (∼100 nM) metal mixture also induced significant MT increase and decreased stability of the lysosomal membranes in the mussel digestive gland.

In our case, the overall higher frequency of MN and NA recorded in the offshore (late spring) mussels related to the marketed (winter) mussels with ripe gametes or depleted gonads suggests better functional state and higher cell replication rates in the former group. Significant chromosomal damage was evident already at the 50 nM dose, irrespective of the season and mussel condition, hence, the measure of MN and NA appears to be a robust biomarker of exposure, scarcely biased by the fluctuations of the external and internal environment under the applied experimental conditions. The diverse amounts of granulocytes observed in gill cell preparations of the treated vs. control mussels (data not shown) suggests hemocytic infiltration and modulation of the haemocyte functions in response to the metal cocktail, in agreement with previous hypotheses [25], [44].

### Increasingly Complex Transcriptional Changes Occur in the Gills of the Metal-treated Mussels

To detect the gene expression changes induced by the combined metals in the mussel gills, we used a previously defined cDNA microarray [32] whose reliability is supported by a number of recently reviewed studies [61]–[62]. The transcriptional profiles obtained from three mussels per dose point revealed a dose-related increase of significantly modulated probes: 161 and 369 transcriptional changes characterized the mussels treated with 100 and 200 nM metal dose whereas the 27 significant changes detected at the 50 nM dose are affected by 3% probability of false positives. Also the magnitude of the observed changes increased with the metal dose (the genes up-regulated at 50, 100 and 200 nM metal doses reached an expression fold change of 1.6, 9.2 and 19.5, respectively). According to the consistent fraction of over-expressed probes, the applied metal doses did not massively reduce the cell functioning; moreover, the 104 transcripts probes modulated at both 100 and 200 nM doses suggested common functional adjustments in response to such metal concentrations. The hierarchical clustering of the normalized fluorescence data confirmed the similarity of the gill cell response to the 100 and 200 nM doses. As in the case of the chromosomal damage (individual frequencies of gill cells with MN and NA), the DNA microarray analysis highlighted consistent, though reasonable, levels of inter-individual variability of the gene expression levels.

The gill gene expression profiles presented in this study appear more informative than those obtained with MytArray 1.0 from the digestive gland of mussels treated with the same metal mixture [32]. Updated functional annotation of the transcripts represented in the DNA microarray platform, greater number of differentially expressed genes (161 vs. 104 detected in gills vs. digestive gland at the 100 nM dose) and expanded metal dose range are the likely explanations. The comparison also confirms the mussel gills as target of toxic metals, hence tissue of priority for measuring exposure biomarkers.

The functional annotation and re-organization of the differentially expressed transcripts by gene category made evident a global modulation of the cellular processes, with many still unknown transcripts also recruited in response to the metal mixture. The resulting gene expression signatures relate the composition of the MytArray platform (1712 cDNA probes which cover the basic functions of the main mussel tissues) with dose-related expression changes exemplified by SQSTM1 (Myt01-007D04; 2, 10 and 21-fold up-regulation) and apoptosis inhibitor 1 (Myt01-007H08; 1.7, 2.9- and 2.4 fold down-regulation).

In agreement with current concepts on the metal toxicity, the gene categories most abundantly differentially expressed in the gills of the treated mussels were Translation, Protein folding-turnover-degradation and Metabolism-ion homeostasis. The complex modulation of genes involved in the metabolism of carbohydrates, aminoacids and lipoproteins, and in the mitochondrial respiration, suggest enhanced turnover of many gill cell components. Accordingly, global protein synthesis alteration and accumulation of non-translated mRNAs have been reported in the digestive gland of mussels exposed to Cd for 15 days, with initial reduction and subsequent significant increases of the polysome levels [63].

Several transcripts for ribosome proteins, heat shock proteins (HSP90, HSP70, small HSP24.1 and chaperonin subunit 7), putative proteinases and proteinase inhibitors (e.g. alpha-2 macroglobulin), proteasome subunits, ubiquitin, were over-expressed in the treated mussels. Such evidence supports the occurrence of oxidative stress with consequent boost of protein synthesis, folding and degradation. Actually, the highest up-regulated mussel transcript at the 100 and 200 nM doses was SQSTM1, a multifunctional protein induced by oxidative damage which can bind ubiquitin and activate the NF-kB pathway in response to upstream signals. Likewise, the highest doses of the metal mixture induced a significant over-expression of a mussel transcript denoting an FK506 binding protein. Immunophilins with peptidylprolyl cis/trans isomerase –PPIase- domains are expected to regulate many biological processes such as receptor signalling and protein folding through protein–protein interactions. The over-expression of the mammalian FK506 binding protein 51 and its atypical mitochondrial localization has been reported to protect the cells against oxidative damage [64]. At the 200 nM metal dose, we also found significantly over-expressed transcripts denoting SEC 61 and SEC 63. In the endoplasmic reticulum, the Sec 61 multimeric complex allows the protein translocation across lipid bilayers, the backward transport of proteins destined to ubiquitination and proteosomal degradation, and it modulates the intracellular trafficking and cytotoxicity of cisplatin and copper, elements whose divalent ions display similar sulfur-binding properties [65].

Unsurprising, the metal mixture significantly induced the expression of MT10B and GST transcripts. In mammalian tissues, millimolar concentrations of glutathione (GSH) account for more than 90% of the total non-protein sulfur and the metal-induced GSH depletion (GSSG) is expected to increase the ROS formation and the oxidation of other cell thiols [66]. Metallothioneins are small thiol-rich metal-binding proteins which contribute to the homeostasis of essential metals, radical scavenging and protection against non-essential metal ions; hence, they are of general importance also in the cell proliferation and differentiation processes [67]. Differential metal binding affinity explains both the displacement of essential elements from MT (Hg^2+^>Cu^+^>Cd^2+^>Zn^2+^) and enhanced MT expression via the metal-regulatory transcription factor 1, MTF1, a nucleocytoplasmic shuttling protein which accumulates in the nucleus upon metal exposure and binds to promoters containing a metal-responsive element [68]–[69]. Different MT isoforms exist in *Mytilus* species and mussels from metal-contaminated sites commonly display increased MT levels [70]–[71]. MT10 isoforms are constitutively expressed but also induced by Cu, Zn, Cd and, partly, Hg whereas MT20 has very low basal expression, markedly increased after exposure to Cd and, somewhat, Hg and Cu [56], [72]–[74]. The DNA microarray and qRT-PCR analyses of MT expression performed in the gills of the treated mussels are consistent with the current knowledge and confirmed isoform-specific expression patterns.

The dose-related increases of MT10 and MT20 transcripts, and the over-expression of ferritin at the 200 nM dose, furthermore suggest enhanced scavenging of free metal ions from the cytosol. Ferritin is an ubiquitous multimeric protein with large metal storage capacity and, likewise MTs, its metal-induced expression depends on metal response elements located upstream the transcribed region. Atypical ferritins with signal peptides were detected in some molluscs and iron-withholding via ferritin up-regulation is supposed to improve the bivalve immune responses against microbial infections [75]. In the Baltic blue mussel Mytilus trossulus, the highest ferritin content was recorded in the hepatopancreas followed by the gills [76]: the large amounts of Cd and Fe associated to ferritin detected in mussels from metal-contaminated sites support the influence of toxic metals on the intracellular cycling of redox metals such as iron and copper.

On the other hand, transcripts grouped in the gene categories of Cell motility and intracellular trafficking, Cell adhesion and extracellular matrix, Immunity and inflammation were frequently under-expressed in the treated mussels. Deleterious effects of Cu and Cd on the cytoskeleton of mussel haemocytes have been reported and are likely associated to the depletion of antioxidant cell defenses (e.g. total thiols) and direct protein damage [77]–[78]. As regards the immune-related genes, transcripts denoting mytimacin-2 and 4, defensin-like protein 298, a macrophage expressed protein, fibrinogen-related protein 7, hemagglutinin/amebocyte aggregation factor precursor and the C1q domain containing proteins (MgC1q89, MgC1q47, MgC1q98) were under-expressed in the treated mussels, a fact confirming the weakening of the immune defenses in bivalves naturally or experimentally contaminated [79]–[80].

Gene categories less represented in these transcriptional profiles are not of minor importance, for instance those of Replication, transcription and repair, Signal transduction, Cell cycle and apoptosis, as they group DNA/RNA-interacting proteins, histones and several molecules essential to cross-talking biochemical pathways. At the highest metal dose, we found over-expressed some putative MAPK signalling elements (e.g. DUSP7 and RACK1), nucleolar p130 (NOLC1) and suppressor of cytokine signalling (SOCS2) whereas a possible protein kinase C binding protein (NELL1) was under-expressed. Besides, the under-expression of transcripts with DEAD motifs or denoting proteins involved in cell cycle regulation (INO80, PCNA) was associated to the over-expression of transcripts such as GADD45, CASP3, CASP7 and TCTP among others, in agreement with the evidence of MN and NA and suggestive of unbalance toward cell death and cell replacement.

A number of genes differentially expressed in the present study (individual mussel gills) showed expression trends similar to those previously detected in the digestive gland of mussels exposed to the 100 nM metal dose [32]: the up-regulated MT10B and GST and, conversely, the down-regulated DEAD-box protein Myt01-007F09, apoptosis inhibitor Myt01-007H08, alpha tubulin Myt01-016D09, actin Myt01-015H12 and C1q domain containing protein Myt01-015H10, among others. Not forgetting the tissue specificity of gene transcription, these expression changes exemplify common elements of the molecular signature induced by the toxic metals.

We have also confirmed selected expression trends by quantitative real time PCR. The expression values of probes for the mussel elongation factors EF1/2 were differentially expressed in our expression data sets and, therefore, not appropriate as endogenous reference genes. According to earlier experience, we used the ribosomal 18S gene as reference gene to calculate the relative expression values of two MT isoforms, ferritin, three heat shock proteins, SQSTM1 and GADD45 gamma sequences (with suitable proportions of 18S competimers to reduce the abundance of r18S sequences into the same scale of the transcripts under testing).

### Overview of the Gill Cell Response to Equi(nano)molar Combinations of Cd, Cu and Hg

Based on the empirical lists of differentially expressed genes, the model proposed in Figure 7 outlines the structural and functional impairments determined by the metal mixtures in the mussel gill cells. In the living organisms, specialized mechanisms govern the uptake, compartmentalization and extrusion of essential metal elements whereas the toxicity of non-essential metals often depends on molecular mimicry, competition for ligand binding and intrinsic ability to induce oxidative stress. In excessive amounts, both essential and non-essential metals can cause cell damage: disturbance to the calcium homeostasis, intracellular signalling and transcription factor regulation as well as boosted oxidative damage to various cell components are common determinants of the metal ion toxicity.

In normal conditions, the metal trafficking between and within the cells is tightly regulated by a complex network of potential metal ligands whose binding constants are of critical importance for many biological processes. For instance, the Ca sensor calmodulin takes part to signalling pathways relevant for cell excitability, exocytosis and motility, and depend on cytosolic increases of Ca at least ten folds the ∼100 nM resting levels [81]. The interplay of GSH and thiol-rich proteins, apoproteins, and organic molecules such as porphyrins and pterins, assures the proper intracellular concentrations of each element, surprisingly close to zero for free metal ions of essential metals such as Cu and Zn [69]. The excessive presence of metal ions can lead to competitive inhibition, saturation or inactivation of metal binding proteins whereas excessive production of reactive species may impair reactive oxygen/nitrogen species (ROS/RNS) signalling, cause untargeted oxidative lesions, and possibly increase the turnover of specific molecules such as MTs [34], [82]–[84].

Divalent Cu ions can enter the cells through CTR and proton-coupled DCT (high-affinity copper transporters and low-affinity divalent cation transporters, respectively) or by exploiting epithelial Na channels (ENaC). In the ascidian *A. sydneiensis* samea, a DCT-like membrane transporter has been reported to mediate vanadium accumulation at exceptional levels in the vacuoles of the signet ring cells, a specific haemocyte subpopulation [85]. ENaC mediates the apical ion entry in kidney tubules and is tightly regulated in humans [86] whereas in the cnidarian *H. magnipapillata* a similar protein channel, directly gated by *Hydra* neuropeptides, surprisingly displayed high Ca ion permeability [87]. Cadmium, and Hg likewise, can enter the cells via substitutive binding to voltage-dependent Ca channels, zinc/iron-regulated transporters (Z/I-RT), Cu transporters, or by receptor-mediated endocytosis via DCT and transferrin systems [69], [88]. Moreover, ATP-dependent uptake of methyl Hg/cysteine complexes can occur via neutral amino acid transporters such as ASCT2 by molecular mimicry of methionine [34].

In the gills of mussels exposed to the highest metal doses, a membrane magnesium transporter (MMgT1) and a taurine transporter were significantly over-expressed, likewise an epsin 1-related transcript (EPN1) at the 200 nM metal dose. Epsins are adaptor molecules involved in both transporter/receptor endocytosis and intracellular signalling whereas distinct epsin-like proteins participate in the trans-Golgi network/endosomes transport [89]. Regarding the intracellular metal trafficking, Cu ions for instance can be passed to chaperones which escort them into the Golgi (ATOX1, P-type ATPases), to apo-SOD1 (CCS) or to the mitochondria. In the latter case, Cu^+^ can be incorporated into the cytochrome c oxidase (COX). Transcripts denoting COX and cytochrome b were mostly down-regulated in the gills of the treated mussels, and suggest impaired functioning of the respiratory chain. Inserted in metalloprotein complexes, Cu^+^ can be released from the basolateral membrane via trans-Golgi network by Cu/Cl symporters, Cu^+^ATPase and exocytosis or, instead, be moved forward the endoplasmic reticulum [69], [90]. The trafficking between these membranous cell compartments is regulated by Rab members of the small GTPase superfamily, and by components of Sec complexes which translocate proteins across and within the endoplasmic reticulum. In addition to SEC 61 and 63, mussel transcripts similar to RAB 6 were significantly up-regulated at the 200 nM metal dose.

Even weakly genotoxic metals such as Cd can indirectly contribute to oxidative cell stress by displacing Fe and Cu ions from ferritin and other proteins whereas metal-induced disruption of interconnected signalling cascades largely depend on to the displacement of Ca ions from calmodulin, membrane Ca^++^-ATPase and protein kinases centrally involved in the regulation of cell cycle, survival responses and also carcinogenesis [33], [81]. Cadmium can displace Zn from MTs or mimic Zn in the active sites of enzymes (e.g. metallo-proteinases, lyases, dehydrogenases, SOD) and, therefore, impair the catalytic, inhibitory, or accessory Zn functions in kinases/phosphatases and zinc-finger proteins [84]. *In vitro* incubation of mussel haemocytes with divalent Hg and Cu ions (50 µM) rapidly increased the cytosolic Ca levels and caused lysosomal membrane destabilization via activation of Ca(2+)-dependent phospholipase A2 (cPLA2) [91]. Different dose range and exposure conditions or potential antagonist effects among the tested metals may explain why the mussel probe related to cPLA2 (Myt01-014H05) was not significantly modulated in the gills of mussels exposed to the metal mixture.

According to the Fenton reaction, any excessive availability of Fe and Cu ions generates hydroxyl radicals (^•^OH) and secondary reactive species which amplify the oxidative damage to lipids, carbohydrates, proteins, nucleic acids and possibly trigger the so called nitrosative stress [92]–[93]. For instance, the reaction of the superoxide radical (O_2_^•−^) with nitric oxide (NO^•^) produces the short-lived oxidant peroxynitrite anion (ONOO^−^). Inside the nucleus, oxidative lesions and DNA strand breaks increase the probability of mutagenic effects [94]. Typical lipid peroxidation products such as malondialdehyde and 4-hydroxy-2-nonenal have also been associated to the formation of DNA adducts, mutations and degenerative diseases [95]. Antioxidant enzymes (SOD, catalases, glutathion peroxidases and reductases), radical scavengers (e.g. glutathion, melatonin) and physiological metal ligands mentioned above represent the main defence line against the metal-induced ROS/RNS. Actually, the genes mostly up-regulated in the gill of the treated mussels (protein chaperones, MT isoforms and GST, among others) and increased DNA damage levels support the evidence of metal-induced oxidative stress. Though not investigated in this study, somatic mutations possibly resulting from misreplication and misrepair at diffused lesion sites are expected to reduce the efficiency of the body processes and reproductive capacity, and might also impact subsequent generations.

### Conclusions

The present study has essentially described early transcriptional changes induced *in vivo* in the mussel gills by a reference combination of toxic and genotoxic metals, namely Cd, Cu and Hg, delivered as water-soluble metal salts at equal nanomolar concentration. We anchored the interpretation of the gene expression profiles to the occurrence of DNA damage measured in the same gill samples in parallel, and added some confirmatory elemental metal analyses. The whole results substantiate previous findings and point to a large number of mussel gene functions whose identity and functional characterization requires future investigation. In perspective, the study of combined metal toxicity and toxicity thresholds could be extended by pointing to the most promising candidate markers, especially those bridging different aspects of the cell and organism response to common pollutants, and to the understanding of synergistic or antagonistic effects of single mixture components.

### Expression Data and Supporting Information

The raw MytArray expression data described above are deposited at the www.ncbi.nlm.nih.gov/geo repository (series GSE41218).

## Supporting Information

Figure S1 **Hierarchical clustering of the normalized fluorescence data referred to three mussels per treatment dose (Pearson correlation).** Twin datasets (e.g. 6A and 6B) derive from the hybridization of the same sample, e.g. gill RNA processed from mussel number six, with dye reversal on Mytarray 1.0.
(TIF)

Figure S2 **Unsupervised hierarchical clustering and relative expression trends of unknown genes differentially expressed in the mussel gills at least in one treatment dose (extension of** **Figure 4** **, identity codes refers to the MytArray probes).**
(TIF)

Table S1 **Individual frequency of gill cells with MN or NA in offshore mussels collected in late spring (A) and marketed mussels collected in winter (B) following 48 h exposure to 0–200 nM doses of combined metals.** Mean (N = 5) and standard deviation per dose point are reported in bold (* and *** indicate significant increases at p<0.05 and 0.001 compared to the control mussels).
(PDF)

Table S2 **Expression values of genes differentially expressed in the gills of individual mussels treated with the 50 nM metal dose (SAM, One class).** Identity codes, best sequence similarity, assigned functional category, relative expression values (log2 test/reference ratio) and inter-individual medians are reported.
(PDF)

Table S3 **Expression values of genes differentially expressed in the gills of individual mussels exposed to the 100 nM metal dose (SAM, One class).** Identity codes, best sequence similarity, assigned functional category, relative expression values (log2 test/reference ratio) and interindividual medians are reported.
(PDF)

Table S4 **Expression values of genes differentially expressed in the gills of individual mussels exposed to the 200 nM metal dose (SAM, One class).** Identity codes, best sequence similarity, assigned functional category, relative expression values (log2 test/reference ratio) and interindividual medians are reported.
(PDF)

Table S5 **Identity and expression values of genes differentially expressed in the gills of individual mussels exposed to the combined metals (genes in common between doses, extracted from Tables S2, S3, S4).**
(PDF)

Table S6 **Relative quantification values (RQ) of selected mussel transcripts significantly modulated after exposure to the combined metals.** Pooled gill RNAs from both treated and control mussels were tested in triplicate according to the 2−ΔΔCt method and considering 18S rRNA as endogenous control gene.
(PDF)
