# Supplementary figures and images for: DNA Damage and Transcriptional Changes in the Gills of Mytilus galloprovincialis Exposed to Nanomolar Doses of Combined Metal Salts (Cd, Cu, Hg)
Source: PLoS One. 2013 Jan 23;8(1):e54602. doi: 10.1371/journal.pone.0054602 (PMC3552849; doi:10.1371/journal.pone.0054602)

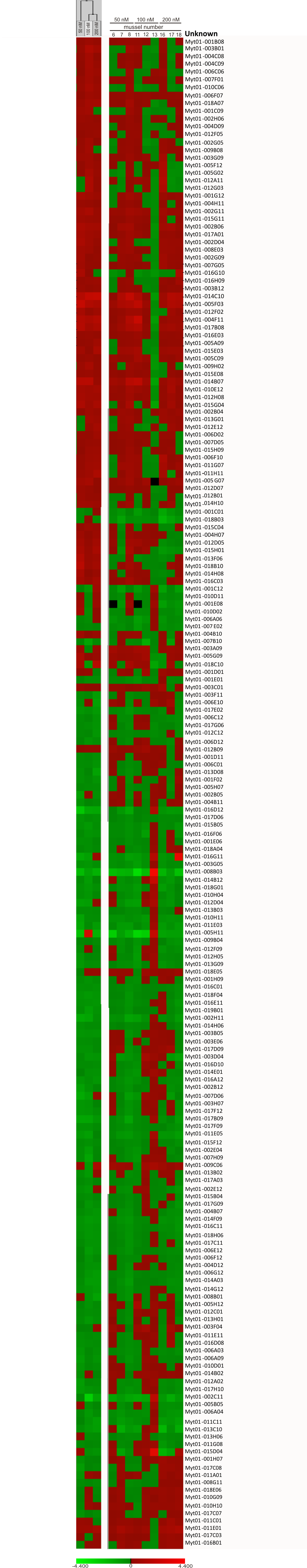

Supplement: Figure S2 — Unsupervised hierarchical clustering and relative expression trends of unknown genes differentially expressed in the mussel gills at least in one treatment dose (extension of Figure 4 , identity codes refers to the MytArray probes). (TIF) [file pone.0054602.s002.tif]
