# Supplementary material for: DNA Damage and Transcriptional Changes in the Gills of Mytilus galloprovincialis Exposed to Nanomolar Doses of Combined Metal Salts (Cd, Cu, Hg)
Source: PLoS One. 2013 Jan 23;8(1):e54602. doi: 10.1371/journal.pone.0054602 (PMC3552849; doi:10.1371/journal.pone.0054602)
Supplement: Table S1 — Individual frequency of gill cells with MN or NA in offshore mussels collected in late spring (A) and marketed mussels collected in winter (B) following 48 h exposure to 0–200 nM doses of combined metals. Mean (N = 5) and standard deviation per dose point are reported in bold (* and *** indicate significant increases at p<0.05 and 0.001 compared to the control mussels). (PDF) [file pone.0054602.s003.pdf]

Table S1. Individual frequency of gill cells with MN or NA in offshore mussels collected in late spring (A) and marked mussels collected in winter (B) following 48 h exposure to 0-200 nM doses of combined metals.

A

Dose	Mussel No.	‰ frequency of MN	‰ frequency of NA
0 nM	1	3.29	4.19
	2	2.21	7.36
	3	2.65	7.37
	4	1.02	6.44
	5	0.34	7.38
		**1.90 ± 1.21**	**6.55 ± 1.38**
50 nM	6	2.99	1.20
	7	2.81	10.67
	8	2.79	18.74
	9	3.39	14.81
	10	3.90	7.02
		***3.18 ± 0.47**	*****10,49 ± 6.80**
100 nM	11	5.56	12.32
	12	7.22	11.28
	13	8.31	7.00
	14	2.21	8.49
	15	4.49	14.36
		*****5.56 ± 2.38**	*****10.69 ± 2.95**
200 nM	16	5.76	7.82
	17	4.88	10.20
	18	11.73	21.32
	19	5.98	9.96
	20	9.30	27.29
		*****7.53 ± 2.89**	*****15.32 ± 8.52**

B

Dose	Mussel No.	‰ frequency of MN	‰ frequency of NA
0 nM	21	0.94	2.83
	22	0.94	2.83
	23	0.98	0.98
	24	0.72	2.15
	25	0.45	1.59
		**0.81 ± 0.22**	**2.08 ± 0.80**
50 nM	26	1.13	4.91
	27	1.52	5.32
	28	2.67	4.13
	29	1.95	6.51
	30	3.14	6.77
		*****2.08 ± 0.82**	*****5.53 ± 1.10**
100 nM	31	3.27	7.54
	32	1.71	10.02
	33	2.02	9.19
	34	2.93	8.56
	35	2.68	8.75
		*****2.52 ±0.64**	*****8.813 ± 0.91**
200 nM	36	3.52	6.81
	37	2.53	10.37
	38	3.08	5.50
	39	2.96	5.18
	40	2.14	6.41
		*****2.85 ± 0.53**	*****6.86 ± 2.07**

Mean (N=5) and standard deviation per dose point are reported in bold (* and *** indicate significant increases at $p < 0.05$ and 0.001 compared to the control mussels).
