# Supplementary material for: DNA Damage and Transcriptional Changes in the Gills of Mytilus galloprovincialis Exposed to Nanomolar Doses of Combined Metal Salts (Cd, Cu, Hg)
Source: PLoS One. 2013 Jan 23;8(1):e54602. doi: 10.1371/journal.pone.0054602 (PMC3552849; doi:10.1371/journal.pone.0054602)
Supplement: Table S2 — Expression values of genes differentially expressed in the gills of individual mussels treated with the 50 nM metal dose (SAM, One class). Identity codes, best sequence similarity, assigned functional category, relative expression values (log2 test/reference ratio) and inter-individual medians are reported. (PDF) [file pone.0054602.s004.pdf]

Table S2. Expression values of genes differentially expressed in the gills of individual mussels treated with the 50 nM metal dose (SAM, One Class).
Identity codes, best sequence similarity, assigned functional category, relative expression values (log2 test/reference ratio) and inter-individual medians are reported.

Mytarray 1.0 ID	Mytibase ID	Description	Functional category	Expression value (log2) Mussel number: 6	7	8	Median	Fold change
**Over-expressed**								
Myt01-003A09	MGC01531	without similarity		0.65	0.71	-0.12	0.65	1.57
Myt01-016G09	MGC00670	heat shock protein 90 [Mytilus galloprovincialis]	protein folding, turnover & degradation	0.64	0.51	0.03	0.51	1.42
Myt01-011G05	MGC00301	small heat shock protein 24.1 [Mytilus galloprovincialis]	protein folding, turnover & degradation	0.78	0.05	0.50	0.50	1.42
Myt01-003C09	MGC00100	eukaryotic translation initiation factor 5A [Branchiostoma belcheri]	translation	0.50	0.53	0.00	0.50	1.42
Myt01-005D09	MGC01749	poly(A)-binding protein [Spisula solidissima]	translation	0.44	0.60	0.16	0.44	1.35
Myt01-015D04	MGC00261	hypothetical protein BRAFLDRAFT_100988 [Branchiostoma floridae]		0.26	0.42	2.76	0.42	1.34
Myt01-003F04	MGC01593	without similarity		0.41	0.71	0.04	0.41	1.33
Myt01-018G09	MGC03464	GTP-binding protein 4 [Xenopus tropicalis]	signal transduction	0.39	0.47	0.03	0.39	1.31
Myt01-014G05	MGC00222	C1q domain containing protein MgC1q98 [Mytilus galloprovincialis]	immunity & inflammation	0.39	0.15	0.46	0.39	1.31
Myt01-012B09	MGC02565	without similarity		0.37	0.57	0.09	0.37	1.29
Myt01-005H12	MGC00440	hypothetical protein CHLREDRAFT_181491 [Chlamydomonas reinhardtii]		0.93	-0.02	0.32	0.32	1.25
Myt01-003C01	MGC01553	without similarity		0.10	0.41	0.27	0.27	1.21
Myt01-008C07	MGC01455	elongation factor 2b [Drosophila melanogaster]	translation	0.27	0.79	0.08	0.27	1.20
Myt01-014B02	MGC02829	without similarity		0.21	0.34	0.26	0.26	1.20
Myt01-012D05	MGC02603	without similarity		0.53	0.24	0.18	0.24	1.18
Myt01-012B10	MGC02567	BAT2 domain containing 1-like [Saccoglossus kowalevskii]	cell cycle & apoptosis	0.41	0.21	0.21	0.21	1.16
Myt01-011G08	MGC02503	without similarity		0.13	0.19	0.42	0.19	1.14
Myt01-014D10	MGC01476	precollagen-P [Mytilus galloprovincialis]	cell adhesion & extracellular matrix	0.37	0.17	0.06	0.17	1.13
**Under-expressed**								
Myt01-015C02	MGC01307	alpha tubulin [Pinctada fucata]	cell motility & intracellular trafficking	-0.51	-0.10	-0.15	-0.15	1.11
Myt01-019B05	MGC10007	NADH dehydrogenase subunit 1 [Mytilus edulis]	metabolism & ion homeostasis	-0.19	-0.19	-0.54	-0.19	1.14
Myt01-010A03	MGC10011	cytochrome b [Mytilus galloprovincialis]	metabolism & ion homeostasis	-0.40	-0.23	-0.24	-0.24	1.18
Myt01-002D03	MGC01471	histone H3, partial [Reishia clavigera]	replication& transcription	-0.21	-0.26	-0.24	-0.24	1.18
Myt01-001C01	MGC01352	without similarity		-0.83	-0.25	-0.03	-0.25	1.19
Myt01-009H08	MGC00918	GalNAc/Gal-specific lectin [Crenomytilus grayanus]	signal transduction	-0.28	-0.31	-0.61	-0.31	1.24
Myt01-011C05	MGC10000	cytochrome b [Mytilus galloprovincialis]	metabolism & ion homeostasis	-0.23	-0.38	-0.45	-0.38	1.30
Myt01-016G10	MGC03163	without similarity		-0.46	-0.20	-0.59	-0.46	1.38
Myt01-015B09	MGC02960	incilarin A [Haliotis discus discus]	signal transduction	-0.67	-0.15	-0.86	-0.67	1.59
