# Supplementary material for: DNA Damage and Transcriptional Changes in the Gills of Mytilus galloprovincialis Exposed to Nanomolar Doses of Combined Metal Salts (Cd, Cu, Hg)
Source: PLoS One. 2013 Jan 23;8(1):e54602. doi: 10.1371/journal.pone.0054602 (PMC3552849; doi:10.1371/journal.pone.0054602)
Supplement: Table S3 — Expression values of genes differentially expressed in the gills of individual mussels exposed to the 100 nM metal dose (SAM, One class). Identity codes, best sequence similarity, assigned functional category, relative expression values (log2 test/reference ratio) and interindividual medians are reported. (PDF) [file pone.0054602.s005.pdf]

Table S3. Expression values of genes differentially expressed in the gills of individual mussels treated with the 100 nM metal dose (SAM, One Class).
Identity codes, best sequence similarity, assigned functional category, relative expression values (log2 test/reference ratio) and inter-individual medians are reported.

Mytarray 1.0 ID	Mytibase ID	Description	Funtional category	Expression value (log2) Mussel number:				Fold change
				11	12	13	Median	
**Over-expressed**								
Myt01-007D04	MGC00749	sequestosome-1 [Harpegnathos saltator]	protein folding, turnover & degradation	2.65	4.15	3.20	3.20	9.20
Myt01-012A04	MGC02534	FK506-binding protein [Suberites domuncula]	protein folding, turnover & degradation	1.24	1.08	0.88	1.08	2.12
Myt01-014C10	MGC02858	without similarity		0.50	1.20	1.06	1.06	2.09
Myt01-010C12	MGC02297	glutathione S-transferase GSTpi1 [Mytilus galloprovincialis]	metabolism & ion homeostasis	0.04	1.05	1.14	1.05	2.07
Myt01-013C12	MGC02733	small heat shock protein 24.1 [Mytilus galloprovincialis]	protein folding, turnover & degradation	-0.01	1.33	1.00	1.00	2.00
Myt01-005F03	MGC02733	without similarity		0.53	1.06	0.94	0.94	1.92
Myt01-011G05	MGC00301	small heat shock protein 24.1 [Mytilus galloprovincialis]	protein folding, turnover & degradation	0.06	1.04	0.90	0.90	1.87
Myt01-011H08	MGC02331	precollagen-D [Mytilus galloprovincialis]	cell adhesion & extracellular matrix	-0.01	0.85	1.10	0.85	1.80
Myt01-014B11	MGC01310	heat shock protein 70 [Mytilus galloprovincialis]	protein folding, turnover & degradation	1.10	0.85	0.74	0.85	1.80
Myt01-011E01	MGC02468	without similarity		0.62	0.76	0.97	0.76	1.69
Myt01-016C08	MGC01659	metallothionein-10B [Mytilus galloprovincialis]	metabolism & ion homeostasis	-0.04	0.88	0.74	0.74	1.67
Myt01-012F06	MGC02640	taurine transporter [Mytilus galloprovincialis]	signal transduction	0.73	0.75	0.52	0.73	1.66
Myt01-003C09	MGC00100	eukaryotic translation initiation factor 5A [Branchiostoma belcheri]	translation	0.72	0.42	0.86	0.72	1.65
Myt01-003E08	MGC01515	small nuclear ribonucleoprotein polypeptide G protein [Crassostrea ariakensis]	translation	0.66	0.74	0.42	0.66	1.58
Myt01-016G09	MGC00670	heat shock protein 90 [Mytilus galloprovincialis]	protein folding, turnover & degradation	-0.09	0.61	0.85	0.61	1.53
Myt01-002B06	MGC00093	without similarity		0.61	0.69	0.30	0.61	1.52
Myt01-001E02	MGC01380	without similarity		0.84	0.04	0.58	0.58	1.50
Myt01-003G03	MGC01604	26S proteasome non-ATPase regulatory subunit 1 [Camponotus floridanus]	protein folding, turnover & degradation	0.59	0.08	0.56	0.56	1.47
Myt01-001H12	MGC01429	ADP-ribosylation factor 2, isoform CRA_b [Mus musculus]	signal transduction	0.07	1.27	0.52	0.52	1.43
Myt01-017B08	MGC03195	without similarity		0.51	0.62	0.41	0.51	1.42
Myt01-011G07	MGC00126	without similarity		0.74	0.11	0.50	0.50	1.41
Myt01-015E03	MGC03027	without similarity		0.39	0.48	0.48	0.48	1.39
Myt01-010B03	MGC02267	heat shock cognate 70 [Mytilus galloprovincialis]	protein folding, turnover & degradation	0.27	0.95	0.47	0.47	1.39
Myt01-009C06	MGC02152	without similarity		0.53	0.21	0.47	0.47	1.38
Myt01-002A11	MGC01439	small nuclear ribonucleoprotein associated protein B [Mustela putorius furo]	translation	0.31	0.58	0.46	0.46	1.37
Myt01-011B06	MGC02420	precollagen-P mRNA, complete cds [Mytilus galloprovincialis]	cell adhesion & extracellular matrix	-0.02	0.45	0.99	0.45	1.37
Myt01-018H05	MGC01435	ribosomal protein S12 [Pinctada maxima]	translation	0.59	0.11	0.44	0.44	1.36
Myt01-017B01	MGC03187	selenide, water dikinase [Harpegnathos saltator]	metabolism & ion homeostasis	0.44	0.52	0.34	0.44	1.36
Myt01-014B07	MGC02839	without similarity		0.29	0.70	0.44	0.44	1.35
Myt01-014B06	MGC02837	chaperonin subunit 7 [Epinephelus coioides]	protein folding, turnover & degradation	0.43	0.51	0.40	0.43	1.35
Myt01-018F02	MGC01507	ribosomal protein S27E [Mytilus galloprovincialis]	translation	0.42	0.35	0.46	0.42	1.34
Myt01-012B08	MGC02496	ribosomal protein L41 [Mus musculus]	translation	0.76	0.19	0.41	0.41	1.33
Myt01-016B01	MGC00162	structure-specific recognition protein and HMG box [Caenorhabditis elegans]		0.23	0.39	0.39	0.39	1.31
Myt01-008B04	MGC02006	precollagen-NG mRNA, complete cds [Mytilus galloprovincialis]	cell adhesion & extracellular matrix	0.00	0.39	0.73	0.39	1.31
Myt01-007D02	MGC07760	proteasome non-ATPase regulatory subunit, partial [Schistocerca gregaria]	protein folding, turnover & degradation	0.36	0.42	0.38	0.38	1.31
Myt01-002C12	MGC01468	membrane magnesium transporter 1 precursor [Danio rerio]	signal transduction	0.22	0.43	0.37	0.37	1.30
Myt01-018G03	MGC03456	eukaryotic translation initiation factor 3, subunit B [Xenopus tropicalis]	translation	0.39	0.37	0.31	0.37	1.29

Myt01-004F11	MGC01693	without similarity		0.18	0.38	0.35	0.35	1.27
Myt01-008E03	MGC02063	without similarity		0.40	0.35	0.26	0.35	1.27
Myt01-017C08	MGC09187	without similarity		0.39	0.34	0.21	0.34	1.27
Myt01-003A09	MGC01531	without similarity		0.24	0.42	0.33	0.33	1.25
Myt01-007E03	MGC01901	PAN2 poly specific ribonuclease subunit-like protein [Mustela putorius furo]	replication, transcription & repair	0.32	0.37	0.23	0.32	1.25
Myt01-011H03	MGC02517	c-24(28) sterol reductase [Aspergillus fumigatus Af293]	metabolism & ion homeostasis	0.32	0.17	0.37	0.32	1.24
Myt01-018E06	MGC03417	without similarity		0.31	0.23	0.39	0.31	1.24
Myt01-015F10	MGC00476	cold shock domain protein [Chlamys farreri]	replication, transcription & repair	0.31	0.54	0.08	0.31	1.24
Myt01-005G07	MGC01775	uncharacterized protein LOC100869204 isoform 2 [Apis florea]		0.07	0.30	0.49	0.30	1.24
Myt01-011A01	MGC02390	without similarity		0.36	0.10	0.29	0.29	1.23
Myt01-017C03	MGC00105	without similarity		0.26	0.40	0.28	0.28	1.22
Myt01-010G09	MGC02371	without similarity		0.28	0.18	0.37	0.28	1.21
Myt01-017H04	MGC02446	small nuclear ribonucleoprotein Sm D1-like [Nasonia vitripennis]	translation	0.27	0.18	0.30	0.27	1.21
Myt01-002G11	MGC01510	without similarity		0.55	0.21	0.27	0.27	1.21
Myt01-012F11	MGC01731	elongation factor 1 gamma, putative [Ixodes scapularis]	translation	0.27	0.30	0.16	0.27	1.21
Myt01-010H10	MGC02388	without similarity		0.26	0.27	0.41	0.27	1.20
Myt01-011C01	MGC02429	high mobility group protein D [Glossina morsitans morsitans]		0.23	0.62	0.26	0.26	1.20
Myt01-011C02	MGC02430	p8 nuclear protein [Ixodes scapularis]	replication, transcription & repair	0.10	0.42	0.26	0.26	1.20
Myt01-015A05	MGC00706	ribosomal protein S26 [Lepidochitona cinerea]	translation	0.37	0.26	0.17	0.26	1.20
Myt01-002G09	MGC01508	without similarity		0.56	0.26	0.23	0.26	1.20
Myt01-017B11	MGC00983	defender against apoptotic cell death 1 [Argopecten irradians]	cell cycle & apoptosis	0.24	0.26	0.44	0.26	1.20
Myt01-007H10	MGC01969	receptor for activated C-kinase [Pinctada fucata]	signal transduction	0.66	0.25	0.20	0.25	1.19
Myt01-018E09	MGC03425	THO complex subunit 5 homolog B [Xenopus laevis]	replication, transcription & repair	0.25	0.24	0.24	0.24	1.18
Myt01-007G05	MGC01939	without similarity		0.58	0.23	0.15	0.23	1.18
Myt01-007H02	MGC00848	proteasome subunit beta type-5-like [Strongylocentrotus purpuratus]	protein folding, turnover & degradation	0.47	0.23	0.16	0.23	1.17
Myt01-006H09	MGC04493	fascin [Haliotis diversicolor]	cell motility & intracellular trafficking	0.23	0.29	0.15	0.23	1.17
Myt01-001D03	MGC04135	ribosomal protein S26 [Ornithodoros parkeri]	translation	0.31	0.22	0.13	0.22	1.16
Myt01-001D01	MGC01366	without similarity		0.38	0.15	0.22	0.22	1.16
Myt01-001H07	MGC01424	without similarity		0.47	0.22	0.19	0.22	1.16
Myt01-012G04	MGC02654	microsomal glutathione S-transferase 3 [Pinctada martensi]	metabolism & ion homeostasis	0.13	0.62	0.22	0.22	1.16
Myt01-018E05	MGC03415	without similarity		0.16	0.21	0.47	0.21	1.16
Myt01-014G01	MGC02914	ribosomal protein rps27 [Arenicola marina]	translation	0.17	0.41	0.19	0.19	1.14
Myt01-009B08	MGC00336	hypothetical protein BRAFLDRAFT_74514 [Branchiostoma floridae]		0.35	0.16	0.18	0.18	1.13
Myt01-001D10	MGC01376	C-type lectin 5 [Chlamys farreri]	signal transduction	0.14	0.73	0.18	0.18	1.13
Myt01-016F03	MGC03147	cytochrome oxidase subunit 3 [Mytilus galloprovincialis]	metabolism & ion homeostasis	0.28	0.17	0.18	0.18	1.13
Myt01-004C05	MGC02203	alpha-2 macroglobulin family protein VIP [Homo sapiens]	protein folding, turnover & degradation	0.16	0.18	0.33	0.18	1.13
Myt01-006F10	MGC01826	without similarity		0.93	0.06	0.17	0.17	1.13
Myt01-010E12	MGC02332	without similarity		0.16	0.79	0.10	0.16	1.12
**Under-expressed**								
Myt01-017F04	MGC01209	ribosomal protein S24; MRP S24 [Mus musculus]	translation	-0.14	-0.14	-0.54	-0.14	1.10
Myt01-012G09	MGC02660	vacuolar protein sorting 26 homolog B-like [Saccoglossus kowalevskii]	cell motility & intracellular trafficking	-0.16	-0.11	-0.37	-0.16	1.12
Myt01-016C09	MGC00117	beta-microseminoprotein [Xenopus (Silurana) tropicalis]	immunity & inflammation	-0.16	-0.18	-0.50	-0.18	1.14
Myt01-015F12	MGC01896	without similarity		-0.17	-0.19	-0.47	-0.19	1.14

Myt01-013H06	MGC00358	without similarity		-0.39	-0.21	-0.20	-0.21	1.16
Myt01-006H01	MGC01796	40S ribosomal protein RPS14 [Novocrania anomala]	translation	-0.18	-0.61	-0.22	-0.22	1.16
Myt01-006A07	MGC03983	peptidylprolyl isomerase protein, cyclophilin [Crassostrea gigas]	protein folding, turnover & degradation	-0.68	-0.07	-0.22	-0.22	1.16
Myt01-001A04	MGC01325	PACRG, partial [Xenopus laevis]	protein folding, turnover & degradation	-0.15	-0.22	-0.45	-0.22	1.16
Myt01-015A11	MGC00019	6-phosphogluconolactonase-like [Strongylocentrotus purpuratus]	metabolism & ion homeostasis	-0.22	-0.42	-0.17	-0.22	1.16
Myt01-014D10	MGC01476	precollagen-P [Mytilus galloprovincialis]	cell adhesion & extracellular matrix	-0.73	-0.09	-0.22	-0.22	1.17
Myt01-015H10	MGC03114	C1q domain containing protein MgC1q89 [Mytilus galloprovincialis]	immunity & inflammation	-0.23	-0.08	-0.52	-0.23	1.17
Myt01-015G01	MGC03065	ubiquitin [Artemia franciscana]	protein folding, turnover & degradation	-0.59	-0.03	-0.24	-0.24	1.18
Myt01-016A08	MGC00131	zona pellucida domain protein D [Haliotis rufescens]	development & reproduction	-0.13	-0.24	-0.59	-0.24	1.18
Myt01-006A03	MGC01810	without similarity		-0.24	-0.16	-0.44	-0.24	1.18
Myt01-009F04	MGC01541	ribosomal protein l17 [Haliotis discus discus]	translation	-0.45	-0.11	-0.24	-0.24	1.18
Myt01-018H06	MGC03479	multiple C2 and transmembrane domain-containing protein 1-like [Strongylocentrotus purpuratus]		-0.16	-0.24	-0.51	-0.24	1.18
Myt01-015B05	MGC02494	without similarity		-0.25	-0.18	-0.66	-0.25	1.19
Myt01-010D02	MGC02053	hypothetical protein BRAFLDRAFT_67696 [Branchiostoma floridae]		-0.25	-0.22	-0.53	-0.25	1.19
Myt01-006G12	MGC01838	without similarity		-0.10	-0.26	-0.37	-0.26	1.20
Myt01-016C12	MGC00089	without similarity		-0.07	-0.27	-0.43	-0.27	1.21
Myt01-013B09	MGC00050	selenium-binding protein [Chlamys farreri]	metabolism & ion homeostasis	-0.32	-0.18	-0.28	-0.28	1.21
Myt01-011B08	MGC01829	paramyosin, complete cds [Mytilus galloprovincialis]	cell motility & intracellular trafficking	-0.15	-0.28	-0.38	-0.28	1.21
Myt01-011G08	MGC02503	without similarity		-0.68	-0.28	-0.01	-0.28	1.22
Myt01-006A06	MGC00023	without similarity		-0.60	-0.19	-0.30	-0.30	1.23
Myt01-018G11	MGC00860	Integumentary mucin C.1 (FIM-C.1) [Xenopus laevis]	cell adhesion & extracellular matrix	-0.30	-0.24	-0.34	-0.30	1.23
Myt01-006E12	MGC04318	predicted protein-like [Saccoglossus kowalevskii]		-0.16	-0.30	-0.34	-0.30	1.23
Myt01-001G04	MGC01408	arginine kinase [Sepiella maindroni]	metabolism & ion homeostasis	-0.31	-0.26	-0.46	-0.31	1.24
Myt01-016C11	MGC03129	without similarity		-0.32	-0.30	-0.64	-0.32	1.25
Myt01-016A12	MGC00159	without similarity		-0.10	-0.33	-0.35	-0.33	1.25
Myt01-017F02	MGC01802	collagen alpha-1(XII) chain-like [Anolis carolinensis]	cell adhesion & extracellular matrix	-0.33	-0.01	-0.67	-0.33	1.25
Myt01-017H10	MGC03327	without similarity		-0.33	-0.19	-0.45	-0.33	1.26
Myt01-003G05	MGC01606	universal stress protein MSMEG_3950 [Clonorchis sinensis]		-0.56	-0.27	-0.33	-0.33	1.26
Myt01-015G12	MGC02989	precollagen-D [Mytilus galloprovincialis]	cell adhesion & extracellular matrix	-0.34	-0.64	-0.04	-0.34	1.26
Myt01-010D11	MGC02596	without similarity		-0.35	-0.17	-0.34	-0.34	1.27
Myt01-014G05	MGC00222	C1q domain containing protein MgC1q98 [Mytilus galloprovincialis]	immunity & inflammation	-0.85	-0.10	-0.34	-0.34	1.27
Myt01-003G12	MGC01613	INO80 complex subunit C-like [Danio rerio]	replication, transcription & repair	-0.16	-0.34	-0.40	-0.34	1.27
Myt01-002E12	MGC01488	without similarity		-0.10	-0.35	-0.71	-0.35	1.27
Myt01-012A02	MGC02531	without similarity		-0.36	-0.26	-0.50	-0.36	1.28
Myt01-013G09	MGC02798	without similarity		-0.59	-0.36	-0.18	-0.36	1.28
Myt01-019B03	MGC10004	cytochrome c oxidase subunit III (mitochondrion) [Mytilus galloprovincialis]	metabolism & ion homeostasis	-0.47	-0.38	-0.15	-0.38	1.30
Myt01-011H07	MGC02521	axonemal dynein light chain p33 [Haliotis discus discus]	cell motility & intracellular trafficking	-0.38	-0.21	-0.59	-0.38	1.30
Myt01-004B07	MGC01642	without similarity		-0.12	-0.39	-0.46	-0.39	1.31
Myt01-002H11	MGC01523	without similarity		-0.39	-0.27	-0.81	-0.39	1.31
Myt01-012F09	MGC01399	without similarity		-0.56	-0.24	-0.40	-0.40	1.32
Myt01-007E02	MGC00455	without similarity		-0.47	-0.27	-0.40	-0.40	1.32
Myt01-019B08	MGC02110	Mitochondrial-ND6 [Mytilus galloprovincialis]	metabolism & ion homeostasis	-0.21	-0.88	-0.41	-0.41	1.32
Myt01-012G05	MGC00349	transcription factor containing NAC and TS-N domains, putative [I. scapularis]	replication, transcription & repair	-0.48	-0.33	-0.41	-0.41	1.33

Myt01-008E07	MGC00055	pleiotrophin-like protein [Patella caerulea]	signal transduction	-0.74	-0.02	-0.41	-0.41	1.33
Myt01-014E05	MGC02886	collagen pro alpha-chain [Haliotis discus]	cell adhesion & extracellular matrix	-0.25	-0.41	-0.42	-0.41	1.33
Myt01-014A03	MGC05861	hypothetical protein BRAFLDRAFT_270517 [Branchiostoma floridae]		-0.15	-0.46	-0.42	-0.42	1.34
Myt01-017B09	MGC03196	without similarity		-0.42	-0.08	-0.81	-0.42	1.34
Myt01-005B05	MGC00027	without similarity		-0.43	-0.46	-0.39	-0.43	1.34
Myt01-014E06	MGC02887	mytimacin-4 [Mytilus galloprovincialis]	immunity & inflammation	-0.09	-0.43	-0.52	-0.43	1.35
Myt01-009B04	MGC02140	without similarity		-0.02	-0.44	-0.60	-0.44	1.36
Myt01-011E07	MGC02476	stress-associated endoplasmic reticulum protein 2 [Homo sapiens]	protein folding, turnover & degradation	-0.45	-0.52	-0.38	-0.45	1.37
Myt01-007F09	MGC01920	DEAD (Asp-Glu-Ala-Asp) box polypeptide 17 [Taeniopygia guttata]	replication, transcription & repair	-0.94	-0.46	-0.33	-0.46	1.38
Myt01-011H01	MGC02512	C1q domain containing protein MgC1q47 [Mytilus galloprovincialis]	immunity & inflammation	-0.62	-0.10	-0.49	-0.49	1.40
Myt01-014B12	MGC01827	hypothetical protein BRAFLDRAFT_86469 [Branchiostoma floridae]		-0.50	-0.71	-0.20	-0.50	1.41
Myt01-014F09	MGC02906	CG10903-PA [Strongylocentrotus purpuratus]		-0.50	-0.39	-0.60	-0.50	1.41
Myt01-018G01	MGC00020	without similarity		-0.85	-0.50	0.00	-0.50	1.42
Myt01-010H11	MGC02389	without similarity		-0.47	-0.59	-0.51	-0.51	1.43
Myt01-009G02	MGC02210	histone deacetylation protein Rxt3 [Glomerella graminicola M1.001]	replication, transcription & repair	-0.64	-0.22	-0.51	-0.51	1.43
Myt01-015H01	MGC02998	without similarity		-0.74	-0.54	-0.38	-0.54	1.45
Myt01-015C02	MGC01307	alpha tubulin [Pinctada fucata]	cell motility & intracellular trafficking	-0.32	-0.55	-0.66	-0.55	1.46
Myt01-010B10	MGC02276	alpha 1 type XII collagen short isoform precursor [Homo sapiens]	cell adhesion & extracellular matrix	-0.59	-2.63	0.34	-0.59	1.50
Myt01-015H12	MGC00110	actin [Mizuhopecten yessoensis]	cell motility & intracellular trafficking	-0.57	-0.59	-0.77	-0.59	1.50
Myt01-006H12	MGC00243	alpha-tubulin, partial [Nodipecten subnodosus]	cell motility & intracellular trafficking	-0.75	-0.23	-0.59	-0.59	1.51
Myt01-001C01	MGC01352	without similarity		-1.19	-0.61	-0.21	-0.61	1.52
Myt01-016D09	MGC00176	alpha tubulin [Pectinaria gouldii]	cell motility & intracellular trafficking	-0.34	-0.61	-0.63	-0.61	1.53
Myt01-001E08	MGC01385	without similarity		-0.94	-0.39	-0.63	-0.63	1.54
Myt01-011E05	MGC02473	without similarity		-0.64	-0.58	-0.88	-0.64	1.56
Myt01-006A04	MGC02476	without similarity		-0.42	-0.64	-0.68	-0.64	1.56
Myt01-002H12	MGC01524	leucine--tRNA ligase, cytoplasmic-like [Strongylocentrotus purpuratus]	translation	-0.14	-0.75	-0.68	-0.68	1.60
Myt01-016D12	MGC00084	without similarity		-0.57	-0.75	-0.71	-0.71	1.63
Myt01-003D04	MGC01543	without similarity		0.07	-0.71	-1.03	-0.71	1.64
Myt01-002C11	MGC01465	without similarity		-0.74	-1.36	-0.76	-0.76	1.69
Myt01-013C05	MGC02449	nongradient byssal precursor [Mytilus edulis]	cell motility & intracellular trafficking	-0.23	-1.29	-0.79	-0.79	1.73
Myt01-011E03	MGC02470	without similarity		-0.80	-0.46	-0.83	-0.80	1.75
Myt01-011C11	MGC02439	Q/N-rich domain Prion like protein PQN-75 (pqn-75) [Caenorhabditis elegans]		-0.83	-0.65	-1.01	-0.83	1.78
Myt01-003E07	MGC00175	beta tubulin [Chlamys farreri]	cell motility & intracellular trafficking	-1.04	-0.71	-0.87	-0.87	1.82
Myt01-001C12	MGC01365	hypothetical protein BRAFLDRAFT_68569 [Branchiostoma floridae]		-1.05	-0.17	-0.92	-0.92	1.89
Myt01-005H11	MGC06190	without similarity		-1.06	-0.93	-0.92	-0.93	1.90
Myt01-018B03	MGC03362	without similarity		-1.65	-1.08	-0.58	-1.08	2.12
Myt01-013C10	MGC01399	without similarity		-1.74	-0.43	-1.42	-1.42	2.68
Myt01-007H08	MGC01966	inhibitor of apoptosis 1 [Gallus gallus]	cell cycle & apoptosis	-1.27	-1.71	-1.65	-1.65	3.14
Myt01-008B03	MGC02005	without similarity		-1.69	-0.88	-2.08	-1.69	3.23
