# Supplementary material for: DNA Damage and Transcriptional Changes in the Gills of Mytilus galloprovincialis Exposed to Nanomolar Doses of Combined Metal Salts (Cd, Cu, Hg)
Source: PLoS One. 2013 Jan 23;8(1):e54602. doi: 10.1371/journal.pone.0054602 (PMC3552849; doi:10.1371/journal.pone.0054602)
Supplement: Table S4 — Expression values of genes differentially expressed in the gills of individual mussels exposed to the 200 nM metal dose (SAM, One class). Identity codes, best sequence similarity, assigned functional category, relative expression values (log2 test/reference ratio) and interindividual medians are reported. (PDF) [file pone.0054602.s006.pdf]

Table S4. Expression values of genes differentially expressed in the gills of individual mussels treated with the 200 nM metal dose (SAM, One Class).
Identity codes, best sequence similarity, assigned functional category, relative expression values (log2 test/reference ratio) and inter-individual medians are reported.

Mytarray 1.0 ID	Mytibase ID	Description	Funtional category	Expression value (log2)				Fold change
				Mussel number:				
Over-expressed				16	17	18	Median	
Myt01-007D04	MGC00749	sequestosome-1 [Harpegnathos saltator]	protein folding, turnover & degradation	5.19	4.28	3.77	4.28	19.49
Myt01-010C12	MGC02297	glutathione S-transferase GSTpi1 [Mytilus galloprovincialis]	metabolism & ion homeostasis	1.95	1.77	1.11	1.77	3.42
Myt01-016C08	MGC01659	metallothionein-10B [Mytilus galloprovincialis]	metabolism & ion homeostasis	2.19	0.77	1.75	1.75	3.35
Myt01-014C10	MGC02858	without similarity		1.19	1.73	1.81	1.73	3.31
Myt01-017B11	MGC00983	defender against apoptotic cell death 1 [Argopecten irradians]	cell cycle & apoptosis	1.51	0.74	1.46	1.46	2.76
Myt01-014B07	MGC02839	without similarity		1.35	1.38	0.30	1.35	2.55
Myt01-016G09	MGC00670	heat shock protein 90 [Mytilus galloprovincialis]	protein folding, turnover & degradation	1.28	1.28	1.19	1.28	2.42
Myt01-011G05	MGC00301	small heat shock protein 24.1 [Mytilus galloprovincialis]	protein folding, turnover & degradation	2.26	1.16	1.26	1.26	2.39
Myt01-014B11	MGC01310	heat shock protein 70 [Mytilus galloprovincialis]	protein folding, turnover & degradation	1.56	1.14	1.22	1.22	2.33
Myt01-004F11	MGC01693	without similarity		1.17	0.92	1.20	1.17	2.26
Myt01-015B09	MGC02960	incilarin A [Haliotis discus discus]	signal transduction	1.14	1.23	-0.20	1.14	2.20
Myt01-005F03	MGC01411	without similarity		1.81	1.11	0.89	1.11	2.17
Myt01-012D01	MGC00857	suppressor of cytokine signaling 2 [Haliotis discus discus]	signal transduction	1.42	0.52	1.09	1.09	2.13
Myt01-015D01	MGC00464	heat shock protein 90 [Mytilus galloprovincialis]	protein folding, turnover & degradation	1.47	1.08	0.97	1.08	2.12
Myt01-015F11	MGC03064	C1q domain containing protein MgC1q8 [Mytilus galloprovincialis]	immunity & inflammation	1.07	-0.19	1.16	1.07	2.10
Myt01-013C12	MGC02733	small heat shock protein 24.1 [Mytilus galloprovincialis]	protein folding, turnover & degradation	1.70	1.03	0.57	1.03	2.05
Myt01-012A04	MGC02534	FK506-binding protein [Suberites domuncula]	protein folding, turnover & degradation	1.03	1.01	0.90	1.01	2.01
Myt01-015F03	MGC03051	eukaryotic initiation factor 4A-II [Hydra magnipapillata]	replication, transcription & repair	0.97	0.69	1.08	0.97	1.96
Myt01-017B08	MGC03195	without similarity		1.23	0.67	0.92	0.92	1.89
Myt01-010B03	MGC02267	heat shock cognate 70 [Mytilus galloprovincialis]	protein folding, turnover & degradation	1.44	0.88	0.89	0.89	1.85
Myt01-011C02	MGC02430	p8 nuclear protein [Ixodes scapularis]	replication, transcription & repair	0.52	1.35	0.85	0.85	1.81
Myt01-015H01	MGC02998	without similarity		0.83	0.29	0.83	0.83	1.77
Myt01-015G04	MGC03070	without similarity		0.96	0.46	0.82	0.82	1.77
Myt01-018C10	MGC01375	without similarity		0.80	-0.75	1.35	0.80	1.74
Myt01-014B06	MGC02837	chaperonin subunit 7 [Epinephelus coioides]	protein folding, turnover & degradation	0.89	0.75	0.41	0.75	1.68
Myt01-012F06	MGC02640	taurine transporter [Mytilus galloprovincialis]	signal transduction	1.05	0.74	0.68	0.74	1.67
Myt01-001G11	MGC01415	RNA-binding protein 8A [Danio rerio]	replication, transcription & repair	0.72	0.89	0.39	0.72	1.64
Myt01-012D02	MGC00060	endochitinase-like [Bombus terrestris]	metabolism & ion homeostasis	0.24	0.71	0.74	0.71	1.63
Myt01-002B06	MGC00093	without similarity		0.90	0.71	0.66	0.71	1.63
Myt01-011H08	MGC02331	precollagen-D [Mytilus galloprovincialis]	cell adhesion & extracellular matrix	0.32	0.90	0.71	0.71	1.63
Myt01-006E09	MGC09113	axonemal dynein light chain p33 [Haliotis discus discus]	cell motility & intracellular trafficking	0.60	0.74	0.70	0.70	1.62
Myt01-003G03	MGC01604	26S proteasome non-ATPase regulatory subunit 1 [Camponotus floridanus]	protein folding, turnover & degradation	1.18	0.69	0.21	0.69	1.61
Myt01-018A07	MGC02814	without similarity		0.35	0.68	0.73	0.68	1.61
Myt01-013D11	MGC00220	ferritin [Mytilus galloprovincialis]	metabolism & ion homeostasis	1.22	0.64	0.66	0.66	1.58
Myt01-010E12	MGC02332	without similarity		0.66	0.57	0.69	0.66	1.58
Myt01-012A10	MGC02543	myosin heavy chain [Mytilus galloprovincialis]	cell motility & intracellular trafficking	0.77	0.66	0.06	0.66	1.58
Myt01-007D11	MGC01897	26S proteasome non-ATPase regulatory subunit 2 [Apis mellifera]	protein folding, turnover & degradation	0.73	0.66	0.48	0.66	1.58

Myt01-001H12	MGC01429	ADP-ribosylation factor 2, isoform CRA_b [Mus musculus]	signal transduction	1.10	0.65	0.41	0.65	1.57
Myt01-018B10	MGC03356	without similarity		1.41	0.36	0.64	0.64	1.56
Myt01-014G01	MGC02914	ribosomal protein rps27 [Arenicola marina]	translation	0.60	0.72	0.64	0.64	1.56
Myt01-003B01	MGC01535	without similarity		0.28	1.04	0.63	0.63	1.55
Myt01-002G11	MGC01510	without similarity		0.63	0.87	0.31	0.63	1.55
Myt01-015F10	MGC00476	cold shock domain protein [Chlamys farreri]	replication, transcription & repair	-0.07	0.63	0.74	0.63	1.55
Myt01-012D05	MGC02603	without similarity		0.65	0.18	0.62	0.62	1.54
Myt01-014G06	MGC02922	elongation factor 1 alpha [Mytilus edulis]	translation	0.62	0.62	0.70	0.62	1.54
Myt01-012G07	MGC02656	caffeoyl-CoA O-methyltransferase [Crocosphaera watsonii WH 0003]	metabolism & ion homeostasis	0.62	0.19	1.23	0.62	1.54
Myt01-016C03	MGC00112	without similarity		0.61	0.21	0.65	0.61	1.53
Myt01-001G12	MGC01416	without similarity		0.76	0.60	0.38	0.60	1.52
Myt01-002A11	MGC01439	small nuclear ribonucleoprotein associated protein B [Mustela putorius furo]	translation	0.52	0.69	0.60	0.60	1.51
Myt01-008C11	MGC02032	GDP dissociation inhibitor 2 [Danio rerio]	signal transduction	0.86	0.59	0.24	0.59	1.50
Myt01-012F11	MGC01731	elongation factor 1 gamma, putative [Ixodes scapularis]	translation	0.38	0.59	1.00	0.59	1.50
Myt01-005G07	MGC01775	uncharacterized protein LOC100869204 isoform 2 [Apis florea]		0.58	0.77	0.08	0.58	1.49
Myt01-015G01	MGC03065	ubiquitin [Artemia franciscana]	protein folding, turnover & degradation	0.69	0.24	0.57	0.57	1.48
Myt01-016G07	MGC00326	dual specificity protein phosphatase 7 [Danio rerio]	signal transduction	0.98	0.56	0.24	0.56	1.48
Myt01-015E03	MGC03027	without similarity		0.56	0.11	0.89	0.56	1.47
Myt01-012H08	MGC02672	without similarity		0.73	0.56	0.54	0.56	1.47
Myt01-014A02	MGC02814	elongation factor 1 alpha, partial [Mytilus californianus]	translation	0.52	0.54	0.70	0.54	1.46
Myt01-017A01	MGC03177	without similarity		0.78	0.54	0.40	0.54	1.45
Myt01-002D04	MGC01472	without similarity		0.89	0.54	0.22	0.54	1.45
Myt01-011G07	MGC00126	without similarity		0.21	0.53	0.61	0.53	1.44
Myt01-018F02	MGC01507	ribosomal protein S27E [Mytilus galloprovincialis]	translation	0.67	0.53	0.44	0.53	1.44
Myt01-018H05	MGC01435	ribosomal protein S12 [Pinctada maxima]	translation	0.15	0.52	0.71	0.52	1.44
Myt01-013D06	MGC02744	proteasome alpha type 2 [Haliotis discus discus]	protein folding, turnover & degradation	0.69	0.52	0.43	0.52	1.43
Myt01-011H11	MGC02526	without similarity		0.35	0.52	0.97	0.52	1.43
Myt01-014H12	MGC02947	pannexin 2 [Aplysia californica]	signal transduction	0.57	0.20	0.51	0.51	1.42
Myt01-012F02	MGC02637	without similarity		0.76	0.23	0.51	0.51	1.42
Myt01-012C06	MGC00759	splicing factor 3b, subunit 2, 145kDa, isoform CRA_f [Homo sapiens]	replication, transcription & repair	0.51	0.58	0.10	0.51	1.42
Myt01-010C09	MGC00153	ribosomal protein rpl7a [Arenicola marina]	translation	0.46	0.52	0.51	0.51	1.42
Myt01-012E08	MGC02625	small nuclear ribonucleoprotein D2-like protein [Chlamys farreri]	translation	0.51	0.66	0.36	0.51	1.42
Myt01-006C05	MGC01801	Growth arrest and DNA-damage-inducible protein 45 gamma [C. gigas]	cell cycle & apoptosis	1.10	0.40	0.50	0.50	1.42
Myt01-007F08	MGC01300	C-type lectin [Mytilus edulis]	signal transduction	0.38	0.50	0.50	0.50	1.41
Myt01-004F10	MGC03846	cytochrome oxidase subunit III [Mytilus galloprovincialis]	metabolism & ion homeostasis	0.21	0.71	0.50	0.50	1.41
Myt01-013F06	MGC02770	without similarity		1.13	0.33	0.49	0.49	1.41
Myt01-006B11	MGC00665	polyubiquitin [Anas platyrhynchos]	protein folding, turnover & degradation	1.67	0.38	0.49	0.49	1.40
Myt01-011G02	MGC00145	elongation factor 1 alpha [Mytilus edulis]	translation	0.32	0.49	0.58	0.49	1.40
Myt01-014H10	MGC00351	without similarity		0.54	0.48	0.41	0.48	1.40
Myt01-001C09	MGC01362	without similarity		0.48	0.64	-0.05	0.48	1.40
Myt01-012C07	MGC02586	cycling-dependent kinase 5 [Sphaerechinus granularis]	signal transduction	0.78	0.47	0.33	0.47	1.39
Myt01-006F10	MGC01826	without similarity		0.21	0.46	0.52	0.46	1.38
Myt01-005D09	MGC01749	poly(A)-binding protein [Spisula solidissima]	translation	0.46	0.62	-0.10	0.46	1.37

Myt01-013G01	MGC01774	without similarity		-0.03	0.58	0.46	0.46	1.37
Myt01-008E03	MGC02063	without similarity		0.58	0.46	0.10	0.46	1.37
Myt01-011E01	MGC02468	without similarity		0.41	0.46	0.77	0.46	1.37
Myt01-015H09	MGC00123	without similarity		0.05	0.43	0.47	0.43	1.34
Myt01-015B01	MGC01913	chondroitin proteoglycan 2 [Ascaris suum]	cell adhesion & extracellular matrix	0.02	0.43	0.43	0.43	1.34
Myt01-014H01	MGC02930	lactamase, beta 2 [Hydra magnipapillata]	metabolism & ion homeostasis	0.32	0.42	0.44	0.42	1.34
Myt01-013B10	MGC00157	ribosomal protein p0 [Mytilus galloprovincialis]	translation	0.42	0.29	0.88	0.42	1.34
Myt01-009H02	MGC02229	without similarity		0.42	0.79	0.19	0.42	1.34
Myt01-006C06	MGC00060	without similarity		0.01	0.63	0.42	0.42	1.34
Myt01-003G09	MGC00118	without similarity		0.41	0.50	0.13	0.41	1.33
Myt01-001E07	MGC00177	ribosomal protein L23a [Argopecten irradians]	translation	0.41	0.37	0.43	0.41	1.33
Myt01-001G09	MGC01413	testis-specific serine/threonine-protein kinase 4-like [Strongylocentrotus purpu	signal transduction	0.40	0.41	0.25	0.40	1.32
Myt01-019B05	MGC10007	NADH dehydrogenase subunit 1 [Mytilus edulis]	metabolism & ion homeostasis	0.02	0.40	0.51	0.40	1.32
Myt01-012H02	MGC01908	60S ribosomal protein L22-like [Strongylocentrotus purpuratus]	translation	0.40	0.16	0.43	0.40	1.31
Myt01-015G11	MGC00678	without similarity		0.48	0.39	0.31	0.39	1.31
Myt01-013D10	MGC02748	nucleosome assembly protein 1-like 1 [Danio rerio]	protein folding, turnover & degradation	0.39	0.44	0.20	0.39	1.31
Myt01-010C06	MGC02286	without similarity		0.28	0.77	0.39	0.39	1.31
Myt01-009D06	MGC00234	cyclophilin [Aiptasia pallida]	protein folding, turnover & degradation	0.38	0.19	0.42	0.38	1.31
Myt01-011C12	MGC02440	sec61alpha [Papilio xuthus]	protein folding, turnover & degradation	0.38	-0.04	0.43	0.38	1.30
Myt01-004H07	MGC00608	without similarity		0.46	0.10	0.38	0.38	1.30
Myt01-017B01	MGC03187	selenide, water dikinase [Harpegnathos saltator]	metabolism & ion homeostasis	0.37	0.52	0.10	0.37	1.30
Myt01-018G07	MGC03462	translocation protein SEC63 homolog [Rattus norvegicus]	protein folding, turnover & degradation	0.37	0.44	0.29	0.37	1.29
Myt01-007D01	MGC01885	aspartyl-tRNA synthetase 2, mitochondrial [Mustela putorius furo]	translation	0.43	0.37	0.34	0.37	1.29
Myt01-015C01	MGC02987	general transcription factor 3C polypeptide 1-like [Danio rerio]	replication, transcription & repair	0.30	0.62	0.37	0.37	1.29
Myt01-014D12	MGC00411	ribosomal protein rps19 [Crassostrea gigas]	translation	0.36	0.29	0.44	0.36	1.29
Myt01-005C08	MGC00571	60S ribosomal protein L5-like [Strongylocentrotus purpuratus]	translation	0.21	0.70	0.36	0.36	1.28
Myt01-007E09	MGC01910	6-phosphogluconate dehydrogenase, decarboxylating [Salmo salar]	metabolism & ion homeostasis	0.36	0.43	0.10	0.36	1.28
Myt01-012E12	MGC02632	without similarity		-0.10	0.36	0.70	0.36	1.28
Myt01-007H05	MGC00183	translationally controlled tumor protein (TCTP) [Mytilus galloprovincialis]	cell cycle & apoptosis	0.34	0.35	0.69	0.35	1.28
Myt01-001D03	MGC04135	ribosomal protein S26 [Ornithodoros parkeri]	translation	0.30	0.37	0.35	0.35	1.28
Myt01-006B07	MGC01793	eukaryotic translation initiation factor 3, EIF3A protein [Homo sapiens]	translation	0.42	0.35	0.29	0.35	1.28
Myt01-012F04	MGC01598	ribosomal protein rpl6 [Glycera tridactyla]	translation	0.35	0.23	0.63	0.35	1.28
Myt01-002B09	MGC01449	Rab6 subfamily protein [Amblyomma variegatum]	protein folding, turnover & degradation	0.15	0.35	0.37	0.35	1.27
Myt01-015C12	MGC00409	putative C1q domain containing protein MgC1q4 [Mytilus galloprovincialis]	immunity & inflammation	0.50	0.34	0.03	0.34	1.27
Myt01-006F07	MGC01822	without similarity		0.11	0.50	0.34	0.34	1.27
Myt01-016H09	MGC03175	without similarity		0.21	0.34	0.38	0.34	1.26
Myt01-014H03	MGC02933	Epsin-1 [Cricetulus griseus]	protein folding, turnover & degradation	0.33	0.37	0.23	0.33	1.26
Myt01-012B08	MGC02496	ribosomal protein L41 [Mus musculus]	translation	0.08	0.33	0.59	0.33	1.26
Myt01-001C07	MGC00083	ribosomal protein L19 [Argopecten irradians]	translation	0.33	0.14	0.39	0.33	1.26
Myt01-005A03	MGC00648	cytochrome c [Mytilus edulis]	metabolism & ion homeostasis	0.38	0.02	0.33	0.33	1.26
Myt01-009D05	MGC02167	26S proteasome non-ATPase regulatory subunit 8 [Osmerus mordax]	protein folding, turnover & degradation	0.33	0.41	0.32	0.33	1.26
Myt01-006E07	MGC07232	thioredoxin [Ruditapes philippinarum]	metabolism & ion homeostasis	0.34	0.33	0.17	0.33	1.25
Myt01-013D09	MGC02360	peptidase 26S subunit, ATPase 5, isoform CRA_e [Rattus norvegicus]	protein folding, turnover & degradation	0.54	0.33	0.15	0.33	1.25

Myt01-012G02	MGC00047	elongation factor-2 [Pseudopleuronectes americanus]	translation	0.32	0.15	0.68	0.32	1.25
Myt01-016F07	MGC03150	proteasome subunit beta type-7 precursor [Esox lucius]	protein folding, turnover & degradation	0.91	0.32	0.31	0.32	1.25
Myt01-015E08	MGC03040	without similarity		0.32	0.38	0.20	0.32	1.25
Myt01-002B04	MGC01428	hypothetical protein XP_795472 [Strongylocentrotus purpuratus]		0.09	0.49	0.32	0.32	1.25
Myt01-016C10	MGC03127	vdg3 [Mytilus edulis]	development & reproduction	0.32	1.20	0.24	0.32	1.25
Myt01-014H08	MGC02940	without similarity		0.83	0.01	0.31	0.31	1.24
Myt01-012E03	MGC02620	collagen alpha-2(IV) chain [Ascaris suum]	cell adhesion & extracellular matrix	0.60	0.31	0.06	0.31	1.24
Myt01-013G06	MGC00556	60S ribosomal protein L40A [Latrodectus hesperus]	translation	0.42	0.13	0.31	0.31	1.24
Myt01-010C01	MGC00016	40S ribosomal protein S6 [Aplysia californica]	translation	0.29	0.40	0.30	0.30	1.23
Myt01-005H06	MGC00796	ribosomal protein rps15 [Lineus viridis]	translation	0.30	0.19	0.50	0.30	1.23
Myt01-015B10	MGC02984	nucleolar and coiled-body phosphoprotein 1; nucleolar protein p130; [Homo sa	cell cycle & apoptosis	0.03	0.67	0.30	0.30	1.23
Myt01-007A08	MGC00146	ribosomal protein L8 [Chlamys farreri]	translation	0.12	0.36	0.30	0.30	1.23
Myt01-007E01	MGC01899	heat shock cognate 71 [Mytilus galloprovincialis]	protein folding, turnover & degradation	0.84	0.30	0.20	0.30	1.23
Myt01-008E05	MGC02065	40S ribosomal protein S4 [Mytilus edulis]	translation	0.27	0.30	0.53	0.30	1.23
Myt01-007D05	MGC01888	without similarity		0.15	0.29	0.33	0.29	1.22
Myt01-009H10	MGC02193	proteasome 26S subunit-like [Ictalurus punctatus]	protein folding, turnover & degradation	0.29	0.37	0.19	0.29	1.22
Myt01-014A05	MGC00753	ribosomal protein S7 [Argopecten irradians]	translation	0.41	0.29	0.27	0.29	1.22
Myt01-002C12	MGC01468	membrane magnesium transporter 1 precursor [Danio rerio]	signal transduction	0.29	0.51	0.12	0.29	1.22
Myt01-004B10	MGC01645	without similarity		0.51	0.01	0.29	0.29	1.22
Myt01-003C05	MGC01558	myc homolog [Crassostrea virginica]	signal transduction	0.11	0.29	0.37	0.29	1.22
Myt01-001B09	MGC00088	ribosomal protein L28 [Lepidochitona cinerea]	translation	0.29	0.09	0.37	0.29	1.22
Myt01-004C09	MGC00116	without similarity		0.05	0.70	0.29	0.29	1.22
Myt01-017A10	MGC00888	ribosomal protein L26 [Argopecten irradians]	translation	0.09	0.28	0.34	0.28	1.22
Myt01-002H06	MGC01518	without similarity		0.35	0.28	0.13	0.28	1.22
Myt01-015C04	MGC02996	without similarity		0.14	0.40	0.28	0.28	1.21
Myt01-011F10	MGC02493	caspase 3/7-3 [Mytilus galloprovincialis]	cell cycle & apoptosis	0.79	0.28	0.15	0.28	1.21
Myt01-005G09	MGC01186	without similarity		0.87	0.05	0.28	0.28	1.21
Myt01-012D06	MGC02604	Nup205 protein, partial [Mus musculus]	signal transduction	0.28	0.50	0.13	0.28	1.21
Myt01-018A06	MGC01843	RNA-binding protein [Pinctada fucata]	replication, transcription & repair	0.16	0.48	0.28	0.28	1.21
Myt01-005G02	MGC00007	without similarity		0.12	0.77	0.27	0.27	1.21
Myt01-018C11	MGC03382	ribosomal protein L12e [Biphyllus lunatus]	translation	0.23	0.27	0.46	0.27	1.21
Myt01-016H11	MGC00184	40S ribosomal protein S15a [Pectinaria gouldii]	translation	0.27	0.07	0.68	0.27	1.21
Myt01-002G05	MGC01505	without similarity		0.27	0.58	0.03	0.27	1.21
Myt01-006D07	MGC01807	translin-like [Strongylocentrotus purpuratus]	replication, transcription & repair	0.11	0.32	0.27	0.27	1.20
Myt01-005F12	MGC02425	without similarity		0.14	0.50	0.27	0.27	1.20
Myt01-010H05	MGC02377	translationally-controlled tumor protein (TCTP) [Mytilus galloprovincialis]	cell cycle & apoptosis	0.25	0.28	0.27	0.27	1.20
Myt01-008E06	MGC00482	ribosomal protein rps2 [Lineus viridis]	translation	0.20	0.26	0.42	0.26	1.20
Myt01-001E02	MGC01380	without similarity		0.22	0.66	0.26	0.26	1.20
Myt01-013B07	MGC02702	importin-7-like [Strongylocentrotus purpuratus]	signal transduction	0.26	0.52	0.25	0.26	1.20
Myt01-012D07	MGC02605	hypothetical protein DAPPUDRAFT_104151 [Daphnia pulex]		0.39	0.26	0.05	0.26	1.19
Myt01-007D02	MGC07760	proteasome non-ATPase regulatory subunit, partial [Schistocerca gregaria]	protein folding, turnover & degradation	0.25	0.50	0.16	0.25	1.19
Myt01-003B12	MGC01551	without similarity		0.32	0.16	0.25	0.25	1.19
Myt01-004D09	MGC01664	without similarity		0.21	0.25	0.26	0.25	1.19

Myt01-010E11	MGC00146	ribosomal protein L8e [Haliotis midae]	translation	0.19	0.34	0.25	0.25	1.19
Myt01-004H11	MGC01715	without similarity		0.33	0.25	0.07	0.25	1.19
Myt01-009A09	MGC02127	60S ribosomal protein L18 [Aplysia californica]	translation	0.28	0.20	0.25	0.25	1.19
Myt01-012B10	MGC02567	BAT2 domain containing 1-like [Saccoglossus kowalevskii]	cell cycle & apoptosis	0.10	0.25	0.32	0.25	1.19
Myt01-007H10	MGC01969	receptor for activated C-kinase [Pinctada fucata]	signal transduction	-0.02	0.65	0.25	0.25	1.19
Myt01-003E08	MGC01515	small nuclear ribonucleoprotein polypeptide G protein [Crassostrea ariakensis	translation	0.13	0.63	0.25	0.25	1.19
Myt01-011B09	MGC02426	tRNA-dihydrouridine(47) synthase [NAD(P)(+)]-like [Xenopus laevis]	translation	0.36	0.18	0.25	0.25	1.19
Myt01-006D02	MGC01768	without similarity		0.24	0.22	0.51	0.24	1.19
Myt01-007F01	MGC01915	without similarity		0.24	0.59	0.24	0.24	1.18
Myt01-012F05	MGC02639	without similarity		0.13	0.24	0.33	0.24	1.18
Myt01-010C05	MGC02284	ribosomal protein [Mytilus galloprovincialis]	translation	0.24	0.23	0.35	0.24	1.18
Myt01-005A09	MGC06703	without similarity		0.16	0.24	0.54	0.24	1.18
Myt01-010C07	MGC02287	prohibitin-like [Oreochromis niloticus]	replication, transcription & repair	0.42	0.22	0.24	0.24	1.18
Myt01-001A10	MGC00191	elongation factor 1 delta 2 [Xenopus laevis]	translation	0.23	0.22	0.53	0.23	1.18
Myt01-001B08	MGC01348	without similarity		0.19	0.41	0.23	0.23	1.18
Myt01-001G10	MGC00308	ribosomal protein S16 [Lepidochitona cinerea]	translation	0.23	0.19	0.38	0.23	1.17
Myt01-003G10	MGC01611	collagen alpha-5(IV) chain [Rattus norvegicus]	cell adhesion & extracellular matrix	0.23	0.09	0.37	0.23	1.17
Myt01-005C09	MGC05413	without similarity		0.23	0.32	0.14	0.23	1.17
Myt01-018B12	MGC03371	foot protein 3 variant 8 [Mytilus californianus]	cell motility & intracellular trafficking	0.10	0.22	0.51	0.22	1.17
Myt01-014A06	MGC02785	endothelial differentiation-related factor 1-like [Strongylocentrotus purpuratus]	replication, transcription & repair	0.22	0.38	0.20	0.22	1.17
Myt01-001C02	MGC01354	small ribonucleoprotein particle protein SmD3 [Papilio xuthus]	translation	0.18	0.77	0.22	0.22	1.16
Myt01-016E03	MGC00094	without similarity		0.22	0.30	0.19	0.22	1.16
Myt01-012B01	MGC00653	without similarity		0.43	0.21	0.20	0.21	1.16
Myt01-005E10	MGC00002	vdg3 [Mytilus edulis]	development & reproduction	0.20	0.55	0.17	0.20	1.15
Myt01-011G10	MGC00136	ribosomal protein S8 [Crassostrea gigas]	translation	0.06	0.20	0.53	0.20	1.15
Myt01-012G04	MGC02654	microsomal glutathione S-transferase 3 [Pinctada martensi]	metabolism & ion homeostasis	0.89	0.10	0.20	0.20	1.15
Myt01-012B12	MGC02574	electron transfer flavoprotein subunit alpha, mitochondrial-like [Megachile rotu	metabolism & ion homeostasis	0.48	0.10	0.20	0.20	1.15
Myt01-012A11	MGC00007	without similarity		0.00	0.95	0.19	0.19	1.14
Myt01-002G09	MGC01508	without similarity		0.64	0.08	0.18	0.18	1.13
Myt01-001A11	MGC01334	intermediate filament protein A [Aplysia kurodai]	cell motility & intracellular trafficking	0.18	0.15	0.76	0.18	1.13
Myt01-002D08	MGC01474	heterogeneous nuclear ribonucleoprotein K [Homo sapiens]	translation	0.14	0.40	0.18	0.18	1.13
Myt01-002H04	MGC00070	ependymin related protein-1 precursor [Haliotis discus discus]	cell adhesion & extracellular matrix	0.18	0.74	0.02	0.18	1.13
Myt01-012G03	MGC02513	without similarity		-0.05	0.93	0.18	0.18	1.13
Myt01-007F02	MGC01916	cold shock domain containing E1, RNA binding-like [Saccoglossus kowalevsk	replication, transcription & repair	0.17	0.57	0.10	0.17	1.13
Myt01-004C08	MGC00116	without similarity		0.15	0.65	0.09	0.15	1.11
Myt01-007G05	MGC01939	without similarity		0.57	0.14	0.12	0.14	1.10
Myt01-003A02	MGC01526	leucyl-tRNA synthetase [Danio rerio]	translation	0.14	0.69	0.09	0.14	1.10
Myt01-011D10	MGC00399	ribosomal protein L7 [Argopecten irradians]	translation	0.09	0.10	0.82	0.10	1.07
**Under-expressed**								
Myt01-007E11	MGC01313	cytochrome c oxidase subunit IV [Mytilus edulis]	metabolism & ion homeostasis	-0.13	-0.92	-0.14	-0.14	1.11
Myt01-003B11	MGC00160	hemagglutinin/amebocyte aggregation factor precursor [Mytilus edulis]	immunity & inflammation	-0.15	-0.61	-0.16	-0.16	1.11
Myt01-017C08	MGC09187	without similarity		-0.84	-0.05	-0.18	-0.18	1.14
Myt01-004A06	MGC01630	putative binding precursor [Mytilus edulis]	cell adhesion & extracellular matrix	-0.19	-1.09	-0.10	-0.19	1.14

Myt01-012H05	MGC02667	without similarity		-0.61	-0.11	-0.20	-0.20	1.15
Myt01-018A04	MGC03266	without similarity		-0.20	-0.39	-0.19	-0.20	1.15
Myt01-012C12	MGC02597	hypothetical protein NEMVEDRAFT_v1g2616 [Nematostella vectensis]		-0.20	-0.16	-0.63	-0.20	1.15
Myt01-016D09	MGC00176	alpha tubulin [Pectinaria gouldii]	cell motility & intracellular trafficking	-0.22	-0.65	-0.09	-0.22	1.16
Myt01-004B11	MGC01646	unknown [Dendroctonus ponderosae]		-0.55	-0.10	-0.23	-0.23	1.17
Myt01-002C05	MGC00406	histone variant H2A.Z [Mytilus galloprovincialis]	replication, transcription & repair	-0.24	-0.55	-0.21	-0.24	1.18
Myt01-005H12	MGC00440	hypothetical protein CHLREDRAFT_181491 [Chlamydomonas reinhardtii]		-0.12	-0.52	-0.25	-0.25	1.19
Myt01-015E01	MGC03023	mitochondrial ATPase alpha subunit [Ictalurus punctatus]	metabolism & ion homeostasis	-0.50	-0.26	-0.07	-0.26	1.19
Myt01-007B10	MGC00473	without similarity		-0.17	-1.09	-0.26	-0.26	1.19
Myt01-009E02	MGC01706	ubiquinol-cytochrome c reductase subunit 6 [Mytilus edulis]	metabolism & ion homeostasis	-0.26	-0.23	-0.29	-0.26	1.19
Myt01-002C06	MGC01459	protein-glutamine gamma-glutamyltransferase 4-like [Anolis carolinensis]	metabolism & ion homeostasis	-0.26	-0.32	-0.20	-0.26	1.19
Myt01-017A03	MGC03179	without similarity		-0.26	-0.42	-0.06	-0.26	1.20
Myt01-014C04	MGC02851	ribosomal protein L4-like [Saccoglossus kowalevskii]	translation	-0.25	-0.26	-0.28	-0.26	1.20
Myt01-006F12	MGC01828	without similarity		-0.03	-0.55	-0.26	-0.26	1.20
Myt01-014E11	MGC02898	pedal retractor muscle myosin heavy chain [Mytilus galloprovincialis]	cell motility & intracellular trafficking	-0.27	-0.36	-0.22	-0.27	1.20
Myt01-003F11	MGC01599	without similarity		-0.27	-0.14	-0.84	-0.27	1.21
Myt01-017F04	MGC01209	ribosomal protein S24; MRP S24 [Mus musculus]	translation	-0.22	-0.46	-0.28	-0.28	1.21
Myt01-006F08	MGC01824	involved in global regulation of transcription; Snf5p [Saccharomyces cerevisia	replication, transcription & repair	-0.19	-0.28	-0.31	-0.28	1.21
Myt01-016E07	MGC03139	beta-1,4-endoglucanase 2 [Panesthia cribrata]	metabolism & ion homeostasis	-0.44	-0.28	-0.27	-0.28	1.21
Myt01-015E06	MGC00387	ATP synthase subunit beta, mitochondrial-like [Bombus impatiens]	metabolism & ion homeostasis	-0.28	-0.51	-0.27	-0.28	1.21
Myt01-006C12	MGC04193	RWD domain-containing protein 1-like [Bombus impatiens]		-0.28	-0.12	-0.52	-0.28	1.21
Myt01-012A02	MGC02531	without similarity		-0.87	-0.23	-0.28	-0.28	1.22
Myt01-012D12	MGC00318	beta-actin, partial [Catla catla]	cell motility & intracellular trafficking	-0.28	-0.24	-0.61	-0.28	1.22
Myt01-014B12	MGC01827	hypothetical protein BRAFLDRAFT_86469 [Branchiostoma floridae]		-0.29	-0.84	-0.08	-0.29	1.22
Myt01-007H03	MGC01960	polyphenolic adhesive protein [Mytilus edulis]	cell adhesion & extracellular matrix	-0.70	-0.29	-0.21	-0.29	1.22
Myt01-001H09	MGC01426	without similarity		-0.29	-0.48	-0.28	-0.29	1.22
Myt01-016C12	MGC00089	without similarity		-0.41	-0.24	-0.29	-0.29	1.22
Myt01-012A08	MGC00931	mytimacin-2 [Mytilus galloprovincialis]	immunity & inflammation	-0.29	-0.34	-0.19	-0.29	1.22
Myt01-001D11	MGC01377	tetratricopeptide repeat protein 25-like [Danio rerio]		-0.29	-0.18	-0.38	-0.29	1.23
Myt01-015B04	MGC02977	without similarity		-0.30	-0.41	-0.17	-0.30	1.23
Myt01-016D10	MGC03133	without similarity		-0.30	-0.28	-0.35	-0.30	1.23
Myt01-005H07	MGC01780	without similarity		-0.21	-0.30	-0.33	-0.30	1.23
Myt01-018G11	MGC00860	Integumentary mucin C.1 (FIM-C.1) [Xenopus laevis]	cell adhesion & extracellular matrix	-0.24	-0.40	-0.30	-0.30	1.23
Myt01-014H06	MGC02938	without similarity		-0.45	-0.28	-0.30	-0.30	1.23
Myt01-017C07	MGC03205	without similarity		-0.53	-0.03	-0.30	-0.30	1.23
Myt01-008G11	MGC02102	without similarity		-0.30	-0.15	-0.33	-0.30	1.23
Myt01-006H12	MGC00243	alpha-tubulin, partial [Nodipecten subnodosus]	cell motility & intracellular trafficking	-0.30	-0.90	-0.30	-0.30	1.23
Myt01-003F09	MGC01596	precollagen-P [Mytilus galloprovincialis]	cell adhesion & extracellular matrix	-0.31	-0.19	-0.36	-0.31	1.24
Myt01-018E07	MGC03419	C1q domain containing protein MgC1q48 [Mytilus galloprovincialis]	immunity & inflammation	-0.15	-0.43	-0.31	-0.31	1.24
Myt01-012C01	MGC02576	without similarity		-0.25	-0.40	-0.31	-0.31	1.24
Myt01-016D06	MGC00161	apolipophorin precursor [Locusta migratoria]	metabolism & ion homeostasis	-0.32	-0.46	-0.25	-0.32	1.25
Myt01-010D01	MGC02298	without similarity		-0.62	-0.18	-0.32	-0.32	1.25
Myt01-006D12	MGC02425	without similarity		-0.07	-0.51	-0.32	-0.32	1.25

Myt01-013B03	MGC02698	without similarity		-0.34	-0.32	-0.24	-0.32	1.25
Myt01-016A01	MGC03116	proliferating cell nuclear antigen [Litopenaeus vannamei]	replication, transcription & repair	-0.20	-0.40	-0.33	-0.33	1.26
Myt01-015F12	MGC01896	without similarity		-0.33	-0.47	-0.24	-0.33	1.26
Myt01-006C01	MGC07381	without similarity		-0.33	-0.29	-0.56	-0.33	1.26
Myt01-001A04	MGC01325	PACRG, partial [Xenopus laevis]	protein folding, turnover & degradation	-0.30	-0.48	-0.33	-0.33	1.26
Myt01-017F02	MGC01802	collagen alpha-1(XII) chain-like [Anolis carolinensis]	cell adhesion & extracellular matrix	-0.34	-0.38	-0.24	-0.34	1.26
Myt01-017G06	MGC03000	without similarity		-0.25	-0.35	-0.55	-0.35	1.27
Myt01-010H11	MGC02389	without similarity		-1.10	-0.35	-0.32	-0.35	1.27
Myt01-001E01	MGC01379	without similarity		-0.08	-0.36	-0.35	-0.35	1.28
Myt01-002B03	MGC01443	neurofilament protein NF70 [Helix aspersa]	cell motility & intracellular trafficking	-0.59	0.00	-0.36	-0.36	1.29
Myt01-017E11	MGC03246	DEAD (Asp-Glu-Ala-Asp) box polypeptide 42 [Danio rerio]	replication, transcription & repair	-0.46	-0.36	-0.28	-0.36	1.29
Myt01-014G10	MGC00910	steroid dehydrogenase [Schistosoma mansoni]	metabolism & ion homeostasis	-0.41	0.01	-0.37	-0.37	1.29
Myt01-003E06	MGC01585	without similarity		-0.39	-0.37	-0.22	-0.37	1.29
Myt01-011E11	MGC00081	without similarity		-0.38	-0.25	-0.37	-0.37	1.29
Myt01-013H01	MGC00821	without similarity		-0.34	-0.68	-0.37	-0.37	1.30
Myt01-016B08	MGC01743	endo-1,3-beta-D-glucanase [Perna viridis]	metabolism & ion homeostasis	-0.38	-0.43	-0.33	-0.38	1.30
Myt01-019B12	MGC10002	cytochrome b [Mytilus galloprovincialis]	metabolism & ion homeostasis	-0.22	-0.38	-0.70	-0.38	1.30
Myt01-006G09	MGC01835	macrophage expressed protein [Crassostrea gigas]	immunity & inflammation	-0.84	-0.30	-0.38	-0.38	1.30
Myt01-007H09	MGC05903	without similarity		-0.38	-1.13	-0.08	-0.38	1.30
Myt01-016D08	MGC03131	without similarity		-0.49	-0.39	-0.31	-0.39	1.31
Myt01-002E04	MGC00414	without similarity		-0.39	-0.86	-0.12	-0.39	1.31
Myt01-006E10	MGC00915	without similarity		-0.39	0.02	-1.02	-0.39	1.31
Myt01-010A03	MGC10011	cytochrome b [Mytilus galloprovincialis]	metabolism & ion homeostasis	-0.23	-0.45	-0.39	-0.39	1.31
Myt01-013G09	MGC02798	without similarity		-0.69	-0.34	-0.40	-0.40	1.32
Myt01-001E06	MGC01384	without similarity		-0.41	-0.40	-0.13	-0.40	1.32
Myt01-013B02	MGC02697	hypothetical protein BRAFLDRAFT_74509 [Branchiostoma floridae]		-0.40	-0.80	0.14	-0.40	1.32
Myt01-017G09	MGC03000	without similarity		-0.08	-0.62	-0.40	-0.40	1.32
Myt01-002B05	MGC01445	without similarity		-0.96	0.18	-0.41	-0.41	1.33
Myt01-016A05	MGC00125	fibrinogen-related protein 7 [Mytilus galloprovincialis]	immunity & inflammation	-0.36	-0.53	-0.41	-0.41	1.33
Myt01-006A03	MGC01810	without similarity		-0.66	-0.19	-0.41	-0.41	1.33
Myt01-017D06	MGC08512	without similarity		-0.55	-0.34	-0.41	-0.41	1.33
Myt01-013D08	MGC02747	without similarity		-0.41	-0.10	-1.05	-0.41	1.33
Myt01-006A11	MGC02779	precollagen-NG mRNA, complete cds [Mytilus galloprovincialis]	cell adhesion & extracellular matrix	-0.41	-0.83	-0.38	-0.41	1.33
Myt01-001F02	MGC01391	hypothetical protein BRAFLDRAFT_118794 [Branchiostoma floridae]		-0.42	-0.22	-0.54	-0.42	1.33
Myt01-019B11	MGC10002	cytochrome c oxidase subunit II [Mytilus galloprovincialis]	metabolism & ion homeostasis	-0.33	-0.42	-0.44	-0.42	1.34
Myt01-002G08	MGC00618	proteasome (prosome, macropain) subunit, beta type, 1, isoform CRA_c [Hon	protein folding, turnover & degradation	-0.45	-0.16	-0.42	-0.42	1.34
Myt01-002H12	MGC01524	leucine--tRNA ligase, cytoplasmic-like [Strongylocentrotus purpuratus]	translation	-0.97	-0.06	-0.43	-0.43	1.34
Myt01-015G12	MGC02989	precollagen-D [Mytilus galloprovincialis]	cell adhesion & extracellular matrix	-0.63	-0.43	-0.21	-0.43	1.35
Myt01-003H01	MGC01614	calmodulin [Porphyra yezoensis]	cell adhesion & extracellular matrix	-0.43	-0.80	-0.32	-0.43	1.35
Myt01-011E07	MGC02476	stress-associated endoplasmic reticulum protein 2 [Homo sapiens]	protein folding, turnover & degradation	-0.43	-0.45	-0.39	-0.43	1.35
Myt01-015F09	MGC03058	protein kinase C-binding protein NELL1, putative [Ixodes scapularis]	signal transduction	-0.06	-0.43	-0.52	-0.43	1.35
Myt01-014A10	MGC02825	asialoglycoprotein receptor 2 [Danio rerio]	signal transduction	-0.63	-0.43	-0.10	-0.43	1.35
Myt01-010H04	MGC00490	without similarity		-0.44	-0.62	-0.14	-0.44	1.35

Myt01-016C01	MGC00114	without similarity		-0.44	-0.40	-0.49	-0.44	1.35
Myt01-001G04	MGC01408	arginine kinase [Sepiella maindroni]	metabolism & ion homeostasis	-0.44	-0.85	-0.29	-0.44	1.36
Myt01-016F06	MGC03149	without similarity		-0.26	-0.55	-0.45	-0.45	1.36
Myt01-012B04	MGC02555	kyphoscoliosis peptidase [Clonorchis sinensis]	protein folding, turnover & degradation	-0.45	-0.51	-0.36	-0.45	1.36
Myt01-014E01	MGC02881	without similarity		-0.01	-0.45	-0.50	-0.45	1.37
Myt01-002E12	MGC01488	without similarity		-0.45	-0.67	0.10	-0.45	1.37
Myt01-007F09	MGC01920	DEAD (Asp-Glu-Ala-Asp) box polypeptide 17 [Taeniopygia guttata]	replication, transcription & repair	-0.45	-0.48	-0.45	-0.45	1.37
Myt01-018G01	MGC00020	without similarity		-0.46	-0.68	-0.39	-0.46	1.37
Myt01-010G08	MGC02370	radial spokehead [Strongylocentrotus purpuratus]	cell motility & intracellular trafficking	-0.37	-0.49	-0.47	-0.47	1.38
Myt01-017F09	MGC03272	without similarity		-0.48	-0.50	-0.24	-0.48	1.40
Myt01-014E06	MGC02887	mytimacin-4 [Mytilus galloprovincialis]	immunity & inflammation	-0.38	-0.48	-0.60	-0.48	1.40
Myt01-004D12	MGC01669	without similarity		-0.18	-0.48	-0.53	-0.48	1.40
Myt01-019B01	MGC01938	without similarity		-0.38	-0.90	-0.49	-0.49	1.40
Myt01-002B12	MGC00417	without similarity		-1.01	-0.49	-0.04	-0.49	1.41
Myt01-017D09	MGC03213	without similarity		-0.49	-0.49	-0.21	-0.49	1.41
Myt01-015B05	MGC02494	without similarity		-0.49	-0.71	-0.36	-0.49	1.41
Myt01-004B07	MGC01642	without similarity		-0.29	-0.51	-0.63	-0.51	1.42
Myt01-015E05	MGC03031	lethal(2) giant larvae protein homolog 1 [Ornithorhynchus anatinus]	development & reproduction	-0.24	-0.64	-0.51	-0.51	1.43
Myt01-019B04	MGC10003	ATP synthase F0 subunit 6 [Mytilus galloprovincialis]	metabolism & ion homeostasis	-0.14	-0.52	-0.52	-0.52	1.43
Myt01-006G10	MGC10001	cytochrome oxidase subunit I [Mytilus galloprovincialis]	metabolism & ion homeostasis	-0.28	-0.55	-0.52	-0.52	1.44
Myt01-016D05	MGC00167	thymosin beta-4-like [Oreochromis niloticus]	cell motility & intracellular trafficking	-0.53	-0.69	-0.09	-0.53	1.44
Myt01-016C11	MGC03129	without similarity		-0.50	-0.61	-0.53	-0.53	1.45
Myt01-006E12	MGC04318	predicted protein-like [Saccoglossus kowalevskii]		-0.03	-0.80	-0.54	-0.54	1.45
Myt01-003B05	MGC01540	uncharacterized protein C5orf48 homolog [Strongylocentrotus purpuratus]		-0.61	-0.53	-0.54	-0.54	1.46
Myt01-018H06	MGC03479	multiple C2 and transmembrane domain-containing protein 1 [Strongylocentrotus purpuratus]		-0.40	-0.63	-0.54	-0.54	1.46
Myt01-017C11	MGC03207	without similarity		-0.24	-0.55	-0.56	-0.55	1.46
Myt01-017H10	MGC03327	without similarity		-0.78	-0.55	-0.42	-0.55	1.46
Myt01-013C05	MGC02449	nongradient byssal precursor [Mytilus edulis]	cell motility & intracellular trafficking	-0.55	-0.68	-0.28	-0.55	1.47
Myt01-016E11	MGC03142	without similarity		-0.50	-0.56	-0.63	-0.56	1.47
Myt01-006A09	MGC01789	without similarity		-0.82	-0.26	-0.56	-0.56	1.47
Myt01-017C05	MGC02472	DC2-related axonemal dynein intermediate chain 4 [Ciona intestinalis]	cell motility & intracellular trafficking an	-0.40	-0.68	-0.56	-0.56	1.47
Myt01-015C02	MGC01307	alpha tubulin [Pinctada fucata]	cell motility & intracellular trafficking	-0.33	-0.82	-0.56	-0.56	1.48
Myt01-003G05	MGC01606	universal stress protein MSMEG_3950 [Clonorchis sinensis]		-0.56	-0.65	-0.39	-0.56	1.48
Myt01-004D04	MGC00014	defensin-like protein 298 [Arabidopsis thaliana]	immunity & inflammation	-0.17	-0.69	-0.57	-0.57	1.48
Myt01-003D04	MGC01543	without similarity		-0.24	-0.64	-0.57	-0.57	1.49
Myt01-019B06	MGC10001	cytochrome c oxidase subunit I [Mytilus californianus]	metabolism & ion homeostasis	-0.57	-0.28	-0.79	-0.57	1.49
Myt01-003H07	MGC01296	without similarity		-0.65	-0.58	-0.01	-0.58	1.49
Myt01-018F04	MGC03442	hypothetical protein DDB_G0287291 [Dictyostelium discoideum AX4]		-0.55	-0.72	-0.58	-0.58	1.49
Myt01-015A11	MGC00019	6-phosphogluconolactonase-like [Strongylocentrotus purpuratus]	metabolism & ion homeostasis	-0.45	-0.58	-0.63	-0.58	1.50
Myt01-008B01	MGC01999	without similarity		0.02	-0.59	-1.07	-0.59	1.51
Myt01-017F12	MGC07381	without similarity		-0.67	-0.60	-0.32	-0.60	1.51
Myt01-011C11	MGC02439	Q/N-rich domain Prion like protein PQN-75 (pqn-75) [Caenorhabditis elegans]		-0.05	-0.60	-0.94	-0.60	1.51
Myt01-014G12	MGC02929	without similarity		-0.42	-0.61	-0.77	-0.61	1.52

Myt01-015H10	MGC03114	C1q domain containing protein MgC1q89 [Mytilus galloprovincialis]	immunity & inflammation	-0.77	-0.61	-0.32	-0.61	1.52
Myt01-017E02	MGC03224	neuron-specific staufen [Aplysia californica]		-0.62	-0.42	-0.76	-0.62	1.53
Myt01-016A12	MGC00159	without similarity		-0.62	-0.39	-0.63	-0.62	1.53
Myt01-009G02	MGC02210	histone deacetylation protein Rxt3 [Glomerella graminicola M1.001]	replication, transcription & repair	-0.63	-0.78	-0.51	-0.63	1.55
Myt01-018F07	MGC03446	tubulin polymerization-promoting protein family member 2-like [Cavia porcellus	cell motility & intracellular trafficking	-0.64	-0.84	-0.34	-0.64	1.56
Myt01-015H12	MGC00110	actin [Mizuhopecten yessoensis]	cell motility & intracellular trafficking	-0.37	-0.72	-0.64	-0.64	1.56
Myt01-006G12	MGC01838	without similarity		-0.24	-0.65	-0.81	-0.65	1.57
Myt01-014F11	MGC02911	fringe [Euprymna scolopes]	signal transduction	-0.41	-1.00	-0.66	-0.66	1.58
Myt01-011H07	MGC02521	axonemal dynein light chain p33 [Haliotis discus discus]	cell motility & intracellular trafficking	-0.66	-1.09	-0.32	-0.66	1.58
Myt01-016A08	MGC00131	zona pellucida domain protein D [Haliotis rufescens]	development & reproduction	-0.66	-0.69	-0.55	-0.66	1.58
Myt01-005F09	MGC10006	mitochondrial NADH dehydrogenase subunit 5 (ND5) and 6 (ND6) and cyt b [	metabolism & ion homeostasis	-0.83	-0.59	-0.68	-0.68	1.60
Myt01-014F09	MGC02906	CG10903-PA [Strongylocentrotus purpuratus]		-0.51	-0.76	-0.68	-0.68	1.60
Myt01-003G12	MGC01613	INO80 complex subunit C-like [Danio rerio]	replication, transcription & repair	-0.43	-0.87	-0.70	-0.70	1.62
Myt01-011E05	MGC02473	without similarity		-0.72	-0.94	-0.03	-0.72	1.65
Myt01-013C10	MGC01399	without similarity		-0.39	-0.73	-0.90	-0.73	1.66
Myt01-016C09	MGC00117	beta-microseminoprotein [Xenopus (Silurana) tropicalis]	immunity & inflammation	-0.51	-0.75	-0.74	-0.74	1.67
Myt01-014E05	MGC02886	collagen pro alpha-chain [Haliotis discus]	cell adhesion & extracellular matrix	-0.37	-0.89	-0.78	-0.78	1.72
Myt01-004D10	MGC01665	exosome component 2-like [Saccoglossus kowalevskii]	replication, transcription & repair	-0.13	-0.98	-0.81	-0.81	1.75
Myt01-011E03	MGC02470	without similarity		-1.23	-0.25	-0.83	-0.83	1.78
Myt01-014A03	MGC05861	hypothetical protein BRAFLDRAFT_270517 [Branchiostoma floridae]		-0.31	-0.84	-0.84	-0.84	1.78
Myt01-012D04	MGC00206	without similarity		-0.98	-0.87	0.12	-0.87	1.82
Myt01-003E07	MGC00175	beta tubulin [Chlamys farreri]	cell adhesion & extracellular matrix	-0.82	-1.12	-0.87	-0.87	1.83
Myt01-010B10	MGC02276	alpha 1 type XII collagen short isoform precursor [Homo sapiens]	cell adhesion & extracellular matrix	-2.29	0.16	-0.88	-0.88	1.84
Myt01-007D06	MGC00206	without similarity		-1.30	-0.89	0.21	-0.89	1.86
Myt01-019B08	MGC02110	Mitochondrial-ND6	metabolism & ion homeostasis	-0.13	-1.10	-0.95	-0.95	1.93
Myt01-016G11	MGC03164	without similarity		-1.27	-0.96	0.43	-0.96	1.95
Myt01-002H11	MGC01523	without similarity		-1.28	-1.11	-0.38	-1.11	2.16
Myt01-017B09	MGC03196	without similarity		-1.16	-1.12	-0.68	-1.12	2.18
Myt01-007H08	MGC01966	inhibitor of apoptosis 1 [Gallus gallus]	cell cycle & apoptosis	-1.13	-1.83	-0.92	-1.13	2.19
Myt01-002C11	MGC01465	without similarity		-0.13	-2.58	-1.14	-1.14	2.21
Myt01-016D12	MGC00084	without similarity		-2.28	-1.28	-1.21	-1.28	2.43
Myt01-008B03	MGC02005	without similarity		-1.90	-1.07	-1.58	-1.58	3.00
