# Supplementary material for: DNA Damage and Transcriptional Changes in the Gills of Mytilus galloprovincialis Exposed to Nanomolar Doses of Combined Metal Salts (Cd, Cu, Hg)
Source: PLoS One. 2013 Jan 23;8(1):e54602. doi: 10.1371/journal.pone.0054602 (PMC3552849; doi:10.1371/journal.pone.0054602)
Supplement: Table S5 — Identity and expression values of genes differentially expressed in the gills of individual mussels exposed to the combined metals (genes in common between doses, extracted from Tables S2, S3, S4). (PDF) [file pone.0054602.s007.pdf]

**Table S5. Identity and expression values of genes differentially expressed in the gills of individual mussels exposed to the combined metals (genes in common between doses, extracted from Tables S2-S4).**

| | | | | Expression value (log2) | | | | | | | | | | | |
|---|---|---|---|---|---|---|---|---|---|---|---|---|---|---|---|
| | | | | 50 nM | | | | 100 nM | | | | 200 nM | | | |
| | | | | Mussel number: | | | | | | | | | | | |
| **Mytarray 1.0 ID** | **Mytibase ID** | **Description** | **Functional category** | **6** | **7** | **8** | **Median** | **11** | **12** | **13** | **Median** | **16** | **17** | **18** | **Median** |
| Myt01-016G09 | MGC00670 | heat shock protein 90 [Mytilus galloprovincialis] | protein folding, turnover & degradation | 0.64 | 0.51 | 0.03 | **0.51** | -0.09 | 0.61 | 0.85 | **0.61** | 1.28 | 1.28 | 1.19 | **1.28** |
| Myt01-011G05 | MGC00301 | small heat shock protein 24.1 [Mytilus galloprovincialis] | protein folding, turnover & degradation | 0.78 | 0.05 | 0.50 | **0.50** | 0.06 | 1.04 | 0.90 | **0.90** | 2.26 | 1.16 | 1.26 | **1.26** |
| Myt01-015C02 | MGC01307 | alpha tubulin [Pinctada fucata] | cell motility & intracellular trafficking | -0.51 | -0.10 | -0.15 | **-0.15** | -0.32 | -0.55 | -0.66 | **-0.55** | -0.33 | -0.82 | -0.56 | **-0.56** |
| Myt01-003C09 | MGC00100 | eukaryotic translation initiation factor 5A [Branchiostoma belcheri] | translation | 0.50 | 0.53 | 0.00 | **0.50** | 0.72 | 0.42 | 0.86 | **0.72** | | | | |
| Myt01-003A09 | MGC01531 | without similarity | | 0.65 | 0.71 | -0.12 | **0.65** | 0.24 | 0.42 | 0.33 | **0.33** | | | | |
| Myt01-014D10 | MGC01476 | precollagen-P [Mytilus galloprovincialis] | cell adhesion & extracellular matrix | 0.37 | 0.17 | 0.06 | **0.17** | -0.73 | -0.09 | -0.22 | **-0.22** | | | | |
| Myt01-011G08 | MGC02503 | without similarity | | 0.13 | 0.19 | 0.42 | **0.19** | -0.68 | -0.28 | -0.01 | **-0.28** | | | | |
| Myt01-014G05 | MGC00222 | C1q domain containing protein MgC1q98 [Mytilus galloprovincialis] | immunity & inflammation | 0.39 | 0.15 | 0.46 | **0.39** | -0.85 | -0.10 | -0.34 | **-0.34** | | | | |
| Myt01-001C01 | MGC01352 | without similarity | | -0.83 | -0.25 | -0.03 | **-0.25** | -1.19 | -0.61 | -0.21 | **-0.61** | | | | |
| Myt01-005D09 | MGC01749 | poly(A)-binding protein [Spisula solidissima] | translation | 0.44 | 0.60 | 0.16 | **0.44** | | | | | 0.46 | 0.62 | -0.10 | **0.46** |
| Myt01-005H12 | MGC00440 | hypothetical protein CHLREDRAFT_181491 [Chlamydomonas reinhardtii] | | 0.93 | -0.02 | 0.32 | **0.32** | | | | | -0.12 | -0.52 | -0.25 | **-0.25** |
| Myt01-012D05 | MGC02603 | without similarity | | 0.53 | 0.24 | 0.18 | **0.24** | | | | | 0.65 | 0.18 | 0.62 | **0.62** |
| Myt01-012B10 | MGC02567 | BAT2 domain containing 1-like [Saccoglossus kowalevskii] | cell cycle & apoptosis | 0.41 | 0.21 | 0.21 | **0.21** | | | | | 0.10 | 0.25 | 0.32 | **0.25** |
| Myt01-019B05 | MGC10007 | NADH dehydrogenase subunit 1 [Mytilus edulis] | metabolism & ion homeostasis | -0.19 | -0.19 | -0.54 | **-0.19** | | | | | 0.02 | 0.40 | 0.51 | **0.40** |
| Myt01-010A03 | MGC10011 | cytochrome b [Mytilus galloprovincialis] | metabolism & ion homeostasis | -0.40 | -0.23 | -0.24 | **-0.24** | | | | | -0.23 | -0.45 | -0.39 | **-0.39** |
| Myt01-015B09 | MGC02960 | incilarin A [Haliotis discus discus] | signal transduction | -0.67 | -0.15 | -0.86 | **-0.67** | | | | | 1.14 | 1.23 | -0.20 | **1.14** |
| Myt01-007D04 | MGC00749 | sequestosome-1 [Harpegnathos saltator] | protein folding, turnover & degradation | | | | | 2.65 | 4.15 | 3.20 | **3.20** | 5.19 | 4.28 | 3.77 | **4.28** |
| Myt01-012A04 | MGC02534 | FK506-binding protein [Suberites domuncula] | protein folding, turnover & degradation | | | | | 1.24 | 1.08 | 0.88 | **1.08** | 1.03 | 1.01 | 0.90 | **1.01** |
| Myt01-014C10 | MGC02858 | without similarity | | | | | | 0.50 | 1.20 | 1.06 | **1.06** | 1.19 | 1.73 | 1.81 | **1.73** |
| Myt01-010C12 | MGC02297 | glutathione S-transferase GSTpi1 [Mytilus galloprovincialis] | metabolism & ion homeostasis | | | | | 0.04 | 1.05 | 1.14 | **1.05** | 1.95 | 1.77 | 1.11 | **1.77** |
| Myt01-013C12 | MGC02733 | small heat shock protein 24.1 [Mytilus galloprovincialis] | protein folding, turnover & degradation | | | | | -0.01 | 1.33 | 1.00 | **1.00** | 1.70 | 1.03 | 0.57 | **1.03** |
| Myt01-005F03 | MGC02733 | without similarity | | | | | | 0.53 | 1.06 | 0.94 | **0.94** | 1.81 | 1.11 | 0.89 | **1.11** |
| Myt01-011H08 | MGC02331 | precollagen-D [Mytilus galloprovincialis] | cell adhesion & extracellular matrix | | | | | -0.01 | 0.85 | 1.10 | **0.85** | 0.32 | 0.90 | 0.71 | **0.71** |
| Myt01-014B11 | MGC01310 | heat shock protein 70 [Mytilus galloprovincialis] | protein folding, turnover & degradation | | | | | 1.10 | 0.85 | 0.74 | **0.85** | 1.56 | 1.14 | 1.22 | **1.22** |
| Myt01-011E01 | MGC02468 | without similarity | | | | | | 0.62 | 0.76 | 0.97 | **0.76** | 0.41 | 0.46 | 0.77 | **0.46** |
| Myt01-016C08 | MGC01659 | metallothionein-10B [Mytilus galloprovincialis] | metabolism & ion homeostasis | | | | | -0.04 | 0.88 | 0.74 | **0.74** | 2.19 | 0.77 | 1.75 | **1.75** |
| Myt01-012F06 | MGC02640 | taurine transporter [Mytilus galloprovincialis] | signal transduction | | | | | 0.73 | 0.75 | 0.52 | **0.73** | 1.05 | 0.74 | 0.68 | **0.74** |
| Myt01-003E08 | MGC01515 | small nuclear ribonucleoprotein polypeptide G protein [Crassostrea ariakensis] | translation | | | | | 0.66 | 0.74 | 0.42 | **0.66** | 0.13 | 0.63 | 0.25 | **0.25** |
| Myt01-002B06 | MGC00093 | without similarity | | | | | | 0.61 | 0.69 | 0.30 | **0.61** | 0.90 | 0.71 | 0.66 | **0.71** |
| Myt01-001E02 | MGC01380 | without similarity | | | | | | 0.84 | 0.04 | 0.58 | **0.58** | 0.22 | 0.66 | 0.26 | **0.26** |
| Myt01-003G03 | MGC01604 | 26S proteasome non-ATPase regulatory subunit 1 [Camponotus floridanus] | protein folding, turnover & degradation | | | | | 0.59 | 0.08 | 0.56 | **0.56** | 1.18 | 0.69 | 0.21 | **0.69** |
| Myt01-001H12 | MGC01429 | ADP-ribosylation factor 2, isoform CRA_b [Mus musculus] | signal transduction | | | | | 0.07 | 1.27 | 0.52 | **0.52** | 1.10 | 0.65 | 0.41 | **0.65** |
| Myt01-017B08 | MGC03195 | without similarity | | | | | | 0.51 | 0.62 | 0.41 | **0.51** | 1.23 | 0.67 | 0.92 | **0.92** |
| Myt01-011G07 | MGC00126 | without similarity | | | | | | 0.74 | 0.11 | 0.50 | **0.50** | 0.21 | 0.53 | 0.61 | **0.53** |
| Myt01-015E03 | MGC03027 | without similarity | | | | | | 0.39 | 0.48 | 0.48 | **0.48** | 0.56 | 0.11 | 0.89 | **0.56** |
| Myt01-010B03 | MGC02267 | heat shock cognate 70 [Mytilus galloprovincialis] | protein folding, turnover & degradation | | | | | 0.27 | 0.95 | 0.47 | **0.47** | 1.44 | 0.88 | 0.89 | **0.89** |
| Myt01-002A11 | MGC01439 | small nuclear ribonucleoprotein associated protein B [Mustela putorius furo] | translation | | | | | 0.31 | 0.58 | 0.46 | **0.46** | 0.52 | 0.69 | 0.60 | **0.60** |
| Myt01-018H05 | MGC01435 | ribosomal protein S12 [Pinctada maxima] | translation | | | | | 0.59 | 0.11 | 0.44 | **0.44** | 0.15 | 0.52 | 0.71 | **0.52** |
| Myt01-017B01 | MGC03187 | selenide, water dikinase [Harpegnathos saltator] | metabolism & ion homeostasis | | | | | 0.44 | 0.52 | 0.34 | **0.44** | 0.37 | 0.52 | 0.10 | **0.37** |
| Myt01-014B07 | MGC02839 | without similarity | | | | | | 0.29 | 0.70 | 0.44 | **0.44** | 1.35 | 1.38 | 0.30 | **1.35** |
| Myt01-014B06 | MGC02837 | chaperonin subunit 7 [Epinephelus coioides] | protein folding, turnover & degradation | | | | | 0.43 | 0.51 | 0.40 | **0.43** | 0.89 | 0.75 | 0.41 | **0.75** |
| Myt01-018F02 | MGC01507 | ribosomal protein S27E [Mytilus galloprovincialis] | translation | | | | | 0.42 | 0.35 | 0.46 | **0.42** | 0.67 | 0.53 | 0.44 | **0.53** |
| Myt01-012B08 | MGC02496 | ribosomal protein L41 [Mus musculus] | translation | | | | | 0.76 | 0.19 | 0.41 | **0.41** | 0.08 | 0.33 | 0.59 | **0.33** |
| Myt01-007D02 | MGC07760 | proteasome non-ATPase regulatory subunit, partial [Schistocerca gregaria] | protein folding, turnover & degradation | | | | | 0.36 | 0.42 | 0.38 | **0.38** | 0.25 | 0.50 | 0.16 | **0.25** |
| Myt01-002C12 | MGC01468 | membrane magnesium transporter 1 precursor [Danio rerio] | signal transduction | | | | | 0.22 | 0.43 | 0.37 | **0.37** | 0.29 | 0.51 | 0.12 | **0.29** |
| Myt01-004F11 | MGC01693 | without similarity | | | | | | 0.18 | 0.38 | 0.35 | **0.35** | 1.17 | 0.92 | 1.20 | **1.17** |
| Myt01-008E03 | MGC02063 | without similarity | | | | | | 0.40 | 0.35 | 0.26 | **0.35** | 0.58 | 0.46 | 0.10 | **0.46** |
| Myt01-017C08 | MGC09187 | without similarity | | | | | | 0.39 | 0.34 | 0.21 | **0.34** | -0.84 | -0.05 | -0.18 | **-0.18** |
| Myt01-015F10 | MGC00476 | cold shock domain protein [Chlamys farreri] | replication, transcription & repair | | | | | 0.31 | 0.54 | 0.08 | **0.31** | -0.07 | 0.63 | 0.74 | **0.63** |
| Myt01-005G07 | MGC01775 | uncharacterized protein LOC100869204 isoform 2 [Apis florea] | | | | | | 0.07 | 0.30 | 0.49 | **0.30** | 0.58 | 0.77 | 0.08 | **0.58** |
| Myt01-002G11 | MGC01510 | without similarity | | | | | | 0.55 | 0.21 | 0.27 | **0.27** | 0.63 | 0.87 | 0.31 | **0.63** |
| Myt01-012F11 | MGC01731 | elongation factor 1 gamma, putative [Ixodes scapularis] | translation | | | | | 0.27 | 0.30 | 0.16 | **0.27** | 0.38 | 0.59 | 1.00 | **0.59** |
| Myt01-011C02 | MGC02430 | p8 nuclear protein [Ixodes scapularis] | replication, transcription & repair | | | | | 0.10 | 0.42 | 0.26 | **0.26** | 0.52 | 1.35 | 0.85 | **0.85** |
| Myt01-002G09 | MGC01508 | without similarity | | | | | | 0.56 | 0.26 | 0.23 | **0.26** | 0.64 | 0.08 | 0.18 | **0.18** |
| Myt01-017B11 | MGC00983 | defender against apoptotic cell death 1 [Argopecten irradians] | cell cycle & apoptosis | | | | | 0.24 | 0.26 | 0.44 | **0.26** | 1.51 | 0.74 | 1.46 | **1.46** |
| Myt01-007H10 | MGC01969 | receptor for activated C-kinase [Pinctada fucata] | signal transduction | | | | | 0.66 | 0.25 | 0.20 | **0.25** | -0.02 | 0.65 | 0.25 | **0.25** |
| Myt01-007G05 | MGC01939 | without similarity | | | | | | 0.58 | 0.23 | 0.15 | **0.23** | 0.57 | 0.14 | 0.12 | **0.14** |
| Myt01-001D03 | MGC04135 | ribosomal protein S26 [Ornithodoros parkeri] | translation | | | | | 0.31 | 0.22 | 0.13 | **0.22** | 0.30 | 0.37 | 0.35 | **0.35** |
| Myt01-012G04 | MGC02654 | microsomal glutathione S-transferase 3 [Pinctada martensi] | metabolism & ion homeostasis | | | | | 0.13 | 0.62 | 0.22 | **0.22** | 0.89 | 0.10 | 0.20 | **0.20** |
| Myt01-014G01 | MGC02914 | ribosomal protein rps27 [Arenicola marina] | translation | | | | | 0.17 | 0.41 | 0.19 | **0.19** | 0.60 | 0.72 | 0.64 | **0.64** |
| Myt01-006F10 | MGC01826 | without similarity | | | | | | 0.93 | 0.06 | 0.17 | **0.17** | 0.21 | 0.46 | 0.52 | **0.46** |
| Myt01-010E12 | MGC02332 | without similarity | | | | | | 0.16 | 0.79 | 0.10 | **0.16** | 0.66 | 0.57 | 0.69 | **0.66** |
| Myt01-017F04 | MGC01209 | ribosomal protein S24; MRP S24 [Mus musculus] | translation | | | | | -0.14 | -0.14 | -0.54 | **-0.14** | -0.22 | -0.46 | -0.28 | **-0.28** |
| Myt01-016C09 | MGC00117 | beta-microseminoprotein [Xenopus (Silurana) tropicalis] | immunity & inflammation | | | | | -0.16 | -0.18 | -0.50 | **-0.18** | -0.51 | -0.75 | -0.74 | **-0.74** |
| Myt01-015F12 | MGC01896 | without similarity | | | | | | -0.17 | -0.19 | -0.47 | **-0.19** | -0.33 | -0.47 | -0.24 | **-0.33** |
| Myt01-001A04 | MGC01325 | PACRG, partial [Xenopus laevis] | protein folding, turnover & degradation | | | | | -0.15 | -0.22 | -0.45 | **-0.22** | -0.30 | -0.48 | -0.33 | **-0.33** |
| Myt01-015A11 | MGC00019 | 6-phosphogluconolactonase-like [Strongylocentrotus purpuratus] | metabolism & ion homeostasis | | | | | -0.22 | -0.42 | -0.17 | **-0.22** | -0.45 | -0.58 | -0.63 | **-0.58** |
| Myt01-015H10 | MGC03114 | C1q domain containing protein MgC1q89 [Mytilus galloprovincialis] | immunity & inflammation | | | | | -0.23 | -0.08 | -0.52 | **-0.23** | -0.77 | -0.61 | -0.32 | **-0.61** |
| Myt01-015G01 | MGC03065 | ubiquitin [Artemia franciscana] | protein folding, turnover & degradation | | | | | -0.59 | -0.03 | -0.24 | **-0.24** | 0.69 | 0.24 | 0.57 | **0.57** |
| Myt01-016A08 | MGC00131 | zona pellucida domain protein D [Haliotis rufescens] | development & reproduction | | | | | -0.13 | -0.24 | -0.59 | **-0.24** | -0.66 | -0.69 | -0.55 | **-0.66** |
| Myt01-006A03 | MGC01810 | without similarity | | | | | | -0.24 | -0.16 | -0.44 | **-0.24** | -0.66 | -0.19 | -0.41 | **-0.41** |
| Myt01-018H06 | MGC03479 | multiple C2 and transmembrane domain-containing protein 1-like [Strongylocentrotus purpuratus] | | | | | | -0.16 | -0.24 | -0.51 | **-0.24** | -0.40 | -0.63 | -0.54 | **-0.54** |
| Myt01-015B05 | MGC02494 | without similarity | | | | | | -0.25 | -0.18 | -0.66 | **-0.25** | -0.49 | -0.71 | -0.36 | **-0.49** |
| Myt01-006G12 | MGC01838 | without similarity | | | | | | -0.10 | -0.26 | -0.37 | **-0.26** | -0.24 | -0.65 | -0.81 | **-0.65** |

| | | | | | | | | | | | |
|---|---|---|---|---|---|---|---|---|---|---|---|
| Myt01-016C12 | MGC00089 | without similarity | | -0.07 | -0.27 | -0.43 | **-0.27** | -0.41 | -0.24 | -0.29 | **-0.29** |
| Myt01-018G11 | MGC00860 | Integumentary mucin C.1 (FIM-C.1) [Xenopus laevis] | cell adhesion & extracellular matrix | -0.30 | -0.24 | -0.34 | **-0.30** | -0.24 | -0.40 | -0.30 | **-0.30** |
| Myt01-006E12 | MGC04318 | predicted protein-like [Saccoglossus kowalevskii] | | -0.16 | -0.30 | -0.34 | **-0.30** | -0.03 | -0.80 | -0.54 | **-0.54** |
| Myt01-001G04 | MGC01408 | arginine kinase [Sepiella maindroni] | metabolism & ion homeostasis | -0.31 | -0.26 | -0.46 | **-0.31** | -0.44 | -0.85 | -0.29 | **-0.44** |
| Myt01-016C11 | MGC03129 | without similarity | | -0.32 | -0.30 | -0.64 | **-0.32** | -0.50 | -0.61 | -0.53 | **-0.53** |
| Myt01-016A12 | MGC00159 | without similarity | | -0.10 | -0.33 | -0.35 | **-0.33** | -0.62 | -0.39 | -0.63 | **-0.62** |
| Myt01-017F02 | MGC01802 | collagen alpha-1(XII) chain-like [Anolis carolinensis] | cell adhesion & extracellular matrix | -0.33 | -0.01 | -0.67 | **-0.33** | -0.34 | -0.38 | -0.24 | **-0.34** |
| Myt01-017H10 | MGC03327 | without similarity | | -0.33 | -0.19 | -0.45 | **-0.33** | -0.78 | -0.55 | -0.42 | **-0.55** |
| Myt01-003G05 | MGC01606 | universal stress protein MSMEG_3950 [Clonorchis sinensis] | | -0.56 | -0.27 | -0.33 | **-0.33** | -0.56 | -0.65 | -0.39 | **-0.56** |
| Myt01-015G12 | MGC02989 | precollagen-D [Mytilus galloprovincialis] | cell adhesion & extracellular matrix | -0.34 | -0.64 | -0.04 | **-0.34** | -0.63 | -0.43 | -0.21 | **-0.43** |
| Myt01-003G12 | MGC01613 | INO80 complex subunit C-like [Danio rerio] | replication, transcription & repair | -0.16 | -0.34 | -0.40 | **-0.34** | -0.43 | -0.87 | -0.70 | **-0.70** |
| Myt01-002E12 | MGC01488 | without similarity | | -0.10 | -0.35 | -0.71 | **-0.35** | -0.45 | -0.67 | 0.10 | **-0.45** |
| Myt01-012A02 | MGC02531 | without similarity | | -0.36 | -0.26 | -0.50 | **-0.36** | -0.87 | -0.23 | -0.28 | **-0.28** |
| Myt01-013G09 | MGC02798 | without similarity | | -0.59 | -0.36 | -0.18 | **-0.36** | -0.69 | -0.34 | -0.40 | **-0.40** |
| Myt01-011H07 | MGC02521 | axonemal dynein light chain p33 [Haliotis discus discus] | cell motility & intracellular trafficking | -0.38 | -0.21 | -0.59 | **-0.38** | -0.66 | -1.09 | -0.32 | **-0.66** |
| Myt01-004B07 | MGC01642 | without similarity | | -0.12 | -0.39 | -0.46 | **-0.39** | -0.29 | -0.51 | -0.63 | **-0.51** |
| Myt01-002H11 | MGC01523 | without similarity | | -0.39 | -0.27 | -0.81 | **-0.39** | -1.28 | -1.11 | -0.38 | **-1.11** |
| Myt01-019B08 | MGC02110 | Mitochondrial-ND6 [Mytilus galloprovincialis] | metabolism & ion homeostasis | -0.21 | -0.88 | -0.41 | **-0.41** | -0.13 | -1.10 | -0.95 | **-0.95** |
| Myt01-014E05 | MGC02886 | collagen pro alpha-chain [Haliotis discus] | cell adhesion & extracellular matrix | -0.25 | -0.41 | -0.42 | **-0.41** | -0.37 | -0.89 | -0.78 | **-0.78** |
| Myt01-014A03 | MGC05861 | hypothetical protein BRAFLDRAFT_270517 [Branchiostoma floridae] | | -0.15 | -0.46 | -0.42 | **-0.42** | -0.31 | -0.84 | -0.84 | **-0.84** |
| Myt01-017B09 | MGC03196 | without similarity | | -0.42 | -0.08 | -0.81 | **-0.42** | -1.16 | -1.12 | -0.68 | **-1.12** |
| Myt01-014E06 | MGC02887 | mytimacin-4 [Mytilus galloprovincialis] | immunity & inflammation | -0.09 | -0.43 | -0.52 | **-0.43** | -0.38 | -0.48 | -0.60 | **-0.48** |
| Myt01-011E07 | MGC02476 | stress-associated endoplasmic reticulum protein 2 [Homo sapiens] | protein folding, turnover & degradation | -0.45 | -0.52 | -0.38 | **-0.45** | -0.43 | -0.45 | -0.39 | **-0.43** |
| Myt01-007F09 | MGC01920 | DEAD (Asp-Glu-Ala-Asp) box polypeptide 17 [Taeniopygia guttata] | replication, transcription & repair | -0.94 | -0.46 | -0.33 | **-0.46** | -0.45 | -0.48 | -0.45 | **-0.45** |
| Myt01-014B12 | MGC01827 | hypothetical protein BRAFLDRAFT_86469 [Branchiostoma floridae] | | -0.50 | -0.71 | -0.20 | **-0.50** | -0.29 | -0.84 | -0.08 | **-0.29** |
| Myt01-014F09 | MGC02906 | CG10903-PA [Strongylocentrotus purpuratus] | | -0.50 | -0.39 | -0.60 | **-0.50** | -0.51 | -0.76 | -0.68 | **-0.68** |
| Myt01-018G01 | MGC00020 | without similarity | | -0.85 | -0.50 | 0.00 | **-0.50** | -0.46 | -0.68 | -0.39 | **-0.46** |
| Myt01-010H11 | MGC02389 | without similarity | | -0.47 | -0.59 | -0.51 | **-0.51** | -1.10 | -0.35 | -0.32 | **-0.35** |
| Myt01-009G02 | MGC02210 | histone deacetylation protein Rxt3 [Glomerella graminicola M1.001] | replication, transcription & repair | -0.64 | -0.22 | -0.51 | **-0.51** | -0.63 | -0.78 | -0.51 | **-0.63** |
| Myt01-015H01 | MGC02998 | without similarity | | -0.74 | -0.54 | -0.38 | **-0.54** | 0.83 | 0.29 | 0.83 | **0.83** |
| Myt01-010B10 | MGC02276 | alpha 1 type XII collagen short isoform precursor [Homo sapiens] | cell adhesion & extracellular matrix | -0.59 | -2.63 | 0.34 | **-0.59** | -2.29 | 0.16 | -0.88 | **-0.88** |
| Myt01-015H12 | MGC00110 | actin [Mizuhopecten yessoensis] | cell motility & intracellular trafficking | -0.57 | -0.59 | -0.77 | **-0.59** | -0.37 | -0.72 | -0.64 | **-0.64** |
| Myt01-006H12 | MGC00243 | alpha-tubulin, partial [Nodipecten subnodosus] | cell motility & intracellular trafficking | -0.75 | -0.23 | -0.59 | **-0.59** | -0.30 | -0.90 | -0.30 | **-0.30** |
| Myt01-016D09 | MGC00176 | alpha tubulin [Pectinaria gouldii] | cell motility & intracellular trafficking | -0.34 | -0.61 | -0.63 | **-0.61** | -0.22 | -0.65 | -0.09 | **-0.22** |
| Myt01-011E05 | MGC02473 | without similarity | | -0.64 | -0.58 | -0.88 | **-0.64** | -0.72 | -0.94 | -0.03 | **-0.72** |
| Myt01-002H12 | MGC01524 | leucine--tRNA ligase, cytoplasmic-like [Strongylocentrotus purpuratus] | translation | -0.14 | -0.75 | -0.68 | **-0.68** | -0.97 | -0.06 | -0.43 | **-0.43** |
| Myt01-016D12 | MGC00084 | without similarity | | -0.57 | -0.75 | -0.71 | **-0.71** | -2.28 | -1.28 | -1.21 | **-1.28** |
| Myt01-003D04 | MGC01543 | without similarity | | 0.07 | -0.71 | -1.03 | **-0.71** | -0.24 | -0.64 | -0.57 | **-0.57** |
| Myt01-002C11 | MGC01465 | without similarity | | -0.74 | -1.36 | -0.76 | **-0.76** | -0.13 | -2.58 | -1.14 | **-1.14** |
| Myt01-013C05 | MGC02449 | nongradient byssal precursor [Mytilus edulis] | cell motility & intracellular trafficking | -0.23 | -1.29 | -0.79 | **-0.79** | -0.55 | -0.68 | -0.28 | **-0.55** |
| Myt01-011E03 | MGC02470 | without similarity | | -0.80 | -0.46 | -0.83 | **-0.80** | -1.23 | -0.25 | -0.83 | **-0.83** |
| Myt01-011C11 | MGC02439 | Q/N-rich domain Prion like protein PQN-75 (pqn-75) [Caenorhabditis elegans] | | -0.83 | -0.65 | -1.01 | **-0.83** | -0.05 | -0.60 | -0.94 | **-0.60** |
| Myt01-003E07 | MGC00175 | beta tubulin [Chlamys farreri] | cell motility & intracellular trafficking | -1.04 | -0.71 | -0.87 | **-0.87** | -0.82 | -1.12 | -0.87 | **-0.87** |
| Myt01-013C10 | MGC01399 | without similarity | | -1.74 | -0.43 | -1.42 | **-1.42** | -0.39 | -0.73 | -0.90 | **-0.73** |
| Myt01-007H08 | MGC01966 | inhibitor of apoptosis 1 [Gallus gallus] | cell cycle & apoptosis | -1.27 | -1.71 | -1.65 | **-1.65** | -1.13 | -1.83 | -0.92 | **-1.13** |
| Myt01-008B03 | MGC02005 | without similarity | | -1.69 | -0.88 | -2.08 | **-1.69** | -1.90 | -1.07 | -1.58 | **-1.58** |
