# Supplementary material for: DNA Damage and Transcriptional Changes in the Gills of Mytilus galloprovincialis Exposed to Nanomolar Doses of Combined Metal Salts (Cd, Cu, Hg)
Source: PLoS One. 2013 Jan 23;8(1):e54602. doi: 10.1371/journal.pone.0054602 (PMC3552849; doi:10.1371/journal.pone.0054602)
Supplement: Table S6 — Relative quantification values (RQ) of selected mussel transcripts significantly modulated after exposure to the combined metals. Pooled gill RNAs from both treated and control mussels were tested in triplicate according to the 2−ΔΔCt method and considering 18S rRNA as endogenous control gene. (PDF) [file pone.0054602.s008.pdf]

Table S6. Relative quantification values (RQ) of selected mussel transcripts significantly modulated after exsposure to the combined metals.

Metal mixture (nM)	Transcript	RQ	±SD
50	MT10	0.79	0.19
100	MT10	2.31	0.37
200	MT10	3.03	0.27
50	MT20	4.97	0.59
100	MT20	8.84	3.17
200	MT20	17.19	1.87
50	small HSP24.1	11.49	2.71
100	small HSP24.1	58.04	3.53
200	small HSP24.1	30.33	2.87
50	SQSTM1	16.87	2.21
100	SQSTM1	21.49	2.56
200	SQSTM1	29.00	2.78
50	HSC70	3.26	0.76
100	HSC70	3.26	0.69
200	HSC70	3.72	0.84
50	HSP90	3.38	0.75
100	HSP90	4.09	0.75
200	HSP90	6.48	1.06
50	ferritin	2.50	0.50
100	ferritin	2.76	0.90
200	ferritin	6.81	1.77
50	GADD45 gamma	6.05	0.82
100	GADD45 gamma	5.80	1.06
200	GADD45 gamma	10.82	2.45

Pooled gill RNAs from both treated and control mussels were tested in triplicate according to the $2^{-\Delta\Delta Ct}$ method and considering to 18S rRNA as endogenous control gene.
